# Case-Control Study of the Association between Single Nucleotide Polymorphisms of Genes Involved in Xenobiotic Detoxification and Antioxidant Protection with the Long-Term Influence of Organochlorine Pesticides on the Population of the Almaty Region

**DOI:** 10.3390/toxics11120948

**Published:** 2023-11-21

**Authors:** Nazym Altynova, Ozada Khamdiyeva, Aleksandr Garshin, Gulminyam Baratzhanova, Almira Amirgaliyeva, Akerke Seisenbayeva, Gulnar Abylkassymova, Kanagat Yergali, Anar Tolebaeva, Liliya Skvortsova, Gulnur Zhunussova, Bakhytzhan Bekmanov, Céline Cakir-Kiefer, Leyla Djansugurova

**Affiliations:** 1 Institute of Genetics and Physiology, Al-Farabi Avenue 93, Almaty 050060, Kazakhstan; naz10.79@mail.ru (N.A.); garshin1511@gmail.com (A.G.); almira-71@mail.ru (A.A.); s_akerke@mail.ru (A.S.); gulyam05@mail.ru (G.A.); ergali.0394@mail.ru (K.Y.); anara_tolebaeva@mail.ru (A.T.); lilia_555@rambler.ru (L.S.); gulnur_j@mail.ru (G.Z.); bobekman@rambler.ru (B.B.); 2 Faculty of Biology and Biotechnology, Al-Farabi Kazakh National University, Al-Farabi Avenue 71, Almaty 050040, Kazakhstan; 3 INRAE, UR AFPA, USC 340, University of Lorraine, Nancy F-54000, France; celine.cakir-kiefer@univ-lorraine.fr

**Keywords:** pesticides, organochlorine pesticides, case-control study, xenobiotic detoxification genes, antioxidant protection genes, SNP, public health

## Abstract

The association of genetic polymorphisms with the individual sensitivity of humans to the action of pesticide pollution is being actively studied in the world. The aim of this study was a molecular epidemiological analysis of candidate polymorphisms of genes involved in pesticide metabolism, detoxification, and antioxidant protection. Some of the selected polymorphisms also relate to susceptibility to cancer and cardiovascular, respiratory, and immune system diseases in individuals exposed to pesticides for a long time. For a case-control study of a unique cohort of people exposed to organochlorine pesticides for 10 years or more were chosen, a control cohort was selected that matched with the experimental group by the main population characteristics. PCR-PRLF and genome-wide microarray genotyping (GWAS) methods were used. We identified 17 polymorphisms of xenobiotic detoxification genes and 27 polymorphisms of antioxidant defense genes, which had a significantly high statistical association with the negative impact of chronic pesticide intoxication on human health. We also found 17 polymorphisms of xenobiotic detoxification genes and 12 polymorphisms of antioxidant defense genes that have a protective effect. Data obtained added to the list of potential polymorphisms that define a group at high risk or resistant to the negative effects of pesticides.

## 1. Introduction

Pesticides are a special group of chemical compounds that are deliberately introduced into the environment to protect crops and control pests (insects and rodents) and weeds. They are used as plant growth regulators, and play an important role in food processing and storage in the commercial and food processing industries [1,2]. It is well known that the use of pesticides affects ecosystems and human health. Pesticide usage induces disorders of the reproductive, endocrine, nervous, and immune systems, and causes cancer, allergies, respiratory diseases, and birth defects [3,4]. Although the WHO (World Health Organization) and FAO (Food and Agriculture Organization of the United Nations) together developed an international code for the management of pesticides, even today, a significant part of the population is influenced by direct and indirect pesticide pollution [5].

Pesticide exposure is an important public health problem in many developing countries dependent on agriculture [3]. In this context, agricultural workers, their families, and individuals living near fields where pesticides are applied are the group receiving the most significant exposure and with a higher risk of adverse health effects than people living in urban areas [6].

The adverse effect of pollutants depends on the dose, chemical properties, and exposure time. Usually, it is much more difficult to indicate the character of exposure to low concentrations of pesticides as either chronic for a long time or a single case of acute toxic influence. The effect of chronic exposure to low doses of pesticides may not appear for several months or even years.

According to data from the Ministry of Agriculture of the Republic of Kazakhstan (July 2012), about 6931.4 tons of obsolete, banned, and unusable pesticides are stored in warehouses in various regions of the republic (http://www.greenwomen.kz/pdf/stok.pdf) (accessed on 24 January 2019). On the territory of 10 districts of the Almaty region, 64 pesticide storage facilities are now ownerless. They accumulated 68,443 kg of obsolete and unusable pesticides of sym-triazine, organophosphorus, chlorine-containing, fluorine-containing, and thiocarbamate classes, as well as pesticides of German and Chinese origin [7]. It is known that these pesticides are highly toxic substances with pronounced skin-resorptive toxicity and immunotoxicity, and can have mutagenic, antimitotic, and embryotoxic effects [4]. Most pesticides that enter the body undergo biotransformation under the influence of enzymes, the activity of which is controlled by the genes of the xenobiotic detoxification system [8]. These genes’ polymorphisms determine the allelic states and genotypes corresponding to proteins with different functional activities [9]. Xenobiotic detoxification genes are involved in the pathogenesis of various cancer types and act as modifiers and risk factors in various diseases associated with the adverse effects of environmental factors [4,10]. 

It is known that some pesticides widely used in agriculture disrupt electron transport chains of mitochondria and endoplasmic reticulum, and inhibit cellular enzymes associated with antioxidants or their biosynthesis, thereby increasing the formation of reactive oxygen species (ROS) and causing oxidative stress [11]. Changes in the physiological balance lead to excess oxidants and serious damage to cellular components and macromolecules, especially DNA [12].

Thus, when studying the effect of pesticides that increase ROS production, it is necessary to consider variations in the genes’ functions in regulating the cell cycle, redox status, and drug metabolism. It is known that genetic polymorphisms of many enzymes involved in these processes negatively affect the biotransformation and detoxification of pesticides and can lead to the development of acute or chronic diseases, especially in subjects already exposed to other pollutants [13]. This allows the exploration of the mechanisms of the occurrence of environmentally conditioned pathology depending on the characteristics of the genotype, and offers specific solutions aimed at protecting the health of people and their offspring [9].

So, genes for the detoxification of xenobiotics and the antioxidant system play an important role in individual sensitivity to environmental toxic agents. Many of them are characterized by genetic polymorphism that affects the functional activity of their alleles. There is evidence that the null allele of glutathione-S-transferase, which has an extended deletion, is associated with lung cancer, bladder cancer, breast cancer, and the development of endometriosis [14]. Many case-control studies revealed the associations of single nucleotide polymorphisms (SNPs) of genes participating in xenobiotic detoxification and antioxidant protection with a broad spectrum of multifactorial diseases. 

This study is part of the results of a complex project devoted to evaluating the ecological situation of populated territories of the Almaty region contaminated by banned organochlorine pesticides over 10 years. The results indicated that organochlorine pesticides and their decay products entered the human food chain and influenced the level of chromosome aberrations and somatic health status [15,16,17,18,19,20]. The impact of many factors on health status was estimated in a recently published article [19]. The pesticide content in the food chain was a primary factor influencing health status. Single nucleotide polymorphisms of xenobiotic detoxification and antioxidant defense genes were also assessed as significant factors. We suppose that “unfavorable” genotypes on genes involved in protection against pesticides can promote the development of many diseases in exposed populations, increasing individual lethality. Chronic pesticide exposure can influence the selection process, inducing the adaptive response. Considering the environment’s ecological state, the frequency of “favorable” genotypes in exposed populations can be increased compared to nonexposed populations. As a result, we decided to expand the range of studied SNPs and conduct a case-control study using a population cohort from “free of pesticides” territories as a control.

Focusing on the medical status of the population exposed to pesticide contamination (cardiovascular diseases, gastrointestinal diseases, cancer, autoimmune diseases, diabetes mellitus, hypothyroidism, etc.), we chose SNPs of xenobiotic detoxification genes (*CYP1A1*, *CYP2B6*, *CYP2D6*, *CYP2C19*, *GSTM1*, *GSTT1*, *GSTP1*, *NAT2*) [14,21,22] and antioxidant protection (*GCLC*, *GCLM*, *GPX4*, *PON1*, *PON2*, *PON3*, *NQO1*, *SOD1*, *SOD2*, *SOD3*, *AKR1B10*, *AKR1C1*, *APOE*, *NFE2*, *NFE2L1*, *NFE2L2*, *NFE2L3*, *SRXN1*, *TXNRD1*, *UCP3*) [9,13,23,24,25,26,27], mutations which are associated with these diseases. Trying to answer the question of whether SNPs of xenobiotic detoxification genes and the genes of the antioxidant system influence the development of different diseases in selected populations that are chronically exposed to organochlorine pesticides, we conducted a case-control study to assess the health implications of these SNPs. 

## 2. Materials and Methods

### 2.1. Study Objects

The study was approved by the local ethical commissions (Protocol No. 52 dated 5 September 2017 of the local ethical committee of the Kazakh–Russian Medical University and Protocol No. 6 dated 7 December 2020 of the local ethical committee of the Institute of Human and Animal Physiology), which allowed for further analysis of the population living in areas where non-utilized pesticides are prohibited for use, and conditionally healthy people.

The selection of a study cohort was carried out taking into account previous studies in the frame of the project “Comprehensive Assessment of the Impact of Non-utilized and Banned Pesticides on the Genetic Status and Health of the Population of the Almaty Region” (BR05236379, 2018–2020). Collected biosamples are presented in the gene bank of the Institute of Genetics and Physiology (Almaty, Kazakhstan) [17,18,19]. The materials for this study were personal data, general health survey data, and EDTA-treated frozen peripheral blood samples. For molecular genetic analysis, 221 residents of the Almaty region were selected (collection of biomaterials of 2018–2020—v. Beskaynar, v. Kyzylkairat, v. Belbulak, v. Amangeldy, v. Enbekshi; collection of 2021—v. Karakestek, v. Umbetali, v. Akdala) [18]. The main selection criteria were living for more than 10 years near sources of pesticide contamination. Our previous studies showed that people in these areas consumed food contaminated with pesticides. In total, 24 organochlorine pesticides and their derivates were analyzed in food products. All pesticides were divided into six groups depending on the chemical structure of active compounds: group 1 DDT (4.4 DDT; 4.4 DDD; 2.4 DDD; 4.4 DDD, DDE); group 2 hexahalogenated benzene (HCB) (hexachlorobenzene and hexabromobenzene); group 3 hexachlorocyclohexane (HCH)(α-HCH, γ-HCH, β-HCH, δ-HCH); group 4 aldrin (aldrin, endrin, dieldrin, endrin aldehyde); group 5 (endosulfan: endosulfan 1, endosulfan 2, and endosulfan sulfate); and group 6 heptachlor (heptachlor and heptachlor epoxide). Measured concentrations of pesticides in food were compared to European Union and Custom Union standards (EU—Pesticides database, 2009; Unified sanitary and epidemiological and hygienic requirements for products, 2010) [17].

**Figure 1:**
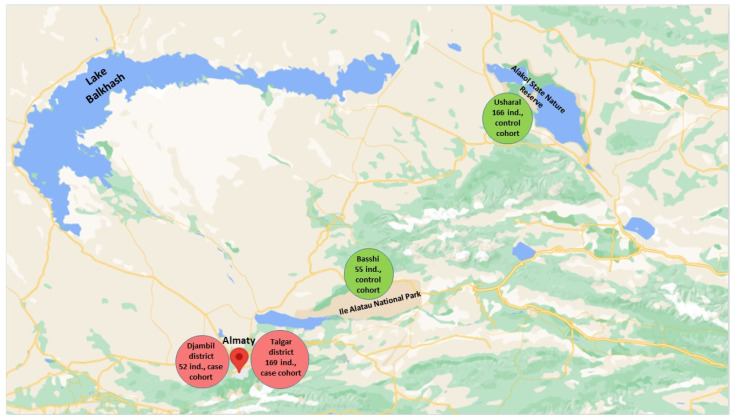
The location of the territories of the Talgar and Dzhambul districts of the Almaty region, in which the study volunteers (case—red color and control—green color) live.

Two settlements of the Almaty region were selected for collection samples for the control group (v. Basshi—55 individuals and v. Usharal—166 individuals) (Figure 1). These villages are located on the territory of national natural reserves. Previously (2021), it had been shown that soil and water samples from these territories were not contaminated by pesticides [18]. 

### 2.2. DNA Extraction

Genomic DNA was isolated from frozen (−20 °C) peripheral blood samples containing EDTA as an anticoagulant agent. DNA extraction was performed using a commercial Wizard^®^ Genomic DNA Purification Kit (Promega, Madison, WI, USA) according to the protocol recommended by the manufacturer. Qualitative and quantitative characteristics of DNA samples were assessed on a NanoDrop OneC spectrophotometer (Thermo Fisher Scientific, Waltham, MA, USA) and a Qubit fluorometer (Thermo Fisher Scientific, Waltham, MA, USA).

### 2.3. Genotyping by PCR-RFLP Method

For genotyping SNPs that were not provided by the Infinium Global Screening Array, 24 kit v3.0 panel, site-specific PCR followed by an analysis of restriction fragment length polymorphism (RFLP) were used. Specific primers were designed for each polymorphism using the available online PrimerQuest (https://eu.idtdna.com/PrimerQuest/Home/Index) (accessed on 24 April 2023), “Basic Local Alignment Search Tool (primer-BLAST)” (https://www.ncbi.nlm.nih.gov/tools/primer-blast/index.cgi?LINK_LOC=BlastHome) (accessed on 24 April 2023), developed by the National Center for Biotechnology Information (NCBI). The main criteria for selecting primers were the absence of hairpin formation, which could interfere with amplification, and the annealing temperature. A critical condition for multiplex PCR was matching the annealing temperature of the primers used in one reaction. The deletion polymorphism of the *GSTT1* and *GSTM1* genes was determined in the multiplex PCR mode. Amplification of the β-globin gene fragment was used as an internal control. Primers, PCR conditions, restriction endonucleases, and restriction products corresponding to the homozygous and heterozygous state of polymorphic variants compared with the wild type are presented in Table 1.

PCR was performed on a Mastercycler (Eppendorf, Taufkirchen, Germany). The reaction mixture included 25–50 ng of genomic DNA, 10 pM of each primer, and 10 µL of PCR Master Mix (2×) (Thermo Fisher Scientific, Waltham, MA, USA). The length of the amplified products was analyzed on 1% agarose gel. The PCR reaction conditions were optimally selected for each pair of primers, considering their base melting point, concentration in the reaction mixture, and the DNA polymerase used. Genotyping was performed in triplicate to avoid genotyping errors.

Further identification of the studied single nucleotide substitutions was carried out by hydrolysis of the polymorphic site in the amplified DNA fragments with the corresponding restriction endonucleases, which were selected using the WatCut online program (Table 2) (http://diyhpl.us/~bryan/irc/protocol-online/protocol-cache/template.php) (accessed on 22 October 2023). Restriction products were analyzed in 8% polyacrylamide gel and visualized using a gel-documenting system Quantum STS (Vilber Lourmat, Collégien, France).

### 2.4. Genome-Wide Genotyping of SNPs and Bioinformatic Processing

Microarray SNP genotyping was performed using the Infinium Global Screening Array 24 kit v3.0 on the iScan Illumina platform using the Infinium HTS Automated protocol [28,29]. A total of 654,270 SNPs were genotyped. Row microarray genotyping data were processed using Illumina GenomeStudio v.2.05, software (Illumina, San Diego, CA, USA), PLINK [30], RStudio (RStudio Team (2020). RStudio: Integrated Development for R. RStudio, PBC, Boston, MA. URL http://www.rstudio.com/) (accessed on 10 June 2022). Samples that had call rates of less than 98% were excluded from the analysis. Bioinformatic analysis of the results of genome-wide SNP genotyping included determining the data reliability, determining the genetic status—homozygosity/heterozygosity—for the identified SNPs, and the detection and analysis of the pathogenicity of the identified mutations and polymorphisms by comparing the found mutations with the mutations identified as pathogenic in known databases and literature sources. Annotation and interpretation of genetic variants obtained using iScan was carried out using the Genome-Wide Association Studies data catalog (GWAS catalog—https://www.ebi.ac.uk/gwas/) (accessed on 24 April 2023), the Single Nucleotide Polymorphisms Database (dbSNP, https://ncbi.nlm.nih.gov/snp/) (accessed on 24 April 2023), ClinVar (https://ncbi.nlm.nih.gov/clinvar/) (accessed on 24 April 2023), and the 1000 Genomes database (1000G— https://www.ensembl.org/info/website/tutorials/grch37.html) (accessed on 24 April 2023).

To study the effect of polymorphisms on pesticide-mediated health effects, SNPs of eight xenobiotic biotransformation genes (*CYP1A1*, *CYP2B6*, *CYP2D6*, *CYP2C19*, *GSTM1*, *GSTT1*, *GSTP1*, *NAT2*) and 20 antioxidant defense genes (*GCLC*, *GCLM*, *GPX4*, *PON1*, *PON2*, *PON3*, *NQO1*, *SOD1*, *SOD2*, *SOD3*, *AKR1B10*, *AKR1C1*, *APOE*, *NFE2*, *NFE2L1*, *NFE2L2*, *NFE2L3*, *SRXN1*, *TXNRD1*, *UCP3*) were chosen.

In total, 608 candidate SNPs of genes participating in xenobiotic detoxification and antioxidant defense were analyzed regarding the heterozygosity/homozygosity, frequencies of alleles, and genotypes in case and control cohorts. Ninety-eight homozygous variants by ancestral alleles in both case and control cohorts were excluded from further analysis, as well as 15 repeated SNPs.

### 2.5. Statistical Methods

Conventional methods of variation statistics were used to assess the fit of case and control cohorts on key population characteristics such as age, gender, ethnicity, and bad habits. The significance level (P) was determined using Chi2 (χ2) and Student’s *t*-test. Differences were regarded as significant at *p* < 0.05, and for matching— *p* > 0.95. Compliance with the Hardy–Weinberg equilibrium (χ2) was assessed in all cases of SNP genotyping. The association of the polymorphism of the studied xenobiotic detoxification genes and antioxidant genes with diseases was evaluated using the odds ratio (OR) method [31]. For OR, the level of significance (p) was determined using the Chi2 (χ2) criterion. The calculation was carried out separately for allelic variants and genotypes, taking into account the general, dominant (heterozygous carriers and homozygotes for a rare allele were combined into one group), and recessive models (heterozygous carriers and homozygotes for a common allele were combined into one group).

## 3. Results

### 3.1. Characteristics of Case-Control Cohorts

As a result of previous studies by the Institute of Genetics and Physiology, a unique genetic bank has been created, representing the population that has lived near pesticide contamination sites for a long time. Surveys of these individuals included questioning, clinical and cytogenetic factors, the level of consumption of pesticides with products of plant and animal origin, etc. [17,19]. Based on these materials, a case cohort of 221 individuals (Supplementary Materials Tables S1 and S2) was selected for the molecular epidemiological study. The control cohort was chosen from the population living in ecologically favorable areas on the territories of the National Natural Parks, where the land was not treated by pesticides. When selecting volunteers, the studies were guided by an approximate correspondence with the personal data of the case cohort people according to individual parameters, such as ethnicity, age, gender, profession, type of house, and bad habits. Selected data from questionnaires relevant to this study are presented in Supplementary Materials Tables S1 and S2.

The main population characteristics of case and control cohorts are represented in Table 3.

Thus, the case-control cohorts were well-matched for key population characteristics such as age, sex, and smoking. In terms of ethnicity, Kazakhs were significantly prevalent in both cohorts.

**Figure 2:**
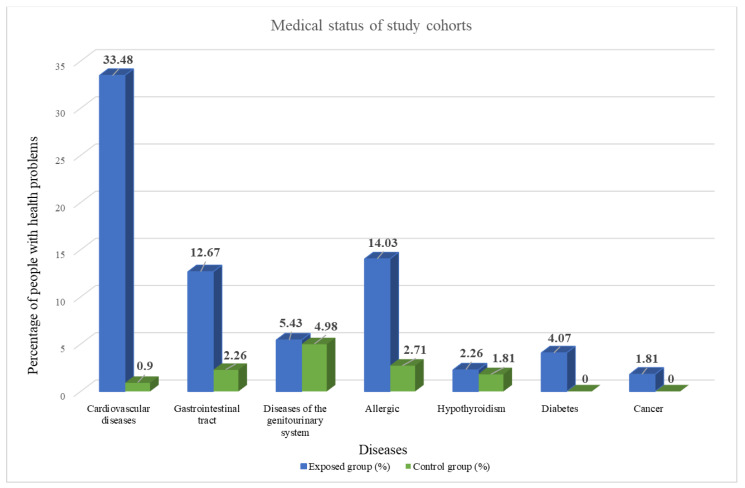
Medical status in a cohort of individuals chronically exposed to pesticide contamination and corresponding control cohort from ecologically favorable environments.

Five years had passed since the time of material collection, and a second survey of the case cohort in 2023 was conducted for health reasons. The updated data show that in the group of people exposed to pesticides, 73.75% of the inhabitants were found to have various diseases; in particular, 33.48% were cardiovascular diseases, 12.67% were gastrointestinal diseases, 14.03% were allergic diseases, 5.43% were diseases of the genitourinary system, 2.26% were hypothyroidism, 4.07% were diabetes mellitus, and 1.81% were cancer. In the control group, 12.66% of the subjects had various diseases (Figure 2).

**Figure 3:**
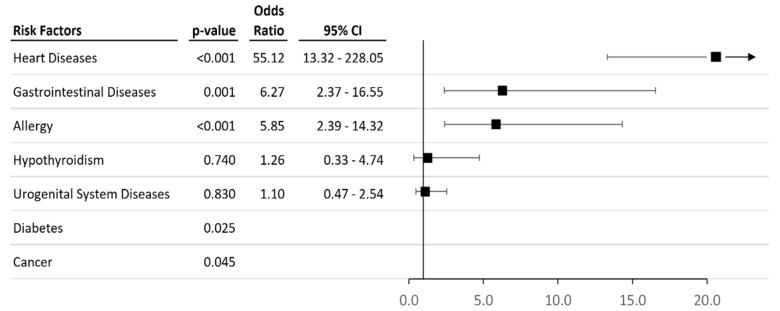
Forest plot of groups of diseases in case and control cohorts.

An associative analysis of medical status in cohorts of individuals exposed to pesticide contamination of the food chain and individuals from ecologically clean areas revealed significant associations of an increase in the incidence of cardiovascular diseases (OR = 55.12, 95%CI = 13.32–228.05), diseases of the gastrointestinal tract (OR = 6.27, 95%CI = 2.37–16.55) and allergic diseases (OR = 5.85, 95%CI = 2.39–14.32) in the case cohort. Regarding hypothyroidism and diseases of the genitourinary system, the prevalence of cases of these diseases in the case cohort did not show significant differences. Cases of diabetes and cancer were reported only in the case cohort (Figure 3).

Thus, the excess of cardiovascular, gastrointestinal tract, and allergic disease frequencies in people chronically exposed to organochlorine pesticides in the food chain can be considered an important criterion for selecting a control group for molecular epidemiological study.

### 3.2. Genotyping Results

The case and control cohorts were genotyped on 608 candidate SNPs of genes participating in xenobiotic detoxification (eight genes) and antioxidant protection (20 genes) using the PCR-RLFP method and genome-wide microarray genotyping on the iScan platform. Two deletion polymorphisms of *GSTT1* and *GSTM1* genes were genotyped using multiplex PCR. After excluding homozygous variants (98) and repeated SNPs (15), a total number of 496 SNPs and two deletion polymorphisms were analyzed, which represented genes of the cytochrome P450 family (*CYP1A1*, *CYP2B6*, *CYP2D6*, *CYP2C19—251 SNPs*), glutathione-S-transferases (*GSTM1*, *GSTT1*, *GSTP1—19 SNPs* and two deletion polymorphisms), genes of N-acetyltransferase 2 (*NAT2—36 SNPs*), the glutathione-cysteine ligase gene family (*GCLC*, *GCLM—26 SNPs)*, the glutathione peroxidase 4 gene (*GPX4*,—*4 SNPs*); the superoxide dismutase gene family (mitochondrial isoforms *SOD1*, *SOD2,* extracellular isoform *SOD3*—*37 SNPs*), the human paraoxonase gene family (*PON1*, *PON2, PON3—40 SNPs*)*,* genes of the aldo-keto reductase family (*AKR1B10*, *AKR1C1*—*8 SNPs*)*,* gene of apolipoprotein E (*APOE 16 SNPs*)*,* genes of *NFE2*-like transcription factor (*NFE2*, *NFE2L1*, *NFE2L2*, *NFE2L3—15 SNPs*), the gene of sulfiredoxin-1 (*SRXN1—3 SNPs*)*,* NADP quinone dehydrogenase 1 gene (*NQO1—14 SNPs),* the gene of thioredoxin reductase 1 (*TXNRD1*—*14 SNPs*), and ubiquitin specific peptidase 3 (*UCP3—13 SNPs*). Out of 496 SNPs, 202 SNPs represent intronic variants and regulatory regions, and 294 SNPs are exon variants. Among the 294 exon variant SNPs, 213 represent missense, eight nonsense, 40 synonymous, and 33 silencing variants.

The correspondence to the Hardy–Weinberg equilibrium was determined to genotype distribution for all genotyped candidate polymorphisms. According to the data of intragroup analysis, in the case cohort, the distribution of genotypes by 221 SNPs, and in the control cohort by 366 SNPs, corresponded to the Hardy–Weinberg equilibrium (*p* > 0.05). Complete data on the frequencies of identified alleles and the distribution of genotypes in the control and case cohorts are given in Supplementary Materials Tables S3 and S4. 

Below, we present selected data on frequencies of determined xenobiotic detoxification (Table 4) and antioxidant protection (Table 5) genes’ SNPs alleles and genotypes. 

In the general cohort, determined frequencies of allelic variants of genes involved in the biotransformation of organochlorine pesticides and antioxidant protection were at the expected levels for populations of mixed Asian–European origin. Regarding the deletion polymorphism of the glutathione-S-transferase T1 and M1 types genes, a relatively high frequency of deletions (>0.500) was noted, which characterized both the case and control cohorts.

### 3.3. Analysis of Associations between the Allelic and Genotype Variants and Chronic Pesticide Exposure

The method of odd ratio estimation was applied for the case and control cohorts to assess the possible association between candidate polymorphisms of genes participating in xenobiotic detoxification and antioxidant protection. Data on 498 polymorphisms related to 28 candidate genes are represented in Supplementary Materials (Tables S3 and S4). We excluded the variants that did not correspond to the Hardy–Weinberg equilibrium and variants where some genotypes were absent or presented by single cases. 

**Figure 4:**
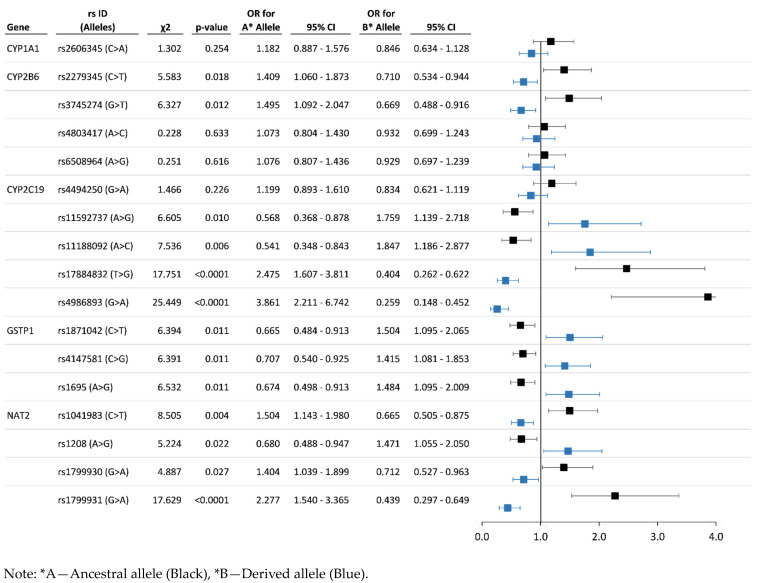
Chronic pesticide exposure risk associated with the most significant allele variants of genes participating in xenobiotic detoxification.

**Figure 5:**
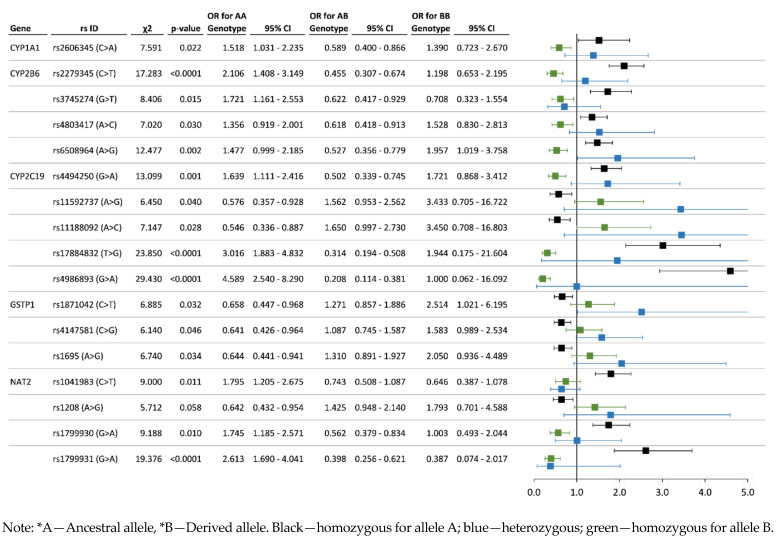
Chronic pesticide exposure risk associated with genotype variants of genes participating in xenobiotic detoxification.

**Figure 6:**
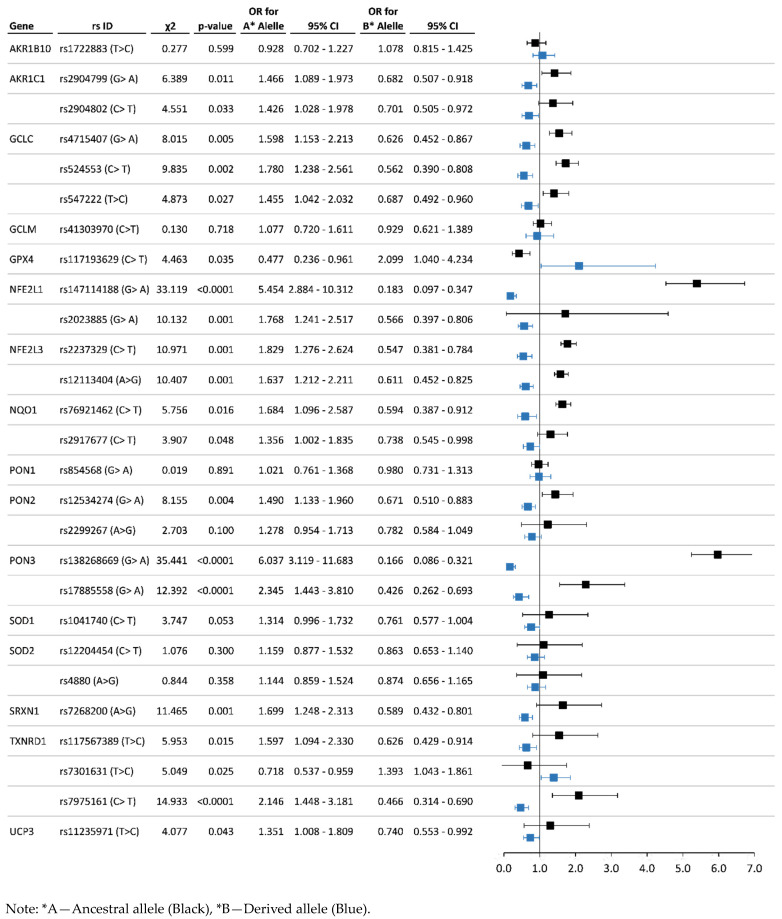
Chronic pesticide exposure risk associated with the most significant allele variants of antioxidant system genes.

**Figure 7:**
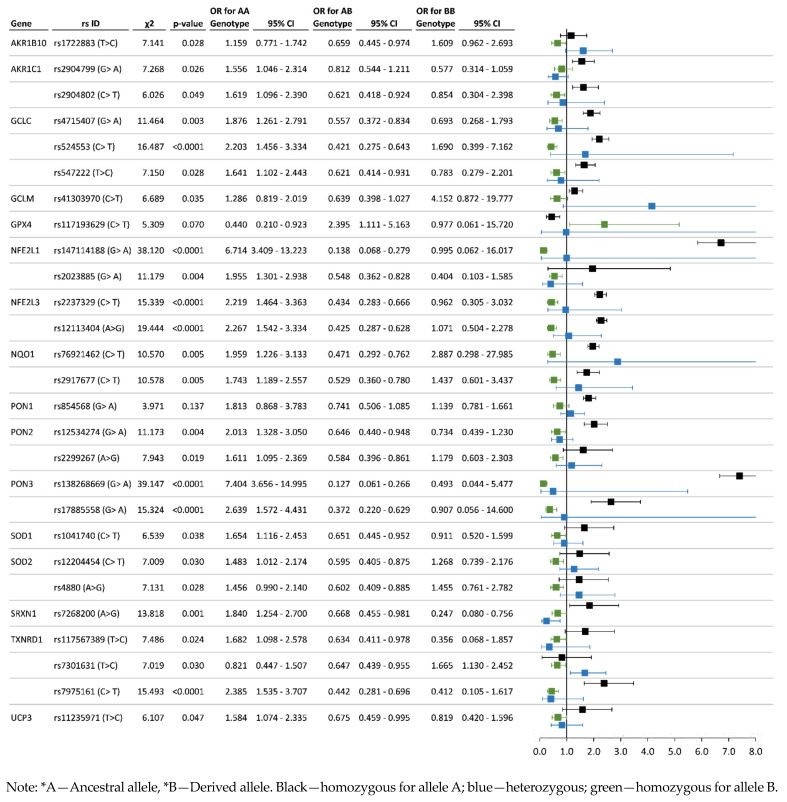
Chronic pesticide exposure risk associated with genotype variants of antioxidant system genes.

Association analysis results can be used to detect the risk (OR ˃ 1.2, *p* < 0.01) and protective (OR < 0.80, *p* < 0.01) alleles and genotypes. Regarding the system of xenobiotic detoxification, 17 SNPs of five genes (*CYP1A1*, *CYP2B6*, *CYP2C19*, *GSTP1*, *NAT2*) revealed a statistically significant association with chronic exposure to organochlorine pesticides. For studied polymorphisms of antioxidant system genes, 27 SNPs of 16 genes (*AKR1B10*, *AKR1C1*, *GCLC, GCLM, GPX4*, *NFE2L1*, *NFE2L3*, *NQO1*, *PON1*, *PON2*, *PON3*, *SOD1*, *SOD2*, *SRXN1*, *TXNRD1*, *UCP3*) have shown the most significant associations. The statistics on associations of polymorphisms of genes participating in xenobiotic detoxification are represented in Figure 4 and Figure 5, and data for allelic and genotype variants of antioxidant system genes are illustrated in Figure 6 and Figure 7, respectively. 

Case-control analysis of SNPs of genes encoding the enzymes of cytochrome P450-dependent monooxygenases family revealed 10 polymorphisms that have a significantly high risk for the pesticide-polluted cohort: *CYP1A1*—rs2606345 (AA genotype—OR = 1.52; CC genotype—OR = 1.39); *CYP2B6*—rs2279345 (TT—OR = 2.11), rs3745274 (TT—OR = 1.72), rs4803417 (AA—OR = 1.36; CC—OR = 1.53), rs6508964 (AA—OR = 1.48; GG—OR = 1.96); *CYP2C19*—rs4494250 (AA—OR = 1.64; GG—OR = 1.72), rs11592737 (AG—OR = 1.56; GG—OR = 3.43), rs11188092 (AC—OR = 1.65; CC—OR = 3.45), rs17884832 (TT—OR = 3.02; GG—OR = 1.94), rs4986893 (AA—OR = 4.59). Corresponding protective effect of genes of the cytochrome P450-dependent monooxygenases family: *CYP1A1*—rs2606345 (AC—OR = 0.59); *CYP2B6*—rs2279345 (TC—OR = 0.46), rs3745274 (TG—OR = 0.62; GG—OR = 0.71), rs4803417 (AC—OR = 0.62), rs6508964 (AG—OR = 0.53); *CYP2C19*—rs4494250 (AG—OR = 0.50), rs11592737 (AA—OR = 0.58), rs11188092 (AA—OR = 0.55), rs17884832 (TG—OR = 0.31), rs4986893 (AG—OR = 0.21). Among genes of the glutathione-S transferase family, the relative risk and protective effect were detected for three SNPs of the *GSTP1* gene—rs1695 (risk genotypes AG—OR = 1.54 and GG—OR = 1.54, protective genotype AA—OR = 0.66), rs1871042 (risk genotypes TC—OR = 1.27 and CC—OR = 2.51, protective genotype—TT—OR = 0.66), rs4147581 (risk genotype CC—OR = 1.58, protective genotype GG—OR = 0.64). Gene encoding N-acetyltransferase 2 revealed significant associations for four SNPs: *NAT2*—rs1041983 (risk genotype TT—OR = 1.80, protective genotypes TC—OR = 0.74 and CC—OR = 0.65), rs1208 (risk genotypes AG—OR = 1.43 and GG—OR = 1.80, protective genotype AA—OR = 0.64), rs1799930 (risk genotype AA—OR = 1.75, protective genotype AG—OR = 0.56), rs1799931 (risk genotype AA—OR = 2.61, protective genotypes AG—OR = 0.40 and GG—OR = 0.39).

In addition to risk alleles and genotypes, we identified a number of polymorphic genotypes of protective significance (OR < 0.8). The complete list of significantly protective SNPs is presented in Supplementary Materials Tables S3 and S4. Among the SNPs of genes encoding the cytochrome P450-dependent monooxygenases family, there are the AC genotype (OR = 0.59, *p* = 0.022) of rs2606345 *CYP1A1* gene; *CYP2B6* gene—rs2279345 TC genotype (OR = 0.46, *p* < 0.001), rs3745274 (TG—OR = 0.62; GG—OR = 0.71, *p* = 0.015), rs4803417 (AC—OR = 0.62, *p* = 0.030), rs6508964 (AG—OR = 0.53, *p* = 0.002); *CYP2C19* gene—rs4494250 (AG—OR = 0.50, *p* = 0.001), rs11592737 (AA—OR = 0.58, *p* = 0.040), rs11188092 (AA—OR = 0.55, *p* = 0.028), rs17884832 (TG—OR = 0.31, *p* < 0.001), rs4986893 (AG—OR = 0.21, *p* < 0.001). Among the genes of the glutathione-S transferase family, there are some protective genotypes of the *GSTP1* gene: AA (OR = 0.66, *p* = 0.013) by rs1695, TT (OR = 0.66, *p* = 0.032) by rs1871042, GG (OR = 0.64, *p* = 0.046) by rs4147581. Gene encoding N-acetyltransferase 2 revealed the associations of the protective effect from pesticide pollution for four SNPs: *NAT2*—rs1041983 (protective genotypes TC—OR = 0.74 and CC—OR = 0.65, *p* = 0.011), rs1208 (AA genotype—OR = 0.64, *p* = 0.058), rs1799930 (AG genotype—OR = 0.56, *p* = 0.010), rs1799931 (genotypes AG—OR = 0.40 and GG—OR = 0.39, *p* < 0.001).

Among the antioxidant protection genes, the most significant protective effect was detected for the following genotypes: *NFE2L1*—rs147114188 (AG—OR = 0.14, *p* < 0.001) and rs2023885 (AG—OR = 0.55 and GG—OR = 0.40, *p* = 0.004); *NFE2L3*—rs2023885 (TC—OR = 0.43, *p* < 0.001) and rs12113404 (AG—OR = 0.43, *p* < 0.001); *NOQ1*—rs76921462 (TC—OR = 0.43, *p* = 0.005), rs2917677 (TC—OR = 0.53, *p* = 0.005); *PON2*—rs2299267 (AG—OR = 0.58, *p* = 0.019); *PON3*—rs138268669 (AG—OR = 0.13, *p* < 0.001) and rs17885558 (AG—OR = 0.37, *p* < 0.001); *SRXN1*—rs7268200 (GG—OR = 0.25, *p* = 0.001); *TXNRD1*—rs117567389 (CC—OR = 0.36, *p* = 0.024) and rs7975161 (GC—OR = 0.44 and CC—OR = 0.41, *p* < 0.001).

## 4. Discussion

### 4.1. Background for Control Cohort Selection 

Populations of people regularly exposed to low doses of pesticides for a long time are of great interest to the study of individual susceptibility and resistance to intoxication by pesticides. As mentioned above (Section 2), a unique biobank of frozen peripheral blood (−20 °C) samples of a population living in pesticide-contaminated areas for more than 10 years has been collected, stored, and studied at the Institute of Genetics and Physiology of Almaty (Kazakhstan). Our previous results showed that organochlorine pesticides and their breakdown products enter the human food chain and affect the level of chromosomal aberrations and physical health [17]. The population’s health status was characterized by a range of typical diseases with a prevalence of cardiovascular diseases. A recently published article assessed the impact to health and genetic status of many risk factors, including individual genotypes of the most popular polymorphisms of genes involved in xenobiotic detoxification, DNA repair, and antioxidant defense. A significant effect was recorded for the group of xenobiotic detoxification genes (26%). The influence of genes on the antioxidant defense system was mainly expressed in genetic risk (8%) but did not significantly affect health risk (0.03%). It should be noted that only the most popular polymorphisms of genes involved in xenobiotic detoxification and antioxidant protection were previously studied [19]. The hypothesis of an adaptive response that assumes the rapid realization of “unfavorable” genotypes through the development of fatal diseases (for example, cancer) and the selection of “favorable” protective genotypes under chronic intoxication by pesticides is quite testable on a population that has been exposed to banned organochlorine pesticides for 15 years. This molecular epidemiological case-control study was conducted on the same experimental group living in areas contaminated with pesticides for 10–15 years. Because initially only a small range of well-known gene polymorphisms was determined in this population, to better understand the influence of genes of xenobiotic detoxification and antioxidant protection on the individual health and genetic state of the population affected by pesticides, we significantly expanded the panel of studied polymorphisms and chose the control population for comparison of chances. The control cohort was selected among the population living in pesticide-clear areas of this region, matching the experimental group in individual age, gender, ethnicity, lifestyle, and bad habits. Thus, the case and control cohorts had a similar climatic–geographical, social, and ethnic background. 

However, for individual health states, in populations living in contaminated areas, data on cardiovascular and gastrointestinal tract diseases, and allergies significantly exceeded the corresponding indicators of the control cohort. The most common group of diseases in the case cohort were cardiovascular diseases (ischemic heart disease, hypertension), the occurrence of which was noted at a relatively early age (the average age of patients was 57.45 ± 9.88). Our earlier studies on cardiovascular diseases showed that the early age of manifestation of cardiovascular diseases in the population of Almaty and the Almaty region was 60 years [32].

It has been shown that more than 60% of diseases and deaths associated with environmental pollution were cardiovascular diseases, such as chronic coronary heart disease, heart attack, stroke, and heart rhythm disorders (arrhythmias) [33,34]. Maria Tvermosegaard and coauthors detected that cardiovascular diseases are the most serious problem for the indigenous population of the Arctic who consume pesticides with seafood. Very high concentrations of a wide range of pesticides have been recorded in adult Eskimos, including pregnant women, newborns, and fetuses [35]. Based on the results of these studies and the frequency of occurrence of cardiovascular diseases in the study groups, it can be concluded that one of the causes of the disease in the study group was contamination of the territory and human food chain by pesticides, which, in combination with unfavorable genotypes regarding the detoxification (*CYP1A1*, *CYP2B6*, *CYP2C19*) and antioxidant protection (*GCLC*, *GCLM*, *GPX4*, *NFE2L1*, *NQO1*, *PON1*, *PON2*, *SOD1*, *SOD2*, *SRXN1*, *TXNRD1*, *UCP3*) functions, promoted the development of cardiovascular disease.

In our cohort, people also had asthma and asthmatic bronchitis. According to the literature data, many pesticides are sensitizers or irritants that can directly damage the bronchial mucosa, making the airways very sensitive to allergens or other irritants that could increase the risk of asthma developing [36,37].

Not many studies related to pesticides’ impact on gastrointestinal tract diseases exist. However, many studies were devoted to the effects of pesticides on the microbiome and pancreatic cancer [38]. In our cohort, there were pancreas, gallbladder, and liver disease cases among people with gastrointestinal problems. Studies have shown that people with pesticide poisoning may have subclinical signs of acute pancreatitis [39,40]. 

The consequences of chronic pesticide exposure can lead to the development of various types of cancer because of these substances’ genotoxic and mutagenic potential [13]. There were four cancer cases in our cohort exposed to pesticides.

This study’s case-control analysis of the association between the occurrence of the main groups of diseases and chronic pesticide intoxication (see Figure 2) showed a relative risk for cardiovascular diseases (OR = 55.12, *p* < 0.001), diseases of the gastrointestinal tract (OR = 6.27, *p* = 0.001), and allergic diseases (OR = 5.85, *p* < 0.001). This confirms that people exposed to pesticides are more at risk of developing the abovementioned diseases than those not in contact with pesticides.

### 4.2. Detoxification Genes

The influence of genetic polymorphisms on the mechanisms of pesticide biotransformation is currently relevant to understanding pesticide metabolism and induction of diseases better. Molecular epidemiological studies of genetic polymorphisms detected the involvement of xenobiotic detoxification and antioxidant protection genes in developing a wide range of multifactorial diseases, including oncological, cardiovascular, neurodegenerative, and autoimmune diseases [4,26,27]. Data indicate the possibility of using polymorphic variants of xenobiotic metabolism genes as molecular genetic markers, which show the individual sensitivity of the population to adverse environmental influences [21]. 

The genotyping results of 608 polymorphisms in the case and control cohorts showed that the frequencies of allelic variants of genes involved in the biotransformation of organochlorine pesticides and antioxidant protection were at the expected levels for populations of mixed Asian–European origin.

Polymorphisms of *CYP* genes (*CYP1A1*, *CYP1A2*, *CYP1B1*, *CYP2B6*, *CYP2C9*, *CYP3A4*, *CYP2D6* and *CYP2C19)* and the *PON1* gene have been actively studied in connection with the development of a wide range of oncological, cardiovascular, and allergic diseases, and also in populations professionally exposed to pesticides [13,41,42,43,44,45]. 

Among all ethnic groups, Asians have a higher frequency of individuals who poorly metabolize xenobiotics due to *CYP2C19* function [46]. Exposure to organochlorine pesticides can provoke inhibition of this enzyme. For example, in vitro experiments have shown that the chiral pesticide Ethofumesate strongly inhibits *CYP2C19* [47]. Another pesticide, Chlorpyrifos, can be activated as a potent β-esterase inhibitor of CYP2B6 or detoxified to dearylated metabolites by CYP2C19 [48]. However, the intensity of oxidation of cytochrome P-450 enzymes often turns an inactive substrate into a chemically reactive, toxic metabolite [49]. 

According to our data, the *CYP*-family gene activity differs between the case and control cohorts. An association study revealed a significantly high risk for five SNPs of the *CYP2C19* gene: rs4494250, rs11592737, rs11188092, rs17884832, and rs4986893. The association with chronic pesticide intoxication was detected for four single nucleotide polymorphisms of the *CYP2B6* gene: rs2279345, rs3745274, rs4803417, and rs6508964. The increased risk was determined for AA (OR = 1.52) and CC (OR = 1.39) genotypes of the rs2606345 *CYP1A1* gene (*p* = 0.022). Based on these data, we consider it possible to include these SNPs in other epidemiological studies as markers of pesticide sensitivity. 

In epidemiological studies, the most studied is the deletion polymorphism of the glutathione S-transferase (GST) superfamily genes involved in the second phase of xenobiotic detoxification. Enzymes of the GST family have a high degree of polymorphism, which determines different individual sensitivities of organisms to the action of mutagenic factors, including pesticides and hereditary predisposition to multifactorial diseases. Deletion mutation of the *GSTT1* and *GSTM1* genes may be a risk factor for developing various types of cancer [22,24] and other chronic diseases associated with delayed exposure to genotoxicants. According to the published data, the GST gene alleles’ frequencies vary depending on the geographical region and ethnicity [50].

The data obtained from studied cohorts revealed a high frequency of glutathione-S-transferase T1 and M1 gene deletions in both the case and control cohorts. The frequency of deletion of the *GSTT1* gene in the ethnically mixed populations of the Almaty region (case cohort—0.640, control cohort—0.658) was much higher than the frequencies characteristic of Asian populations (0.260–0.460), European populations (0.220), and African populations (0.380). Deletions of the GSTM1 gene in the cohorts of the population of the Almaty region (case—0.707, control cohort—0.683) occurred with a very high frequency that significantly exceeded the frequency of deletions of this gene, determined for many Asian (0.430–0.530), European (0.520), African (0.300–0.550), and Native American (0.330) populations. 

In East Asians, higher frequencies of null genotypes were observed in healthy volunteers than in patients who suffered from diseases [14]. Likely, the high frequency of deletions of the *GSTT1* and *GSTM1* genes is typical for the population of Kazakhstan. In a case-control study of *GSTM1* and *GSTT1* deletion polymorphisms’ role in fetal growth restriction due to exposure to organochlorine pesticides, it was noticed that organochlorine pesticides and oxidative stress were associated with adverse reproductive outcomes [51]. Other studies found correlations between pesticide exposure, specific pathologies, and nonfunctional *GST* genotypes [52]. 

In this study, we did not determine a statistically significant relative risk for homozygotes for *GSTT1* and *GSTM1* gene deletions because of the high frequency of *GST* deletions in both the case and control cohorts. Perhaps selection is an important issue in chronic pesticide intoxication. Many years of exposure to pesticide-contaminated environments possibly promote individuals’ GST deletions to develop fatal diseases faster. Thus far, two cancer patients from the cohort exposed to pesticides have already died; one of them was a heterozygous carrier of the *GSTM1* gene deletion, and the other was homozygous for the *GSTM1* deletion (sample codes UZH 15 and UZH 37).

In relation to another member of the *GST* family, the *GSTP1* gene, a statistically significant high relative risk was determined for three SNPs: rs1695 for AG genotype (OR = 1.54) and GG (OR = 1.54), *p* = 0.013), rs1871042 for TC (OR = 1.27) and CC (OR = 2.51), *p* = 0.032), rs4147581 for CC (OR = 1.58, *p* = 0.046). In addition, in a case-control study, SNPs of gene encoding N-acetyltransferase 2 revealed the risk associations with pesticide contamination: *NAT2*—rs1041983, rs1208, rs1799930, rs1799931. This also highlights the decreasing xenobiotic detoxification enzymes’ functional activity in a population chronically exposed to pesticides.

### 4.3. Antioxidant System Genes

Pesticide-induced oxidative stress may involve critical pathophysiological mechanisms leading to the development of cancer and cardiovascular pathology, and may cause immunosuppression and stimulate the development of neurodegenerative diseases [53,54,55,56]. 

Thus, it has been shown that workers handling organochlorine pesticides have a reduced function of the enzyme superoxide dismutase [53]. Variations in the *SOD2* and *SOD3* genes can participate in susceptibility to breast cancer, chronic obstructive pulmonary disease in adults and children, preeclampsia, insulin resistance, type 2 diabetes, and arterial hypertension [54,56,57].

The glutathione peroxidase enzyme GSH-Px4, due to its unique properties of reducing lipid hydroperoxides, plays an important role in the prevention of pesticide accumulation in the body, protecting against development of cardiovascular diseases [25], renal failure in patients with type 1 diabetes mellitus [58], schizophrenia [59], and chronic obstructive pulmonary disease [60]. Modification in glutathione synthesis regarding the function of the *GCLM* gene can be involved in pesticides binding, and following excretion from the cells, preventing the development of a wide spectrum of diseases [61,62,63].

The human paraoxonase (PON) gene family encodes enzymes that catalyze organophosphates before they can phosphorylate cholinergic enzymes. Polymorphism of paraoxygenase genes is associated with the development of a wide range of diseases induced by oxidative stress [64,65,66,67,68,69,70,71]. A number of studies have shown the relationship between the work of PON enzymes and the toxicity of pesticides [64,65].

For several SNPs of other antioxidant system genes (*TXNRD-1*, *GPX4*, *NQO1*, *NFE2L3*, *NFE2L1*) that prevent the formation of free radicals and reactive oxygen molecules, thereby protecting the cell from oxidative stress, the significant associations with various cancer types and various skin, respiratory system, and digestive tract pathologies [72,73] have been detected. We can assume the participance of these genes in pesticide metabolism. However, in this regard, there are no data on populations exposed to pesticides.

In the present study of a population exposed to chronic intoxication by organochlorine pesticides, we showed the possible involvement of polymorphisms in a wide range of antioxidant system genes (*GCLC*, *GCLM*, *GPX4*, *PON1*, *PON2*, *PON3*, *NQO1*, *SOD1*, *SOD2*, *SOD3*, *AKR1B10*, *AKR1C1*, *APOE*, *NFE2*, *NFE2L1*, *NFE2L2*, *NFE2L3*, *SRXN1*, *TXNRD1*, *UCP3*) in public health problems, which was expressed in the increasing frequencies of cardiovascular pathologies, gastrointestinal tract diseases, and the development of allergic diseases [9,27,73,74,75,76]. This range of polymorphisms (*SOD1* rs1041740; *SOD2* rs12204454, rs4880; *GCLM* rs41303970; *GCLC* rs4715407, rs524553, rs547222; *GPX4* rs117193629; *PON1* rs854568; *PON2* rs12534274, rs2299267; *PON3* rs138268669, rs17885558; *TXNRD1* rs117567389, rs7301631, rs7975161; *NQO1* rs76921462, rs2917677; *NFE2L1* rs2237329; *NFE2L3* rs12113404; *AKR1B10* rs1722883) that revealed the relative risk in our study (Figure 4, Figure 5, Figure 6 and Figure 7) can complement the panel of prognostic markers for the health of populations exposed to pesticides 

For most studied SNPs, the heterozygous genotypes revealed a protective effect. Considering the significant difference between the control population and the cohort chronically exposed to organochlorine pesticides in the frequency of occurrence of a large number of allelic variants for many SNPs, it can be assumed that evolutionary fixation of “favorable” genotypes occurs in a pesticide-polluted environment. This suggests that, over the course of evolution, the fixation of these SNPs in populations probably reflected the need to adapt to persistent pesticide intoxication [77]. For example, a study by David Lozano-Paniagua and coauthors assessed a range of biomarkers of oxidative stress (*TBARS*, *FRAS*, and *SHT*) and antioxidant enzymes (*GGT* and *PON1*) as part of an integrated production system in greenhouse workers from southeast Spain engaged in intensive agriculture, regularly exposed to various pesticides. The results of this study indicate an immoderate increase in oxidative stress levels associated with pesticide exposure, followed by an adaptive response aimed at increasing antioxidant protection [78]. Interestingly, the effect of selective selection is confirmed by analyzing individual genotypes. Thus, individuals with good health indicators from the case cohort are characterized by many “favorable” genotypes, while “unfavorable” gene variants are widely represented in sick individuals.

Thus, detected associations of polymorphisms of xenobiotic detoxification genes and antioxidant protection genes can expand the range of predictive health markers for populations exposed to pesticides. We believe that the associations found in our case-control study will be beneficial in assessing the effects of pesticides, helping to identify risk groups, and preventing the development of a wide range of diseases in the population receiving pesticides through the food chain.

## 5. Conclusions

Overall, our studies identified 17 polymorphisms in xenobiotic detoxification genes and 27 polymorphisms in antioxidant defense genes, which had a significant statistical association with the negative impact of chronic pesticide intoxication on human health. We also found 17 polymorphisms in xenobiotic detoxification genes and 12 polymorphisms in antioxidant defense genes that have a protective effect. The results of this case-control study still need to be confirmed by studies of xenobiotic detoxification and antioxidant protection genes pointed to SNPs in other pesticide-exposed populations, as well as by in vitro experiments to analyze the expression and functional activity of the corresponding polymorphic enzymes. The obtained data adds to the list of potential polymorphisms that define a group at high risk or resistant to the adverse effects of pesticides.

## Figures and Tables

**Table 1:** Primers and PCR conditions.

Gene	SNPs	Primer, 5′→3′	PCR Conditions	PCR Products Length
*GPX4*	Leu220Leu	f–GAGAAGGACCTGCCCCACTA r–GTCATGAGTGCCGGTGGAAG	95 °C—4 min. 95 °C—30 s. 61 °C—30 s. 35 cycles 72 °C—45 s. 72 °C—5 min.	96 bp
*GCLC*	−129T/C	f–TCGTCCCAAGTCTCACAGTC r–CGCCCTCCCCGCTGCTCCTC	95 °C—4 min. 95 °C—30 s. 61 °C—30 s. 35 cycles 72 °C—45 s. 72 °C—5 min.	613 bp
*GCLM*	−588C/T	f–CTCAAGGGCAAAGACTCA r–CCGCCTGGTGAGGTAGACAC	95 °C—4 min. 95 °C—30 s. 58 °C—30 s. 35 cycles 72 °C—45 s. 72 °C—5 min.	329 bp
*GSTP1*	Ile105Val	f–ACCCCAGGGCTCTATGGGAA r–TGAGGGCACAAGAAGCCCCT	94 °C—4 min. 94 °C—30 s. 55 °C—30 s. 30 cycles 72 °C—30 s. 72 °C—8 min.	176 bp
*GSTT1*	Deletion	f–CCTTACTGGTCCTCACATCTC r–TCACCGGATCATGGCCACA	94 °C—4 min. 94 °C—4 min. 59 °C—1 min. 35 cycles 72 °C—1 min. 72 °C—8 min.	+/+;+/−: 480 bp
*GSTM1*	Deletion	f–GAACTCCCTGAAAAGCTAAAGC r–GTTGGGCTCAAATATACGGTGG		+/+;+/−: 215 bp
*β*-globin		f–CAACTTCATCCACGTTCACC r–GAAGAGCCTAGGACAGGTAC		+/+: 268 bp

**Table 2:** Restriction endonucleases for RFLP-analysis, restriction product length, and corresponding SNP genotypes.

Gene/rs	Substitutions	Restriction Endonucleases	Genotypes and DNA Fragments Length (bp)
*GPX4* (rs713041)	Leu220Leu	StyI	TT: 68 + 28; TC: 96 + 68 + 28; CC: 96
*GCLC* (rs524553)	−129T/C	Tsp45I	CC: 500 + 113; CT: 500 + 302 + 198 + 113; TT: 302 + 198 + 113
*GCLM* (rs41303970)	−588C/T	MspI	CC: 200 + 84 + 45; CT: 200 + 129 + 84 + 45; TT: 200 + 129
*GSTP1* (rs1695)	Ile105Val	Alw26I	AA: 91 + 85; AG: 176 + 91 + 85; GG: 176

**Table 3:** Characteristics of case-control cohorts.

Characteristics		Case, %	Control, %	t_st_	*p*-Value
**N**		221	221		
Age (years)		1939–2004 (51.60 ± 12.26)	1940–1987 (54.16 ± 7.68)	0.178	0.899
Sex, n (%)	Males	70 (31.67)	70 (31.67)	0	1 ***
	Females	151(68.33)	151(68.33)	0	1 ***
Ethnicity, n (%)	Kazakhs	184 (83.26)	185 (83.71)	0.052	0.967 *
	Russians	23 (10.41)	19 (8.60)	0.610	0.651
	Other nationalities	14 (6.33)	17 (7.69)	0.533	0.688
Smoking, n (%)	Smokers	40(18.09)	37 (16.74)	0.270	0.832
	Non-smokers	181 (81.91)	184 (83.26)	0.262	0.837

Note: Matching confidence *—*p* ≥ 0.95; *** *p* ≥ 0.999.

**Table 4:** Allele and genotype frequencies on selected detoxification gene variants in case and control cohorts.

Gene	rs ID	CASE					CONTROL					European/Asian Frequencies
		Allele Frequencies		Genotype Frequencies			Allele Frequencies		Genotype Frequencies			
		A*	B*	AA	AB	BB	A*	B*	AA	AB	BB	
*CYP1A1*	rs2606345 (C > A)	0.690	0.310	0.491	0.398	0.111	0.653	0.347	0.388	0.529	0.083	C = 0.37 A = 0.63 C = 0.95 A = 0.05
*CYP2B6*	rs6508964 (A > G)	0.671	0.329	0.479	0.382	0.138	0.654	0.346	0.384	0.540	0.076	C = 0.62 T = 0.33 C = 0.67 T = 0.33
	rs3745274 (G > T)	0.775	0.225	0.606	0.338	0.056	0.697	0.303	0.472	0.451	0.078	G = 0.716 T = 0.284 G = 0.815 T = 0.185
	rs4803417 (A > C)	0.669	0.331	0.477	0.384	0.139	0.653	0.347	0.402	0.503	0.095	C = 0.41 A = 0.58 C = 0.34 A = 0.66
	rs2279345 (C > T)	0.680	0.320	0.484	0.392	0.124	0.601	0.399	0.308	0.586	0.106	C = 0.62 T = 0.33 C = 0.67 T = 0.33
*CYP2C19*	rs4494250 (G > A)	0.710	0.290	0.535	0.350	0.115	0.671	0.329	0.412	0.518	0.070	G = 0.65 A = 0.34 G = 0.83 A = 0.17
	rs11592737 (A > G)	0.857	0.143	0.747	0.221	0.032	0.913	0.087	0.837	0.154	0.010	A = 0.78 G = 0.22 A = 0.77 G = 0.23
	rs4986893 (G > A)	0.961	0.039	0.926	0.069	0.005	0.863	0.137	0.731	0.264	0.005	G = 0.9998 A = 0.0002 G = 0.974 A = 0.026
	rs11188092 (A > C)	0.859	0.141	0.751	0.217	0.032	0.919	0.081	0.847	0.144	0.010	A = 0.81 C = 0.19 A = 0.99 C = 0.02
	rs17884832 (T > G)	0.922	0.078	0.853	0.138	0.009	0.826	0.174	0.657	0.338	0.005	T = 0.93 G = 0.06 T = 0.81 G = 0.19
*GSTP1*	rs1871042 (C > T)	0.723	0.277	0.526	0.395	0.079	0.797	0.203	0.627	0.340	0.033	C = 0.66 T = 0.33 C = 0.82 T = 0.18
	rs4147581 (C > G)	0.512	0.488	0.270	0.484	0.247	0.597	0.403	0.366	0.463	0.171	C = 0.49 G = 0.51 C = 0.32 G = 0.68
	rs1695 (A > G)	0.695	0.305	0.482	0.427	0.091	0.772	0.228	0.591	0.363	0.047	A = 0.67 G = 0.33 A = 0.81 G = 0.19
*NAT2*	rs1799930 (G > A)	0.749	0.251	0.577	0.344	0.079	0.680	0.320	0.438	0.483	0.079	G = 0.715 A = 0.285 G = 0.678 A = 0.322
	rs1799931 (G > A)	0.898	0.102	0.806	0.185	0.009	0.795	0.205	0.613	0.363	0.024	G = 0.974 A = 0.026 G = 0.890 A = 0.110
	rs1208 (A > G)	0.750	0.250	0.560	0.380	0.060	0.815	0.185	0.665	0.300	0.034	G = 0.434 A = 0.566 G = 0.247 A = 0.753
	rs1041983 (C > T)	0.647	0.353	0.433	0.429	0.138	0.550	0.450	0.299	0.502	0.199	C = 0.689 T = 0.311 C = 0.578 T = 0.422

Note: A*—Ancestral allele, B*—Derived allele.

**Table 5:** Allele and genotype frequencies on selected antioxidant gene variants in case and control cohorts.

Gene	rs ID	CASE					CONTROL					European>Asian Frequencies
		Allele Frequencies		Genotype Frequencies			Allele Frequencies		Genotype Frequencies			
		A*	B*	AA	AB	BB	A*	B*	AA	AB	BB	
*AKR1B10*	rs1722883 T > C	0.576	0.424	0.369	0.415	0.217	0.594	0.406	0.335	0.518	0.147	T = 0.54 C = 0.46 T = 0.58 C = 0.42
*AKR1C1*	rs2904799 A > G	0.712	0.288	0.519	0.386	0.095	0.628	0.372	0.410	0.436	0.154	G = 0.960 A = 0.040 G = 0.71 A = 0.29
	rs2904802 T > C	0.813	0.188	0.657	0.310	0.032	0.752	0.248	0.542	0.420	0.038	T = 0.333 C = 0.667 T = 0.192 C = 0.808
*GCLC*	rs4715407 A > G	0.804	0.196	0.645	0.318	0.037	0.720	0.280	0.492	0.455	0.052	G = 0.864 A = 0.136 G = 0.736 A = 0.264
	rs524553 T > C	0.871	0.129	0.765	0.212	0.023	0.791	0.209	0.596	0.390	0.014	C = 0.76 T = 0.24 C = 0.85 T = 0.15
	rs547222 T > C	0.816	0.184	0.664	0.304	0.032	0.753	0.247	0.546	0.413	0.041	T = 0.942 C = 0.059 T = 0.74 C = 0.26
*GCLM*	rs41303970 C > T	0.881	0.119	0.799	0.164	0.037	0.873	0.127	0.756	0.235	0.009	G = 0.833 A = 0.167 G = 0.812 A = 0.188
*GPX4*	rs117193629 T > C	0.942	0.058	0.889	0.106	0.005	0.972	0.028	0.948	0.047	0.005	C = 0.95 T = 0.05 C = 0.98 T = 0.01
*NFE2L1*	rs2023885 A > G	0.856	0.144	0.726	0.260	0.014	0.771	0.229	0.575	0.391	0.034	G = 0.895 A = 0.105 G = 0.768 A = 0.232
	rs147114188 A > G	0.972	0.028	0.949	0.046	0.005	0.866	0.134	0.736	0.259	0.005	G = 0.9998 A = 0.0002 G = 0.991 A = 0.009
*NFE2L3*	rs12113404 A > G	0.769	0.231	0.606	0.324	0.069	0.670	0.330	0.405	0.530	0.065	A = 0.890 G = 0.110 A = 0.674 G = 0.326
	rs2237329 T > C	0.866	0.134	0.760	0.212	0.028	0.780	0.220	0.589	0.383	0.029	C = 0.989 T = 0.011 C = 0.962 T = 0.037
*NQO1*	rs2917677 T > C	0.757	0.243	0.574	0.366	0.060	0.697	0.303	0.436	0.521	0.043	C = 0.57 T = 0.43 C = 0.90T = 0.09
	rs76921462 T > C	0.910	0.090	0.833	0.153	0.014	0.857	0.143	0.718	0.277	0.005	C = 0.977 T = 0.023 C = 0.9997 T = 0.0003
*PON1*	rs854568 A > G	0.295	0.705	0.097	0.396	0.507	0.291	0.709	0.056	0.470	0.474	G = 0.199 A = 0.801 G = 0.303 A = 0.697
*PON2*	rs12534274 A > G	0.628	0.372	0.400	0.456	0.144	0.531	0.469	0.249	0.565	0.187	G = 0.756 A = 0.244 G = 0.516 A = 0.484
	rs2299267 A > G	0.717	0.283	0.530	0.373	0.097	0.664	0.336	0.412	0.505	0.083	A = 0.83 G = 0.16 A = 0.68 G = 0.32
*PON3*	rs17885558 A > G	0.938	0.063	0.880	0.116	0.005	0.865	0.135	0.735	0.260	0.005	G = 0.993 A = 0.007 G = 0.964 A = 0.036
	rs138268669 A > G	0.973	0.027	0.952	0.043	0.005	0.859	0.141	0.727	0.263	0.010	G = 0.9998 A = 0.0002 G = 0.9997 A = 0.0003
*SOD1*	rs1041740 T > C	0.655	0.345	0.435	0.440	0.125	0.591	0.409	0.318	0.547	0.136	C = 0.644 T = 0.356 C = 0.602 T = 0.398
*SOD2*	rs12204454 T > C	0.663	0.337	0.484	0.358	0.158	0.629	0.371	0.387	0.484	0.129	C = 0.80 T = 0.20 C = 0.65 T = 0.35
	rs4880 A > G	0.687	0.313	0.488	0.396	0.115	0.657	0.343	0.396	0.522	0.082	A = 0.50 G = 0.50 A = 0.86 G = 0.13
*SRXN1*	rs7268200 A > G	0.788	0.212	0.594	0.387	0.018	0.686	0.314	0.443	0.486	0.071	A = 0.671 G = 0.329 A = 0.747 G = 0.253
*TXNRD1*	rs117567389 T > C	0.867	0.133	0.744	0.246	0.009	0.804	0.196	0.634	0.340	0.026	T = 0.9996 C = 0.0004 T = 1 C = 0
	rs7301631 T > C	0.291	0.709	0.102	0.377	0.521	0.363	0.637	0.122	0.483	0.395	T = 0.49 C = 0.51 T = 0.22C = 0.78
	rs7975161 T > C	0.898	0.102	0.810	0.176	0.014	0.804	0.196	0.642	0.325	0.033	T = 0.159 C = 0.841 T = 0.140 C = 0.860
*UCP3*	rs11235971 T > C	0.714	0.286	0.512	0.406	0.083	0.649	0.351	0.398	0.502	0.100	T = 0.847 C = 0.153 T = 0.697 C = 0.303

Note: A*—Ancestral allele, B*—Derived allele.

## Data Availability

The data presented in this study are available on request from the corresponding author. The data are not publicly available due to the privacy protection of people who participated in this study.

## Supplementary Materials

The following supporting information can be downloaded at: https://www.mdpi.com/article/10.3390/toxics11120948/s1. Table S1: Case cohort data; Table S2: Control cohort data; Table S3: Allele frequency and odds ratio of studied SNPs; Table S4: Association between individual SNPs and pesticide exposure; Table S5: Allele frequency of significantly associated polymorphisms of xenobiotic detoxification genes; Table S6: Genotype frequency of significant associations of xenobiotic detoxification genes; Table S7: Allele frequency of significantly associated polymorphisms of antioxidant defense genes; Table S8: Genotype frequency of significant associations of antioxidant defense genes.
