# Supplementary material for: Case-Control Study of the Association between Single Nucleotide Polymorphisms of Genes Involved in Xenobiotic Detoxification and Antioxidant Protection with the Long-Term Influence of Organochlorine Pesticides on the Population of the Almaty Region
Source: Toxics. 2023 Nov 21;11(12):948. doi: 10.3390/toxics11120948 (PMC10747153; doi:10.3390/toxics11120948)
Supplement: Supplementary file 1 [file toxics-11-00948-s001.zip › Supplementary Materials 2.pdf]

Table S3. Allele frequency and odds ratio of studied SNPs

Gene	rs ID	Alleles	Allele frequency in case		Allele frequency in control		χ2	*p*-value	OR for A* allele	95% CI	OR for B* allele	95% CI
			Allele A*	Allele B*	Allele A*	Allele B*						
AKR1B10	rs77337210	A>G	0.995	0.005	0.969	0.031	7.431	0.006	6.318	1.391-28.702	0.158	0.035-0.719
AKR1B10	rs1722883	T>C	0.576	0.424	0.594	0.406	0.277	0.599	0.928	0.702-1.227	1.078	0.815-1.425
AKR1C1	rs113645163	T>G	0.991	0.009	0.986	0.014	0.386	0.534	1.493	0.418-5.328	0.670	0.188-2.39
AKR1C1	rs117795769	A>G	0.993	0.007	0.889	0.111	41.004	<0.0001	17.507	5.395-56.814	0.057	0.018-0.185
AKR1C1	rs142200840	A>C	0.993	0.007	0.889	0.111	42.018	<0.0001	17.861	5.51-57.904	0.056	0.017-0.182
AKR1C1	rs2904799	A>G	0.712	0.288	0.628	0.372	6.389	0.011	1.466	1.089-1.973	0.682	0.507-0.918
AKR1C1	rs2904802	T>C	0.813	0.188	0.752	0.248	4.551	0.033	1.426	1.028-1.978	0.701	0.505-0.972
AKR1C1	rs7076886	T>C	0.820	0.180	0.828	0.172	0.087	0.768	0.949	0.668-1.347	1.054	0.743-1.496
APOE	rs150375400	A>G	0.993	0.007	0.991	0.009	0.135	0.713	1.324	0.295-5.951	0.755	0.168-3.395
APOE	rs11083750	A>C	1.000	0.000	0.993	0.007	3.024	0.082	-	-	-	-
APOE	rs11083750.1	G>C	1.000	0.000	0.993	0.007	3.011	0.083	-	-	-	-
APOE	rs140808909	A>G	0.995	0.005	0.901	0.099	38.730	<0.0001	23.406	5.628-97.341	0.043	0.01-0.178
APOE	rs190853081	A>G	0.991	0.009	0.995	0.005	0.681	0.409	0.495	0.09-2.719	2.019	0.368-11.083
APOE	rs199768005	A>T	0.998	0.002	0.995	0.005	0.349	0.555	2.033	0.184-22.506	0.492	0.044-5.445
APOE	rs201672011	A>G	0.995	0.005	0.991	0.009	0.690	0.406	2.028	0.37-11.134	0.493	0.09-2.706
APOE	rs267606661	C>G	1.000	0.000	0.995	0.005	2.043	0.153	-	-	-	-
APOE	rs267606664	A>G	0.993	0.007	0.989	0.011	0.477	0.490	1.651	0.392-6.953	0.606	0.144-2.55
APOE	rs28931577	A>G	0.993	0.007	0.991	0.009	0.144	0.704	1.336	0.297-6.007	0.748	0.166-3.363
APOE	rs387906567	T>C	0.995	0.005	0.993	0.007	0.178	0.673	1.469	0.244-8.833	0.681	0.113-4.096
APOE	rs7412	T>C	0.935	0.065	0.955	0.045	1.692	0.193	0.674	0.37-1.226	1.485	0.816-2.702
APOE	rs7412_10AT	T>C	0.937	0.063	0.958	0.042	1.945	0.163	0.649	0.352-1.197	1.541	0.836-2.843
APOE	rs7412_15AT	T>C	0.949	0.051	0.964	0.036	1.186	0.276	0.690	0.353-1.35	1.449	0.741-2.833
APOE	rs769449	A>G	0.876	0.124	0.894	0.106	0.703	0.402	0.836	0.549-1.272	1.197	0.786-1.822
APOE	rs769455	T>C	0.995	0.005	0.991	0.009	0.662	0.416	2.000	0.364-10.978	0.500	0.091-2.744
CYP1A1	rs1799814	T>G	0.988	0.012	0.914	0.086	24.358	<0.0001	7.763	3.004-20.061	0.129	0.05-0.333
CYP1A1	rs143070677	C>G	0.998	0.002	0.990	0.010	1.912	0.167	4.164	0.463-37.413	0.240	0.027-2.158
CYP1A1	rs17861084	T>G	0.993	0.007	0.958	0.042	11.107	0.001	6.311	1.835-21.7	0.158	0.046-0.545
CYP1A1	rs17861094	A>G	1.000	0.000	0.998	0.002	1.006	0.316	-	-	-	-
CYP1A1	rs180744198	T>C	0.995	0.005	0.894	0.106	42.706	<0.0001	25.622	6.178-106.254	0.039	0.009-0.162
CYP1A1	rs201691396	A>C	1.000	0.000	0.897	0.103	46.806	<0.0001	-	-	-	-
CYP1A1	rs2278970.2	G>C	1.000	0.000	0.998	0.002	1.016	0.314	-	-	-	-
CYP1A1	rs2606345	A>C	0.690	0.310	0.653	0.347	1.302	0.254	1.182	0.887-1.576	0.846	0.634-1.128
CYP1A1	rs28399430	C>G	0.998	0.002	0.998	0.002	0.000	0.995	1.009	0.063-16.19	0.991	0.062-15.891
CYP1A1	rs34260157.1	A>G	1.000	0.000	0.988	0.012	5.053	0.025	-	-	-	-
CYP1A1	rs34260157.2	G>C	1.000	0.000	0.991	0.009	4.019	0.045	-	-	-	-
CYP1A1	rs35035798	T>C	1.000	0.000	0.998	0.002	1.001	0.317	-	-	-	-

CYP1A1	rs36121583	A>C	1.000	0.000	0.894	0.106	48.357	<0.0001	-	-	-	-
CYP1A1	rs41279188	T>G	0.995	0.005	0.988	0.012	1.296	0.255	2.517	0.486-13.047	0.397	0.077-2.059
CYP1A1	rs45442501	A>G	0.995	0.005	0.991	0.009	0.671	0.413	2.009	0.366-11.028	0.498	0.091-2.732
CYP1A1	rs45500996	A>G	1.000	0.000	0.991	0.009	4.000	0.045	-	-	-	-
CYP1A1	rs45528935	T>C	0.995	0.005	0.988	0.012	1.296	0.255	2.517	0.486-13.047	0.397	0.077-2.059
CYP1A1	rs4646422	T>C	0.938	0.063	0.960	0.040	2.156	0.142	0.630	0.338-1.173	1.588	0.852-2.959
CYP1A1	rs4986881.2	T>G	1.000	0.000	0.993	0.007	3.111	0.078	-	-	-	-
CYP1A1	rs4986884	A>G	1.000	0.000	0.993	0.007	2.997	0.083	-	-	-	-
CYP1A1	rs56240201.2	G>C	1.000	0.000	0.998	0.002	1.030	0.310	-	-	-	-
CYP1A1	rs56313657.1	A>C	1.000	0.000	0.993	0.007	3.067	0.080	-	-	-	-
CYP1A1	rs56313657.2	T>C	1.000	0.000	0.993	0.007	3.024	0.082	-	-	-	-
CYP1A1	rs61747605	A>G	0.995	0.005	0.991	0.009	0.671	0.413	2.009	0.366-11.028	0.498	0.091-2.732
CYP1A1	rs72547509	A>T	1.000	0.000	0.988	0.012	5.100	0.024	-	-	-	-
CYP1A1	rs72547510	I>D	0.995	0.005	0.872	0.128	53.162	<0.0001	31.729	7.675-131.174	0.032	0.008-0.13
CYP2B6	rs180791497	G>C	1.000	0.000	0.998	0.002	1.030	0.310	-	-	-	-
CYP2B6	rs149403002	T>G	1.000	0.000	0.898	0.102	46.373	<0.0001	-	-	-	-
CYP2B6	rs138652715	A>G	0.995	0.005	0.993	0.007	0.211	0.646	1.518	0.252-9.128	0.659	0.11-3.963
CYP2B6	rs192903015	T>C	0.995	0.005	0.902	0.098	38.911	<0.0001	23.503	5.652-97.737	0.043	0.01-0.177
CYP2B6	rs373489637	T>G	1.000	0.000	0.898	0.102	46.361	<0.0001	-	-	-	-
CYP2B6	rs28399491	A>G	0.986	0.014	0.984	0.016	0.073	0.786	1.164	0.388-3.492	0.859	0.286-2.577
CYP2B6	rs8192718	A>G	0.981	0.019	0.959	0.041	3.918	0.048	2.293	0.986-5.332	0.436	0.188-1.014
CYP2B6	rs111688353	A>G	0.993	0.007	0.991	0.009	0.154	0.695	1.349	0.3-6.064	0.741	0.165-3.332
CYP2B6	rs117398851	A>G	0.988	0.012	0.979	0.021	1.180	0.277	1.826	0.607-5.492	0.548	0.182-1.648
CYP2B6	rs12721656	A>G	0.998	0.002	1.000	0.000	0.997	0.318	-	-	-	-
CYP2B6	rs35449271	T>C	0.979	0.021	0.988	0.012	1.162	0.281	0.550	0.183-1.656	1.817	0.604-5.467
CYP2B6	rs7246465	T>C	0.673	0.327	0.691	0.309	0.339	0.561	0.918	0.688-1.225	1.089	0.816-1.454
CYP2B6	rs7260329	A>G	0.586	0.414	0.594	0.406	0.061	0.805	0.966	0.733-1.273	1.035	0.786-1.364
CYP2B6	rs1042389	T>C	0.757	0.243	0.750	0.250	0.056	0.813	1.039	0.755-1.43	0.962	0.699-1.324
CYP2B6	rs117323987	T>G	0.995	0.005	0.897	0.103	41.348	<0.0001	24.880	5.991-103.316	0.040	0.01-0.167
CYP2B6	rs12721646.1	C>G	1.000	0.000	0.995	0.005	2.033	0.154	-	-	-	-
CYP2B6	rs12721646.2	T>C	1.000	0.000	0.993	0.007	3.024	0.082	-	-	-	-
CYP2B6	rs12721649	A>G	1.000	0.000	0.913	0.087	39.235	<0.0001	-	-	-	-
CYP2B6	rs12721655	A>G	1.000	0.000	0.913	0.087	39.042	<0.0001	-	-	-	-
CYP2B6	rs138264188	T>C	0.995	0.005	0.911	0.089	34.508	<0.0001	21.087	5.049-88.078	0.047	0.011-0.198
CYP2B6	rs145884402	T>G	0.995	0.005	0.998	0.002	0.285	0.594	0.526	0.047-5.818	1.903	0.172-21.065
CYP2B6	rs186335453	T>G	0.990	0.010	0.894	0.106	35.048	<0.0001	12.202	4.322-34.451	0.082	0.029-0.231
CYP2B6	rs200238771	A>T	0.995	0.005	0.991	0.009	0.671	0.413	2.009	0.366-11.028	0.498	0.091-2.732
CYP2B6	rs2099361	A>C	0.281	0.719	0.274	0.726	0.061	0.806	1.038	0.77-1.4	0.963	0.714-1.299
CYP2B6	rs2279341	G>C	0.951	0.049	0.974	0.026	2.915	0.088	0.529	0.252-1.111	1.891	0.9-3.971
CYP2B6	rs2279342	T>A	0.850	0.150	0.835	0.165	0.350	0.554	1.118	0.772-1.62	0.894	0.617-1.295
CYP2B6	rs2279344	A>G	0.679	0.321	0.678	0.322	0.001	0.979	1.004	0.754-1.336	0.996	0.748-1.326
CYP2B6	rs2279345	T>C	0.680	0.320	0.601	0.399	5.583	0.018	1.409	1.06-1.873	0.710	0.534-0.944
CYP2B6	rs28399499	T>C	1.000	0.000	0.995	0.005	2.014	0.156	-	-	-	-

CYP2B6	rs33980385	A>G	1.000	0.000	0.995	0.005	2.343	0.126	-	-	-	-
CYP2B6	rs34646544	A>G	0.995	0.005	0.893	0.107	42.928	<0.0001	25.755	6.21-106.81	0.039	0.009-0.161
CYP2B6	rs34698757	G>C	1.000	0.000	0.993	0.007	2.983	0.084	-	-	-	-
CYP2B6	rs34826503	T>C	0.995	0.005	0.892	0.108	43.799	<0.0001	26.233	6.33-108.717	0.038	0.009-0.158
CYP2B6	rs35010098	A>C	0.995	0.005	0.991	0.009	0.671	0.413	2.009	0.366-11.028	0.498	0.091-2.732
CYP2B6	rs35266616	T>C	0.995	0.005	0.868	0.132	54.967	<0.0001	32.802	7.938-135.553	0.030	0.007-0.126
CYP2B6	rs35303484	A>G	0.995	0.005	0.995	0.005	0.000	1.000	1.000	0.14-7.132	1.000	0.14-7.132
CYP2B6	rs35303484.1	T>A	0.995	0.005	0.991	0.009	0.700	0.403	2.038	0.371-11.185	0.491	0.089-2.694
CYP2B6	rs35468935	T>C	0.995	0.005	0.993	0.007	0.201	0.654	1.503	0.25-9.043	0.665	0.111-4
CYP2B6	rs35622401	C>G	1.000	0.000	0.995	0.005	2.005	0.157	-	-	-	-
CYP2B6	rs35979566	A>T	1.000	0.000	0.993	0.007	3.082	0.079	-	-	-	-
CYP2B6	rs36056539	T>C	0.995	0.005	0.890	0.110	44.677	<0.0001	26.722	6.452-110.676	0.037	0.009-0.155
CYP2B6	rs36060847	A>G	0.995	0.005	0.898	0.102	40.059	<0.0001	24.166	5.815-100.433	0.041	0.01-0.172
CYP2B6	rs36118214	A>G	1.000	0.000	0.899	0.101	44.333	<0.0001	-	-	-	-
CYP2B6	rs3745274	T>G	0.775	0.225	0.697	0.303	6.327	0.012	1.495	1.092-2.047	0.669	0.488-0.916
CYP2B6	rs3786547	T>C	0.765	0.235	0.728	0.273	1.547	0.214	1.219	0.892-1.666	0.820	0.6-1.121
CYP2B6	rs45459594.1	C>G	0.998	0.002	0.993	0.007	1.043	0.307	3.071	0.318-29.645	0.326	0.034-3.143
CYP2B6	rs45459594.2	T>C	0.998	0.002	0.995	0.005	0.339	0.560	2.014	0.182-22.294	0.497	0.045-5.496
CYP2B6	rs45466193	T>G	1.000	0.000	0.893	0.107	49.054	<0.0001	-	-	-	-
CYP2B6	rs45482602	A>C	0.991	0.009	0.993	0.007	0.144	0.704	0.748	0.166-3.363	1.336	0.297-6.007
CYP2B6	rs4803417	A>C	0.669	0.331	0.653	0.347	0.228	0.633	1.073	0.804-1.43	0.932	0.699-1.243
CYP2B6	rs4803418	C>G	0.572	0.428	0.574	0.426	0.005	0.945	0.991	0.756-1.297	1.010	0.771-1.322
CYP2B6	rs4803419	T>C	0.664	0.336	0.637	0.363	0.707	0.400	1.130	0.85-1.502	0.885	0.666-1.176
CYP2B6	rs6508964	A>G	0.671	0.329	0.654	0.346	0.251	0.616	1.076	0.807-1.436	0.929	0.697-1.239
CYP2B6	rs707265	A>G	0.735	0.265	0.675	0.325	3.505	0.061	1.333	0.986-1.801	0.750	0.555-1.014
CYP2B6	rs71337576	A>G	0.323	0.677	0.364	0.636	1.606	0.205	0.831	0.624-1.107	1.204	0.904-1.603
CYP2B6	rs8101756	T>C	0.760	0.240	0.746	0.254	0.223	0.637	1.078	0.79-1.471	0.928	0.68-1.266
CYP2B6	rs8192709	T>C	0.970	0.030	0.970	0.030	0.000	0.990	0.995	0.456-2.173	1.005	0.46-2.194
CYP2B6	rs8192719	T>C	0.782	0.218	0.732	0.268	2.921	0.087	1.314	0.96-1.797	0.761	0.556-1.041
CYP2C19	rs1564657013	A>G	0.995	0.005	0.896	0.104	41.363	<0.0001	24.895	5.995-103.383	0.040	0.01-0.167
CYP2C19	rs375781227	A>G	0.995	0.005	0.991	0.009	0.662	0.416	2.000	0.364-10.977	0.500	0.091-2.744
CYP2C19	rs1564660997	T>C	0.998	0.002	0.895	0.105	44.886	<0.0001	50.610	6.943-368.915	0.020	0.003-0.144
CYP2C19	rs141417293	A>C	0.993	0.007	0.896	0.104	39.112	<0.0001	16.705	5.15-54.189	0.060	0.018-0.194
CYP2C19	rs150790215	A>G	0.995	0.005	0.993	0.007	0.211	0.646	1.518	0.252-9.129	0.659	0.11-3.963
CYP2C19	rs190777341	C>G	0.970	0.030	0.967	0.033	0.054	0.816	1.095	0.509-2.359	0.913	0.424-1.966
CYP2C19	rs575923433	T>C	0.995	0.005	0.989	0.011	1.268	0.260	2.494	0.481-12.926	0.401	0.077-2.078
CYP2C19	rs72552267	A>G	0.995	0.005	0.993	0.007	0.211	0.646	1.518	0.252-9.128	0.659	0.11-3.963
CYP2C19	rs4494250	A>G	0.710	0.290	0.671	0.329	1.466	0.226	1.199	0.893-1.61	0.834	0.621-1.119
CYP2C19	rs11592737	A>G	0.857	0.143	0.913	0.087	6.605	0.010	0.568	0.368-0.878	1.759	1.139-2.718
CYP2C19	rs116992754	T>G	0.995	0.005	0.886	0.114	46.129	<0.0001	27.613	6.666-114.388	0.036	0.009-0.15
CYP2C19	rs140554854	T>G	0.998	0.002	0.888	0.112	48.355	<0.0001	54.601	7.497-397.682	0.018	0.003-0.133
CYP2C19	rs75105257	T>C	0.998	0.002	0.993	0.007	1.014	0.314	3.028	0.314-29.225	0.330	0.034-3.188
CYP2C19	rs11188092	A>C	0.859	0.141	0.919	0.081	7.536	0.006	0.541	0.348-0.843	1.847	1.186-2.877

CYP2C19	rs11528090	T>G	0.712	0.288	0.689	0.311	0.531	0.466	1.114	0.833-1.492	0.897	0.67-1.201
CYP2C19	rs118203756	C>G	1.000	0.000	0.995	0.005	1.995	0.158	-	-	-	-
CYP2C19	rs118203757	A>G	0.995	0.005	0.989	0.011	1.282	0.257	2.506	0.484-12.986	0.399	0.077-2.068
CYP2C19	rs118203759	C>G	1.000	0.000	0.998	0.002	1.020	0.313	-	-	-	-
CYP2C19	rs12268020	T>C	0.855	0.145	0.835	0.165	0.645	0.422	1.164	0.803-1.688	0.859	0.592-1.245
CYP2C19	rs12571421	A>G	0.827	0.173	0.834	0.166	0.073	0.786	0.952	0.667-1.359	1.050	0.736-1.5
CYP2C19	rs12768009	A>G	0.788	0.212	0.792	0.208	0.025	0.873	0.974	0.701-1.353	1.027	0.739-1.427
CYP2C19	rs12769205	A>G	0.826	0.174	0.816	0.184	0.144	0.704	1.071	0.753-1.523	0.934	0.657-1.328
CYP2C19	rs138142612	A>G	0.995	0.005	0.894	0.106	42.693	<0.0001	25.686	6.185-106.679	0.039	0.009-0.162
CYP2C19	rs140278421	G>C	1.000	0.000	0.993	0.007	3.039	0.081	-	-	-	-
CYP2C19	rs144036596	A>G	0.993	0.007	0.991	0.009	0.158	0.691	1.355	0.302-6.092	0.738	0.164-3.316
CYP2C19	rs145119820	A>G	0.995	0.005	0.893	0.107	43.139	<0.0001	25.875	6.239-107.305	0.039	0.009-0.16
CYP2C19	rs145328984	T>C	0.995	0.005	0.895	0.105	42.027	<0.0001	25.247	6.084-104.767	0.040	0.01-0.164
CYP2C19	rs17878459.1	A>G	1.000	0.000	0.995	0.005	2.014	0.156	-	-	-	-
CYP2C19	rs17878459.2	C>G	0.988	0.012	0.991	0.009	0.099	0.754	0.809	0.216-3.035	1.235	0.329-4.632
CYP2C19	rs17878649	A>G	0.961	0.039	0.954	0.046	0.254	0.614	1.185	0.612-2.294	0.844	0.436-1.634
CYP2C19	rs17879685	T>C	0.995	0.005	0.892	0.108	43.613	<0.0001	26.199	6.313-108.735	0.038	0.009-0.158
CYP2C19	rs17884832	T>G	0.922	0.078	0.826	0.174	17.751	<0.0001	2.475	1.607-3.811	0.404	0.262-0.622
CYP2C19	rs17886522	A>C	0.965	0.035	0.960	0.040	0.111	0.739	1.128	0.556-2.289	0.887	0.437-1.799
CYP2C19	rs185136199	T>C	0.995	0.005	0.920	0.080	30.202	<0.0001	18.709	4.46-78.487	0.053	0.013-0.224
CYP2C19	rs200003088	A>G	0.995	0.005	0.887	0.113	45.850	<0.0001	27.429	6.622-113.615	0.036	0.009-0.151
CYP2C19	rs200936950	A>G	0.995	0.005	0.988	0.012	1.282	0.257	2.506	0.484-12.987	0.399	0.077-2.068
CYP2C19	rs201132803	A>T	1.000	0.000	0.995	0.005	2.043	0.153	-	-	-	-
CYP2C19	rs28399505	T>C	1.000	0.000	0.998	0.002	0.992	0.319	-	-	-	-
CYP2C19	rs28399513	A>T	0.823	0.177	0.819	0.181	0.016	0.899	1.023	0.722-1.45	0.978	0.69-1.385
CYP2C19	rs28399514	A>G	0.995	0.005	0.991	0.009	0.681	0.409	2.019	0.368-11.08	0.495	0.09-2.719
CYP2C19	rs375283723	T>C	1.000	0.000	0.892	0.108	49.246	<0.0001	-	-	-	-
CYP2C19	rs377184510	A>G	1.000	0.000	0.998	0.002	0.997	0.318	-	-	-	-
CYP2C19	rs41291556	T>C	1.000	0.000	0.901	0.099	44.595	<0.0001	-	-	-	-
CYP2C19	rs4244285.1	A>G	0.827	0.173	0.826	0.174	0.004	0.950	1.011	0.711-1.438	0.989	0.695-1.406
CYP2C19	rs4388808	A>G	0.708	0.292	0.710	0.290	0.003	0.954	0.991	0.736-1.336	1.009	0.749-1.359
CYP2C19	rs4417205	G>C	0.826	0.174	0.822	0.178	0.023	0.878	1.028	0.723-1.461	0.973	0.685-1.383
CYP2C19	rs4917612	C>G	0.739	0.261	0.714	0.286	0.715	0.398	1.139	0.842-1.54	0.878	0.649-1.187
CYP2C19	rs4917623	T>C	0.419	0.581	0.383	0.617	1.147	0.284	1.161	0.884-1.525	0.861	0.656-1.132
CYP2C19	rs4986893	A>G	0.961	0.039	0.863	0.137	25.449	<0.0001	3.861	2.211-6.742	0.259	0.148-0.452
CYP2C19	rs545642100	G>C	1.000	0.000	0.993	0.007	3.039	0.081	-	-	-	-
CYP2C19	rs550527959	A>T	1.000	0.000	0.998	0.002	1.006	0.316	-	-	-	-
CYP2C19	rs55640102.1	A>C	1.000	0.000	0.995	0.005	2.023	0.155	-	-	-	-
CYP2C19	rs55640102.2	T>A	1.000	0.000	0.993	0.007	3.068	0.080	-	-	-	-
CYP2C19	rs55752064	T>C	1.000	0.000	0.912	0.088	39.965	<0.0001	-	-	-	-
CYP2C19	rs55948420	A>G	0.995	0.005	0.895	0.105	42.027	<0.0001	25.247	6.084-104.767	0.040	0.01-0.164
CYP2C19	rs559628884	A>C	0.993	0.007	0.899	0.101	37.542	<0.0001	16.130	4.964-52.412	0.062	0.019-0.201
CYP2C19	rs56337013	T>C	0.995	0.005	0.931	0.069	24.965	<0.0001	15.891	3.753-67.277	0.063	0.015-0.266

CYP2C19	rs5787121	D>I	0.995	0.005	0.993	0.007	0.206	0.650	1.510	0.251-9.085	0.662	0.11-3.982
CYP2C19	rs6413438	T>C	0.995	0.005	0.988	0.012	1.310	0.252	2.530	0.488-13.11	0.395	0.076-2.049
CYP2C19	rs72552267	A>G	0.995	0.005	0.993	0.007	0.211	0.646	1.518	0.252-9.128	0.659	0.11-3.963
CYP2C19	rs72558185	D>I	1.000	0.000	0.995	0.005	1.995	0.158	-	-	-	-
CYP2C19	rs72558186	T>A	1.000	0.000	0.995	0.005	2.043	0.153	-	-	-	-
CYP2C19	rs770829708.1	A>G	1.000	0.000	0.995	0.005	2.034	0.154	-	-	-	-
CYP2C19	rs778258371	A>G	0.995	0.005	0.991	0.009	0.690	0.406	2.028	0.37-11.133	0.493	0.09-2.706
CYP2C19	rs7916649	A>G	0.604	0.396	0.584	0.416	0.337	0.562	1.084	0.825-1.426	0.922	0.701-1.213
CYP2D6	rs75467367	C>G	1.000	0.000	0.995	0.005	2.023	0.155	-	-	-	-
CYP2D6	rs180847475	G>C	1.000	0.000	0.995	0.005	2.014	0.156	-	-	-	-
CYP2D6	rs186133763	C>G	1.000	0.000	0.991	0.009	4.037	0.045	-	-	-	-
CYP2D6	rs28371706	A>G	1.000	0.000	0.988	0.012	5.300	0.021	-	-	-	-
CYP2D6	rs140513104	A>G	0.998	0.002	0.893	0.107	46.006	<0.0001	51.870	7.119-377.921	0.019	0.003-0.14
CYP2D6	rs59421388	T>C	1.000	0.000	0.905	0.095	43.154	<0.0001	-	-	-	-
CYP2D6	rs61736512	T>C	0.998	0.002	0.896	0.104	44.663	<0.0001	50.349	6.907-367.003	0.020	0.003-0.145
CYP2D6	rs28371732	T>C	0.993	0.007	0.890	0.110	40.918	<0.0001	17.424	5.375-56.488	0.057	0.018-0.186
CYP2D6	rs5030866	T>C	0.995	0.005	0.984	0.016	2.784	0.095	3.524	0.728-17.063	0.284	0.059-1.374
CYP2D6	rs1058172	T>C	0.917	0.083	0.891	0.109	1.621	0.203	1.346	0.851-2.128	0.743	0.47-1.175
CYP2D6	rs1135824	T>C	1.000	0.000	0.998	0.002	1.035	0.309	-	-	-	-
CYP2D6	rs1135832.1	A>C	1.000	0.000	0.995	0.005	2.052	0.152	-	-	-	-
CYP2D6	rs1135832.2	G>C	1.000	0.000	0.998	0.002	1.011	0.315	-	-	-	-
CYP2D6	rs1135833	G>C	1.000	0.000	1.000	0.000	-	-	-	-	-	-
CYP2D6	rs1135835	T>C	1.000	0.000	0.897	0.103	46.806	<0.0001	-	-	-	-
CYP2D6	rs1135838	A>C	0.998	0.002	0.899	0.101	42.882	<0.0001	48.389	6.632-353.083	0.021	0.003-0.151
CYP2D6	rs118203758	T>C	0.995	0.005	0.988	0.012	1.324	0.250	2.541	0.49-13.17	0.394	0.076-2.039
CYP2D6	rs138100349	A>G	1.000	0.000	0.890	0.110	50.424	<0.0001	-	-	-	-
CYP2D6	rs138417770	T>C	0.995	0.005	0.995	0.005	0.000	0.985	1.019	0.143-7.266	0.981	0.138-7
CYP2D6	rs139779104	T>C	0.998	0.002	0.984	0.016	4.486	0.034	7.033	0.862-57.406	0.142	0.017-1.161
CYP2D6	rs142302759	T>G	1.000	0.000	0.897	0.103	46.608	<0.0001	-	-	-	-
CYP2D6	rs146540061	A>G	1.000	0.000	0.902	0.098	44.569	<0.0001	-	-	-	-
CYP2D6	rs146838345	T>C	0.995	0.005	0.991	0.009	0.700	0.403	2.038	0.371-11.185	0.491	0.089-2.694
CYP2D6	rs147943410	C>G	1.000	0.000	0.991	0.009	4.037	0.045	-	-	-	-
CYP2D6	rs147960066	A>G	0.998	0.002	0.988	0.012	2.742	0.098	5.119	0.595-44	0.195	0.023-1.679
CYP2D6	rs148250267	T>C	1.000	0.000	0.900	0.100	45.686	<0.0001	-	-	-	-
CYP2D6	rs148382141	T>C	1.000	0.000	0.894	0.106	48.357	<0.0001	-	-	-	-
CYP2D6	rs149157808	T>C	1.000	0.000	0.895	0.105	47.928	<0.0001	-	-	-	-
CYP2D6	rs149686350	A>G	0.998	0.002	0.894	0.106	45.552	<0.0001	51.335	7.046-374.015	0.019	0.003-0.142
CYP2D6	rs150445731	A>G	0.998	0.002	0.889	0.111	48.088	<0.0001	54.269	7.451-395.251	0.018	0.003-0.134
CYP2D6	rs188062577	T>C	0.995	0.005	0.894	0.106	42.916	<0.0001	25.741	6.207-106.747	0.039	0.009-0.161
CYP2D6	rs199722016	T>C	1.000	0.000	0.998	0.002	0.997	0.318	-	-	-	-
CYP2D6	rs199849357	A>G	1.000	0.000	0.991	0.009	4.000	0.045	-	-	-	-
CYP2D6	rs201297021	T>C	0.998	0.002	0.986	0.014	3.794	0.051	6.306	0.756-52.605	0.159	0.019-1.323
CYP2D6	rs201377835	C>G	1.000	0.000	0.993	0.007	3.010	0.083	-	-	-	-

CYP2D6	rs267608279	D>I	1.000	0.000	0.993	0.007	3.039	0.081	-	-	-	-
CYP2D6	rs267608285	T>G	1.000	0.000	0.889	0.111	50.128	<0.0001	-	-	-	-
CYP2D6	rs267608289	T>C	0.986	0.014	0.993	0.007	1.011	0.315	0.496	0.123-1.998	2.014	0.5-8.106
CYP2D6	rs267608290	T>G	1.000	0.000	0.900	0.100	45.263	<0.0001	-	-	-	-
CYP2D6	rs267608291	T>C	0.998	0.002	0.993	0.007	1.005	0.316	3.014	0.312-29.091	0.332	0.034-3.202
CYP2D6	rs267608295.2	G>C	1.000	0.000	0.995	0.005	1.995	0.158	-	-	-	-
CYP2D6	rs267608295.3	T>G	1.000	0.000	0.995	0.005	2.005	0.157	-	-	-	-
CYP2D6	rs267608297	A>G	1.000	0.000	0.981	0.019	8.352	0.004	-	-	-	-
CYP2D6	rs267608298	A>G	0.998	0.002	0.993	0.007	1.092	0.296	3.145	0.326-30.36	0.318	0.033-3.069
CYP2D6	rs267608302	T>G	1.000	0.000	0.995	0.005	1.986	0.159	-	-	-	-
CYP2D6	rs267608305	T>C	1.000	0.000	0.898	0.102	46.373	<0.0001	-	-	-	-
CYP2D6	rs267608308	T>C	0.995	0.005	0.895	0.105	42.040	<0.0001	25.261	6.087-104.83	0.040	0.01-0.164
CYP2D6	rs267608309	A>G	1.000	0.000	0.991	0.009	3.981	0.046	-	-	-	-
CYP2D6	rs267608310	A>G	0.998	0.002	0.991	0.009	1.825	0.177	4.047	0.45-36.357	0.247	0.028-2.22
CYP2D6	rs267608313	A>G	1.000	0.000	0.984	0.016	6.992	0.008	-	-	-	-
CYP2D6	rs267608316	A>C	1.000	0.000	0.896	0.104	47.259	<0.0001	-	-	-	-
CYP2D6	rs267608322	A>C	0.995	0.005	0.998	0.002	0.330	0.566	0.501	0.045-5.548	1.995	0.18-22.088
CYP2D6	rs28371706.1	A>G	0.998	0.002	0.992	0.008	1.253	0.263	3.384	0.35-32.673	0.296	0.031-2.853
CYP2D6	rs28371717	A>C	0.993	0.007	0.982	0.018	2.256	0.133	2.673	0.704-10.143	0.374	0.099-1.42
CYP2D6	rs28371725	T>C	0.936	0.064	0.856	0.144	14.500	<0.0001	2.466	1.531-3.97	0.406	0.252-0.653
CYP2D6	rs28371729	T>G	0.998	0.002	0.895	0.105	44.900	<0.0001	50.640	6.947-369.137	0.020	0.003-0.144
CYP2D6	rs28371733	T>C	1.000	0.000	0.893	0.107	48.819	<0.0001	-	-	-	-
CYP2D6	rs28371735	A>G	1.000	0.000	0.874	0.126	57.783	<0.0001	-	-	-	-
CYP2D6	rs28371736.1	A>G	1.000	0.000	0.998	0.002	1.015	0.314	-	-	-	-
CYP2D6	rs28371736.2	G>C	1.000	0.000	0.998	0.002	1.011	0.315	-	-	-	-
CYP2D6	rs35742686	I>D	0.998	0.002	0.893	0.107	45.791	<0.0001	51.630	7.086-376.179	0.019	0.003-0.141
CYP2D6	rs367543000	A>G	0.998	0.002	0.989	0.011	2.667	0.102	5.023	0.584-43.176	0.199	0.023-1.711
CYP2D6	rs368389952	T>C	1.000	0.000	0.986	0.014	5.931	0.015	-	-	-	-
CYP2D6	rs370580423	A>G	0.998	0.002	0.986	0.014	3.600	0.058	6.070	0.728-50.634	0.165	0.02-1.374
CYP2D6	rs371181941	A>G	1.000	0.000	0.894	0.106	48.587	<0.0001	-	-	-	-
CYP2D6	rs371793722	A>G	1.000	0.000	0.922	0.078	35.224	<0.0001	-	-	-	-
CYP2D6	rs377591409	A>G	0.998	0.002	0.991	0.009	1.824	0.177	4.047	0.45-36.355	0.247	0.028-2.22
CYP2D6	rs5030862	T>C	0.995	0.005	0.993	0.007	0.206	0.650	1.510	0.251-9.085	0.662	0.11-3.982
CYP2D6	rs5030867	T>G	0.998	0.002	0.991	0.009	1.797	0.180	4.009	0.446-36.018	0.249	0.028-2.241
CYP2D6	rs536049502	T>A	1.000	0.000	0.993	0.007	3.096	0.078	-	-	-	-
CYP2D6	rs544790460	A>C	1.000	0.000	0.897	0.103	46.806	<0.0001	-	-	-	-
CYP2D6	rs554540676	T>G	1.000	0.000	0.895	0.105	47.485	<0.0001	-	-	-	-
CYP2D6	rs554875652	A>C	0.998	0.002	0.984	0.016	4.514	0.034	7.065	0.866-57.672	0.142	0.017-1.155
CYP2D6	rs567606867	T>C	1.000	0.000	0.889	0.111	50.824	<0.0001	-	-	-	-
CYP2D6	rs569926140	A>C	1.000	0.000	0.894	0.106	48.175	<0.0001	-	-	-	-
CYP2D6	rs67497403	A>G	0.998	0.002	0.993	0.007	1.005	0.316	3.014	0.312-29.09	0.332	0.034-3.202
CYP2D6	rs72549346	I>D	1.000	0.000	0.995	0.005	2.005	0.157	-	-	-	-
CYP2D6	rs72549349	C>G	1.000	0.000	0.995	0.005	2.062	0.151	-	-	-	-

CYP2D6	rs72549351	I>D	1.000	0.000	0.896	0.104	47.259	<0.0001	-	-	-	-
CYP2D6	rs72549354	D>I	1.000	0.000	0.998	0.002	0.997	0.318	-	-	-	-
CYP2D6	rs72549358	T>C	0.998	0.002	0.889	0.111	47.915	<0.0001	54.125	7.428-394.395	0.018	0.003-0.135
CYP2D6	rs730882170	D>I	0.998	0.002	0.988	0.012	2.742	0.098	5.119	0.595-44	0.195	0.023-1.679
CYP2D6	rs74478221	T>C	0.995	0.005	0.983	0.017	2.832	0.092	3.559	0.735-17.235	0.281	0.058-1.36
CYP2D6	rs745746329	T>C	0.995	0.005	0.887	0.113	45.850	<0.0001	27.429	6.622-113.615	0.036	0.009-0.151
CYP2D6	rs747998333	A>G	1.000	0.000	0.985	0.015	6.367	0.012	-	-	-	-
CYP2D6	rs74802369	T>A	1.000	0.000	0.993	0.007	3.067	0.080	-	-	-	-
CYP2D6	rs748712690	T>C	1.000	0.000	0.920	0.080	36.010	<0.0001	-	-	-	-
CYP2D6	rs74962936	A>G	0.998	0.002	0.989	0.011	2.667	0.102	5.023	0.584-43.176	0.199	0.023-1.711
CYP2D6	rs750996195	T>C	0.998	0.002	0.897	0.103	43.988	<0.0001	49.615	6.803-361.835	0.020	0.003-0.147
CYP2D6	rs758320086	I>D	1.000	0.000	0.998	0.002	0.997	0.318	-	-	-	-
CYP2D6	rs76187628	A>G	0.993	0.007	0.907	0.093	31.106	<0.0001	13.728	4.202-44.85	0.073	0.022-0.238
CYP2D6	rs765776661	I>D	0.998	0.002	0.993	0.007	1.005	0.316	3.014	0.312-29.09	0.332	0.034-3.202
CYP2D6	rs766507177	T>G	1.000	0.000	0.901	0.099	44.797	<0.0001	-	-	-	-
CYP2D6	rs77312092	T>C	1.000	0.000	0.991	0.009	4.075	0.044	-	-	-	-
CYP2D6	rs773790593	A>G	1.000	0.000	0.895	0.105	47.699	<0.0001	-	-	-	-
CYP2D6	rs774943042	A>C	1.000	0.000	0.897	0.103	46.806	<0.0001	-	-	-	-
CYP2D6	rs78482768	C>G	1.000	0.000	0.991	0.009	4.000	0.045	-	-	-	-
CYP2D6	rs79292917	T>C	0.998	0.002	0.881	0.119	51.171	<0.0001	57.728	7.939-419.761	0.017	0.002-0.126
CYP2D6	rs79392742	T>G	0.995	0.005	0.995	0.005	0.000	0.996	1.005	0.141-7.165	0.995	0.14-7.099
CYP2D6	rs79738337	A>G	0.998	0.002	0.993	0.007	1.033	0.309	3.057	0.317-29.504	0.327	0.034-3.158
CYP2D6	rs5030655	I>D	1.000	0.000	0.889	0.111	50.076	<0.0001	-	-	-	-
CYP2D6	rs747955910	I>D	0.998	0.002	0.991	0.009	1.810	0.178	4.028	0.448-36.185	0.248	0.028-2.23
GCLC	rs114704032	T>C	1.000	0.000	0.998	0.002	0.992	0.319	-	-	-	-
GCLC	rs12524550	T>C	0.988	0.012	0.961	0.039	6.378	0.012	3.458	1.245-9.606	0.289	0.104-0.803
GCLC	rs12524652	T>C	0.986	0.014	0.889	0.111	34.032	<0.0001	8.727	3.689-20.644	0.115	0.048-0.271
GCLC	rs17193216	A>G	0.984	0.016	0.977	0.023	0.540	0.462	1.439	0.543-3.815	0.695	0.262-1.843
GCLC	rs17884118	T>C	0.988	0.012	0.993	0.007	0.486	0.486	0.603	0.143-2.538	1.659	0.394-6.985
GCLC	rs2397147	T>C	0.778	0.222	0.838	0.162	4.927	0.026	0.675	0.477-0.956	1.481	1.046-2.096
GCLC	rs41271285	T>C	0.998	0.002	0.995	0.005	0.330	0.566	1.995	0.18-22.088	0.501	0.045-5.548
GCLC	rs41271289	T>C	0.993	0.007	0.895	0.105	39.325	<0.0001	16.792	5.177-54.472	0.060	0.018-0.193
GCLC	rs77516417	A>C	0.995	0.005	0.889	0.111	44.973	<0.0001	26.947	6.501-111.69	0.037	0.009-0.154
GCLC	rs12525474	T>C	0.836	0.164	0.798	0.202	2.003	0.157	1.286	0.907-1.824	0.777	0.548-1.102
GCLC	rs1555906	A>G	0.685	0.315	0.640	0.360	1.906	0.167	1.222	0.919-1.624	0.819	0.616-1.088
GCLC	rs17883718	A>G	1.000	0.000	0.892	0.108	49.468	<0.0001	-	-	-	-
GCLC	rs17884046	A>C	0.924	0.076	0.928	0.072	0.058	0.810	0.939	0.564-1.563	1.065	0.64-1.772
GCLC	rs3799694	T>C	0.708	0.292	0.638	0.362	4.758	0.029	1.375	1.032-1.831	0.727	0.546-0.969
GCLC	rs3799700	T>C	0.749	0.251	0.780	0.220	1.135	0.287	0.841	0.612-1.156	1.188	0.865-1.633
GCLC	rs4715407	A>G	0.804	0.196	0.720	0.280	8.015	0.005	1.598	1.153-2.213	0.626	0.452-0.867
GCLC	rs524553	T>C	0.871	0.129	0.791	0.209	9.835	0.002	1.780	1.238-2.561	0.562	0.39-0.808
GCLC	rs547222	T>C	0.816	0.184	0.753	0.247	4.873	0.027	1.455	1.042-2.032	0.687	0.492-0.96
GCLC	rs648595	T>G	0.505	0.495	0.500	0.500	0.018	0.893	1.019	0.778-1.333	0.982	0.75-1.285

GCLC	rs680403	T>C	0.535	0.465	0.526	0.474	0.068	0.794	1.037	0.788-1.366	0.964	0.732-1.269
GCLC	rs761142	A>C	0.689	0.311	0.669	0.331	0.388	0.534	1.096	0.822-1.46	0.913	0.685-1.217
GCLC	rs9474588	A>G	0.675	0.325	0.644	0.356	0.920	0.337	1.148	0.866-1.521	0.871	0.657-1.155
GCLC	rs17883901	C>T	0.912	0.088	0.889	0.111	1.227	0.268	1.286	0.823-2.009	0.777	0.498-1.215
GCLM	rs17376966	T>C	0.993	0.007	0.995	0.005	0.201	0.654	0.665	0.111-4	1.503	0.25-9.043
GCLM	rs3789453	T>C	0.778	0.222	0.791	0.209	0.224	0.636	0.924	0.667-1.28	1.082	0.781-1.498
GCLM	rs41303970	C>T	0.881	0.119	0.873	0.127	0.130	0.718	1.077	0.72-1.611	0.929	0.621-1.389
GPX4	rs117193629	T>C	0.942	0.058	0.972	0.028	4.463	0.035	0.477	0.236-0.961	2.099	1.04-4.234
GPX4	rs73507255	A>G	1.000	0.000	0.898	0.102	46.361	<0.0001	-	-	-	-
GPX4	rs76201145	A>G	0.995	0.005	0.989	0.011	1.282	0.257	2.506	0.484-12.986	0.399	0.077-2.068
GPX4	rs713041	C>T	0.528	0.472	0.550	0.450	0.437	0.508	0.914	0.701-1.192	1.094	0.839-1.426
GSTM1	rs185525957	A>G	0.995	0.005	0.986	0.014	2.000	0.157	3.014	0.605-15.016	0.332	0.067-1.653
GSTM1	rs2239892	A>G	0.940	0.060	0.855	0.145	16.788	<0.0001	2.652	1.64-4.287	0.377	0.233-0.61
GSTM1	Null	I>D	0.500	0.500	0.533	0.467	0.956	0.328	0.875	0.669-1.144	1.143	0.874-1.494
GSTP1	rs1138272	T>C	0.926	0.074	0.969	0.031	8.097	0.004	0.395	0.205-0.764	2.529	1.308-4.888
GSTP1	rs11553890	T>C	1.000	0.000	0.989	0.011	4.983	0.026	-	-	-	-
GSTP1	rs11553892.1	A>C	1.000	0.000	0.993	0.007	2.997	0.083	-	-	-	-
GSTP1	rs11553892.2	G>C	1.000	0.000	0.986	0.014	6.042	0.014	-	-	-	-
GSTP1	rs11553893	T>G	0.995	0.005	0.893	0.107	42.718	<0.0001	25.635	6.181-106.314	0.039	0.009-0.162
GSTP1	rs12796085	T>C	0.998	0.002	0.907	0.093	38.647	<0.0001	43.709	5.976-319.678	0.023	0.003-0.167
GSTP1	rs1871042	T>C	0.723	0.277	0.797	0.203	6.394	0.011	0.665	0.484-0.913	1.504	1.095-2.065
GSTP1	rs41462048	A>G	0.998	0.002	0.993	0.007	0.995	0.318	3.000	0.311-28.955	0.333	0.035-3.217
GSTP1	rs4147581	G>C	0.512	0.488	0.597	0.403	6.391	0.011	0.707	0.54-0.925	1.415	1.081-1.853
GSTP1	rs45543438	A>G	0.995	0.005	0.991	0.009	0.671	0.413	2.009	0.366-11.028	0.498	0.091-2.732
GSTP1	rs4986949	T>G	0.988	0.012	0.877	0.123	42.232	<0.0001	11.927	4.706-30.23	0.084	0.033-0.212
GSTP1	rs749174	A>G	0.723	0.277	0.759	0.241	1.357	0.244	0.830	0.607-1.135	1.204	0.881-1.647
GSTP1	rs762803	A>C	0.672	0.328	0.728	0.272	3.059	0.080	0.765	0.566-1.033	1.307	0.968-1.765
GSTP1	rs8191439	A>G	0.972	0.028	0.977	0.023	0.150	0.698	0.845	0.361-1.978	1.183	0.506-2.768
GSTP1	rs8191441	A>G	0.991	0.009	0.988	0.012	0.112	0.738	1.253	0.334-4.698	0.798	0.213-2.993
GSTP1	rs8191444	G>C	1.000	0.000	0.995	0.005	2.014	0.156	-	-	-	-
GSTP1	rs1695	A>G	0.695	0.305	0.772	0.228	6.532	0.011	0.674	0.498-0.913	1.484	1.095-2.009
GSTT1	Null	I>D	0.590	0.410	0.567	0.433	0.471	0.493	1.100	0.838-1.443	0.909	0.693-1.193
NAT2	rs76846598	T>C	0.988	0.012	0.989	0.011	0.000	0.982	0.986	0.283-3.431	1.014	0.291-3.528
NAT2	rs146789770	A>C	0.995	0.005	0.993	0.007	0.192	0.661	1.490	0.248-8.959	0.671	0.112-4.038
NAT2	rs150339859	T>C	0.995	0.005	0.897	0.103	41.132	<0.0001	24.750	5.96-102.776	0.040	0.01-0.168
NAT2	rs45594437	A>G	0.988	0.012	0.993	0.007	0.477	0.490	0.606	0.144-2.55	1.651	0.392-6.953
NAT2	rs1041983	T>C	0.647	0.353	0.550	0.450	8.505	0.004	1.504	1.143-1.98	0.665	0.505-0.875
NAT2	rs1208	A>G	0.750	0.250	0.815	0.185	5.224	0.022	0.680	0.488-0.947	1.471	1.055-2.05
NAT2	rs12720065	G>C	1.000	0.000	0.991	0.009	4.037	0.045	-	-	-	-
NAT2	rs138707146	T>C	0.993	0.007	0.896	0.104	38.276	<0.0001	16.413	5.055-53.293	0.061	0.019-0.198
NAT2	rs139351995	A>C	1.000	0.000	0.998	0.002	1.001	0.317	-	-	-	-
NAT2	rs144176822	T>G	0.995	0.005	0.988	0.012	1.296	0.255	2.518	0.486-13.048	0.397	0.077-2.059
NAT2	rs149283608	A>T	1.000	0.000	0.993	0.007	3.097	0.078	-	-	-	-

NAT2	rs1799929	T>C	0.774	0.226	0.767	0.233	0.066	0.797	1.044	0.753-1.447	0.958	0.691-1.328
NAT2	rs1799930	A>G	0.749	0.251	0.680	0.320	4.887	0.027	1.404	1.039-1.899	0.712	0.527-0.963
NAT2	rs1799931	A>G	0.898	0.102	0.795	0.205	17.629	<0.0001	2.277	1.54-3.365	0.439	0.297-0.649
NAT2	rs1801279	A>G	0.995	0.005	0.988	0.012	1.296	0.255	2.517	0.486-13.047	0.397	0.077-2.059
NAT2	rs1801280	T>C	0.753	0.247	0.786	0.214	1.273	0.259	0.833	0.607-1.144	1.200	0.874-1.647
NAT2	rs1805158	T>C	0.995	0.005	0.989	0.011	1.282	0.257	2.506	0.484-12.986	0.399	0.077-2.068
NAT2	rs45477599	T>A	1.000	0.000	0.993	0.007	3.024	0.082	-	-	-	-
NAT2	rs45518335	T>C	0.995	0.005	0.993	0.007	0.215	0.642	1.525	0.254-9.172	0.656	0.109-3.945
NAT2	rs45607939	T>A	0.998	0.002	0.991	0.009	1.824	0.177	4.047	0.45-36.355	0.247	0.028-2.22
NAT2	rs45618543	T>G	0.993	0.007	0.895	0.105	39.325	<0.0001	16.792	5.177-54.472	0.060	0.018-0.193
NAT2	rs4986996	A>G	0.998	0.002	0.959	0.041	15.410	<0.0001	18.644	2.47-140.747	0.054	0.007-0.405
NAT2	rs4986997	A>T	1.000	0.000	0.993	0.007	2.997	0.083	-	-	-	-
NAT2	rs55700793	A>G	1.000	0.000	0.998	0.002	1.001	0.317	-	-	-	-
NAT2	rs56011192	T>C	0.993	0.007	0.988	0.012	0.495	0.482	1.667	0.396-7.018	0.600	0.142-2.526
NAT2	rs56054745.1	A>C	0.995	0.005	0.991	0.009	0.690	0.406	2.028	0.37-11.132	0.493	0.09-2.706
NAT2	rs56054745.2	A>G	1.000	0.000	0.993	0.007	3.067	0.080	-	-	-	-
NAT2	rs56387565	T>C	1.000	0.000	0.995	0.005	1.986	0.159	-	-	-	-
NAT2	rs56393504	A>G	0.993	0.007	0.894	0.106	39.568	<0.0001	16.900	5.209-54.826	0.059	0.018-0.192
NAT2	rs72466459	T>C	0.995	0.005	0.884	0.116	47.062	<0.0001	28.257	6.811-117.225	0.035	0.009-0.147
NAT2	rs72466460	T>C	1.000	0.000	0.880	0.120	55.014	<0.0001	-	-	-	-
NAT2	rs72466461	A>G	1.000	0.000	0.995	0.005	1.995	0.158	-	-	-	-
NAT2	rs72554615	T>C	1.000	0.000	0.896	0.104	47.248	<0.0001	-	-	-	-
NAT2	rs72554616	A>C	1.000	0.000	0.998	0.002	0.997	0.318	-	-	-	-
NAT2	rs72554617	A>G	0.988	0.012	0.883	0.117	39.783	<0.0001	11.296	4.458-28.622	0.089	0.035-0.224
NAT2	rs79050330	T>C	0.995	0.005	0.881	0.119	48.671	<0.0001	29.153	7.036-120.787	0.034	0.008-0.142
NFE2	rs10506328	A>C	0.840	0.160	0.848	0.152	0.101	0.751	0.942	0.652-1.362	1.062	0.734-1.535
NFE2L1	rs147114188	A>G	0.972	0.028	0.866	0.134	33.119	<0.0001	5.454	2.884-10.312	0.183	0.097-0.347
NFE2L1	rs150840650	T>C	0.995	0.005	0.991	0.009	0.671	0.413	2.009	0.366-11.028	0.498	0.091-2.732
NFE2L1	rs2023885	A>G	0.856	0.144	0.771	0.229	10.132	0.001	1.768	1.241-2.517	0.566	0.397-0.806
NFE2L1	rs2229367	G>C	0.998	0.002	0.993	0.007	1.072	0.300	3.116	0.323-30.074	0.321	0.033-3.098
NFE2L2	rs10930781	A>G	0.748	0.252	0.768	0.232	0.473	0.492	0.893	0.647-1.233	1.120	0.811-1.546
NFE2L2	rs11686945	T>C	0.753	0.247	0.806	0.194	3.472	0.062	0.735	0.532-1.017	1.360	0.984-1.881
NFE2L2	rs13001694	A>G	0.800	0.200	0.836	0.164	1.981	0.159	0.780	0.552-1.103	1.282	0.907-1.812
NFE2L2	rs199673454	A>T	1.000	0.000	0.998	0.002	1.025	0.311	-	-	-	-
NFE2L2	rs2001350	T>C	0.765	0.235	0.794	0.206	1.038	0.308	0.844	0.609-1.17	1.185	0.855-1.643
NFE2L2	rs34468415	A>G	0.811	0.189	0.849	0.151	2.183	0.140	0.764	0.535-1.092	1.308	0.915-1.869
NFE2L3	rs11770841	T>G	0.588	0.412	0.591	0.409	0.009	0.925	0.987	0.752-1.295	1.013	0.772-1.329
NFE2L3	rs2237329	T>C	0.866	0.134	0.780	0.220	10.971	0.001	1.829	1.276-2.624	0.547	0.381-0.784
NFE2L3	rs79693596	A>G	0.977	0.023	0.961	0.039	1.804	0.179	1.722	0.772-3.839	0.581	0.26-1.295
NFE2L3	rs12113404	A>G	0.769	0.231	0.670	0.330	10.407	0.001	1.637	1.212-2.211	0.611	0.452-0.825
NQO1	rs117363962	T>C	0.995	0.005	0.903	0.097	37.829	<0.0001	22.909	5.504-95.347	0.044	0.01-0.182
NQO1	rs143567674	T>C	0.995	0.005	0.883	0.117	47.589	<0.0001	28.484	6.875-118.001	0.035	0.008-0.145
NQO1	rs45467396	A>G	0.991	0.009	0.995	0.005	0.681	0.409	0.495	0.09-2.719	2.019	0.368-11.08

NQO1	rs76921462	T>C	0.910	0.090	0.857	0.143	5.756	0.016	1.684	1.096-2.587	0.594	0.387-0.912
NQO1	rs77097817	T>C	0.988	0.012	0.984	0.016	0.357	0.550	1.420	0.447-4.51	0.704	0.222-2.236
NQO1	rs10517	A>G	0.780	0.220	0.778	0.222	0.005	0.943	1.012	0.731-1.402	0.988	0.713-1.369
NQO1	rs1131341	G>C	1.000	0.000	0.993	0.007	3.067	0.080	-	-	-	-
NQO1	rs1131341.1	A>G	0.979	0.021	0.967	0.033	1.241	0.265	1.613	0.69-3.767	0.620	0.265-1.448
NQO1	rs114238154	A>G	1.000	0.000	0.995	0.005	2.014	0.156	-	-	-	-
NQO1	rs1800566	A>G	0.623	0.377	0.543	0.457	5.693	0.017	1.394	1.061-1.833	0.717	0.546-0.943
NQO1	rs201787127	T>C	0.995	0.005	0.938	0.062	21.973	<0.0001	14.182	3.344-60.139	0.071	0.017-0.299
NQO1	rs2917670	T>C	0.625	0.375	0.624	0.376	0.000	0.986	1.003	0.76-1.322	0.998	0.757-1.315
NQO1	rs2917677	T>C	0.757	0.243	0.697	0.303	3.907	0.048	1.356	1.002-1.835	0.738	0.545-0.998
NQO1	rs34447156	C>G	0.998	0.002	0.991	0.009	1.824	0.177	4.047	0.45-36.355	0.247	0.028-2.22
PON1	rs3917592	T>C	0.995	0.005	0.995	0.005	0.001	0.978	0.972	0.136-6.934	1.029	0.144-7.336
PON1	rs78307684	T>G	0.995	0.005	0.894	0.106	41.013	<0.0001	24.685	5.948-102.45	0.041	0.01-0.168
PON1	rs854570	A>C	0.532	0.468	0.459	0.541	4.570	0.033	1.343	1.025-1.759	0.745	0.568-0.976
PON1	rs13306698	T>C	0.944	0.056	0.849	0.151	21.438	<0.0001	3.032	1.862-4.94	0.330	0.202-0.537
PON1	rs199851417	A>G	0.995	0.005	0.910	0.090	35.251	<0.0001	21.487	5.149-89.66	0.047	0.011-0.194
PON1	rs2057681	A>G	0.600	0.400	0.567	0.433	0.953	0.329	1.144	0.873-1.499	0.874	0.667-1.145
PON1	rs2299260	T>C	0.780	0.220	0.815	0.185	1.663	0.197	0.803	0.575-1.121	1.245	0.892-1.739
PON1	rs3917477	A>G	0.928	0.072	0.923	0.077	0.078	0.780	1.075	0.646-1.79	0.930	0.559-1.548
PON1	rs3917503	T>C	0.574	0.426	0.516	0.484	2.903	0.088	1.263	0.965-1.652	0.792	0.605-1.036
PON1	rs3917550	A>G	0.922	0.078	0.893	0.107	2.108	0.147	1.409	0.885-2.243	0.710	0.446-1.129
PON1	rs3917594	T>C	0.995	0.005	0.989	0.011	1.268	0.260	2.494	0.481-12.926	0.401	0.077-2.078
PON1	rs662	T>C	0.600	0.400	0.562	0.438	1.217	0.270	1.166	0.888-1.531	0.858	0.653-1.127
PON1	rs854552	T>C	0.705	0.295	0.667	0.333	1.442	0.230	1.193	0.894-1.591	0.838	0.629-1.118
PON1	rs854555	A>C	0.414	0.586	0.462	0.538	1.917	0.166	0.825	0.629-1.083	1.211	0.923-1.59
PON1	rs854562	T>C	0.843	0.157	0.814	0.186	1.159	0.282	1.222	0.848-1.761	0.818	0.568-1.179
PON1	rs854565	A>G	0.592	0.408	0.599	0.401	0.041	0.839	0.972	0.739-1.279	1.029	0.782-1.354
PON1	rs854568	A>G	0.295	0.705	0.291	0.709	0.019	0.891	1.021	0.761-1.368	0.980	0.731-1.313
PON2	rs375489124	T>C	0.995	0.005	0.947	0.053	17.424	<0.0001	11.984	2.748-52.251	0.083	0.019-0.364
PON2	rs144782675	T>C	0.998	0.002	1.000	0.000	1.006	0.316	-	-	-	-
PON2	rs12534274	A>G	0.628	0.372	0.531	0.469	8.155	0.004	1.490	1.133-1.96	0.671	0.51-0.883
PON2	rs17717505	A>C	1.000	0.000	0.865	0.135	62.315	<0.0001	-	-	-	-
PON2	rs10261470	A>G	0.936	0.064	0.943	0.057	0.159	0.690	0.889	0.497-1.588	1.125	0.63-2.011
PON2	rs11981433	T>C	0.829	0.171	0.842	0.158	0.244	0.622	0.913	0.636-1.311	1.095	0.763-1.572
PON2	rs201301042	T>C	0.995	0.005	0.903	0.097	38.411	<0.0001	23.244	5.585-96.742	0.043	0.01-0.179
PON2	rs2299267	A>G	0.717	0.283	0.664	0.336	2.703	0.100	1.278	0.954-1.713	0.782	0.584-1.049
PON2	rs7493	G>C	0.699	0.301	0.691	0.309	0.071	0.789	1.040	0.778-1.39	0.961	0.719-1.285
PON2	rs77619496	A>G	0.995	0.005	0.897	0.103	41.132	<0.0001	24.750	5.96-102.776	0.040	0.01-0.168
PON2	rs7785039	T>C	0.811	0.189	0.832	0.168	0.580	0.446	0.869	0.606-1.247	1.150	0.802-1.649
PON3	rs377433561	T>C	0.993	0.007	0.988	0.012	0.524	0.469	1.690	0.401-7.117	0.592	0.141-2.491
PON3	rs138268669	A>G	0.973	0.027	0.859	0.141	35.441	<0.0001	6.037	3.119-11.683	0.166	0.086-0.321
PON3	rs17883823	T>C	1.000	0.000	0.900	0.100	45.673	<0.0001	-	-	-	-
PON3	rs368482537	A>G	1.000	0.000	0.894	0.106	48.345	<0.0001	-	-	-	-

PON3	rs763839908	T>C	1.000	0.000	0.896	0.104	47.248	<0.0001	-	-	-	-
PON3	rs17883750	A>G	0.942	0.058	0.952	0.048	0.407	0.523	0.824	0.454-1.495	1.214	0.669-2.203
PON3	rs17885558	A>G	0.938	0.063	0.865	0.135	12.392	<0.0001	2.345	1.443-3.81	0.426	0.262-0.693
PON3	rs10953143	T>C	0.433	0.567	0.408	0.592	0.545	0.460	1.109	0.843-1.457	0.902	0.686-1.186
PON3	rs139856535	T>C	0.995	0.005	0.993	0.007	0.206	0.650	1.511	0.251-9.086	0.662	0.11-3.982
PON3	rs141350740	A>G	0.970	0.030	0.981	0.019	1.173	0.279	0.614	0.252-1.497	1.629	0.668-3.971
PON3	rs142620825	A>G	1.000	0.000	0.998	0.002	0.997	0.318	-	-	-	-
PON3	rs17883013	T>G	1.000	0.000	0.900	0.100	45.686	<0.0001	-	-	-	-
SOD1	rs114905802	T>C	0.995	0.005	0.993	0.007	0.211	0.646	1.518	0.252-9.129	0.659	0.11-3.963
SOD1	rs138002121	G>C	1.000	0.000	0.991	0.009	4.076	0.044	-	-	0.000	-
SOD1	rs183603801	C>G	1.000	0.000	0.998	0.002	1.001	0.317	-	-	-	-
SOD1	rs76067554	G>C	0.982	0.018	0.988	0.012	0.593	0.441	0.645	0.209-1.987	1.551	0.503-4.781
SOD1	rs1041740	T>C	0.655	0.345	0.591	0.409	3.747	0.053	1.314	0.996-1.732	0.761	0.577-1.004
SOD1	rs121912438	G>C	1.000	0.000	0.993	0.007	3.039	0.081	-	-	-	-
SOD1	rs121912441	T>C	1.000	0.000	0.998	0.002	0.997	0.318	-	-	-	-
SOD1	rs121912442	T>C	0.995	0.005	0.991	0.009	0.662	0.416	2.000	0.364-10.977	0.500	0.091-2.744
SOD1	rs121912443	A>G	1.000	0.000	0.907	0.093	42.244	<0.0001	-	-	-	-
SOD1	rs121912451	A>G	0.995	0.005	0.890	0.110	44.092	<0.0001	26.462	6.38-109.756	0.038	0.009-0.157
SOD1	rs121912455	A>G	0.995	0.005	0.993	0.007	0.201	0.654	1.503	0.25-9.043	0.665	0.111-4
SOD1	rs4816407	A>G	0.726	0.274	0.755	0.245	0.930	0.335	0.860	0.633-1.168	1.163	0.856-1.58
SOD1	rs80265967	A>C	1.000	0.000	0.896	0.104	47.259	<0.0001	-	-	-	-
SOD2	rs113886498	A>G	0.995	0.005	0.913	0.088	31.875	<0.0001	19.658	4.695-82.303	0.051	0.012-0.213
SOD2	rs117466922	A>G	0.984	0.016	0.988	0.012	0.292	0.589	0.728	0.229-2.312	1.374	0.433-4.363
SOD2	rs12190141	A>G	0.977	0.023	0.988	0.012	1.673	0.196	0.496	0.168-1.465	2.014	0.683-5.943
SOD2	rs12204454	T>C	0.663	0.337	0.629	0.371	1.076	0.300	1.159	0.877-1.532	0.863	0.653-1.14
SOD2	rs13212047	A>C	0.995	0.005	0.885	0.115	45.086	<0.0001	27.061	6.527-112.186	0.037	0.009-0.153
SOD2	rs140181088	A>G	0.963	0.037	0.963	0.037	0.001	0.979	0.990	0.489-2.007	1.010	0.498-2.046
SOD2	rs4516970	A>G	0.972	0.028	0.969	0.031	0.069	0.793	1.112	0.502-2.466	0.899	0.405-1.993
SOD2	rs5746081	A>G	0.972	0.028	0.857	0.143	36.457	<0.0001	5.878	3.104-11.133	0.170	0.09-0.322
SOD2	rs75374960	T>C	0.988	0.012	0.984	0.016	0.357	0.550	1.420	0.447-4.509	0.704	0.222-2.237
SOD2	rs80065806	A>C	0.946	0.054	0.967	0.033	2.158	0.142	0.604	0.307-1.191	1.655	0.84-3.263
SOD2	rs143582231	A>G	0.995	0.005	0.901	0.099	39.130	<0.0001	23.639	5.684-98.308	0.042	0.01-0.176
SOD2	rs2758348	T>C	0.829	0.171	0.779	0.221	3.167	0.075	1.375	0.967-1.954	0.727	0.512-1.034
SOD2	rs2842974	T>C	0.685	0.315	0.671	0.329	0.208	0.649	1.069	0.802-1.425	0.935	0.702-1.247
SOD2	rs4709364	T>C	0.683	0.317	0.668	0.332	0.197	0.657	1.068	0.798-1.431	0.936	0.699-1.253
SOD2	rs4880	A>G	0.687	0.313	0.657	0.343	0.844	0.358	1.144	0.859-1.524	0.874	0.656-1.165
SOD3	rs17880193	T>C	0.995	0.005	0.991	0.009	0.671	0.413	2.009	0.366-11.028	0.498	0.091-2.732
SOD3	rs17878863	A>G	0.958	0.042	0.945	0.055	0.778	0.378	1.332	0.703-2.521	0.751	0.397-1.422
SOD3	rs17880362	T>C	0.970	0.030	0.868	0.133	30.036	<0.0001	4.946	2.653-9.223	0.202	0.108-0.377
SOD3	rs800444	A>G	0.995	0.005	0.946	0.054	17.255	<0.0001	11.660	2.716-50.068	0.086	0.02-0.368
SOD3	rs368892083	A>G	0.995	0.005	0.880	0.120	49.179	<0.0001	29.455	7.109-122.039	0.034	0.008-0.141
SOD3	rs1799895	C>G	0.967	0.033	0.962	0.038	0.215	0.643	1.189	0.573-2.467	0.841	0.405-1.746
SOD3	rs8192290	T>C	0.947	0.053	0.876	0.124	13.542	<0.0001	2.527	1.521-4.198	0.396	0.238-0.657

SOD3	rs2536512	A>G	0.588	0.412	0.620	0.380	0.904	0.342	0.876	0.666-1.151	1.142	0.868-1.502
SOD3	rs2855262	T>C	0.588	0.412	0.609	0.391	0.378	0.539	0.917	0.697-1.208	1.090	0.828-1.435
SRXN1	rs6053666	T>C	0.576	0.424	0.565	0.435	0.121	0.728	1.049	0.8-1.377	0.953	0.726-1.25
SRXN1	rs6107696	A>G	0.366	0.634	0.329	0.671	1.293	0.256	1.179	0.888-1.565	0.848	0.639-1.127
SRXN1	rs7268200	A>G	0.788	0.212	0.686	0.314	11.465	0.001	1.699	1.248-2.313	0.589	0.432-0.801
TXNRD1	rs550114104	T>C	1.000	0.000	0.998	0.002	0.992	0.319	-	-	-	-
TXNRD1	rs117567389	T>C	0.867	0.133	0.804	0.196	5.953	0.015	1.597	1.094-2.33	0.626	0.429-0.914
TXNRD1	rs34337366	T>G	1.000	0.000	0.991	0.009	4.019	0.045	-	-	0.000	-
TXNRD1	rs35232644	T>C	0.988	0.012	0.988	0.012	0.000	0.994	0.995	0.286-3.463	1.005	0.289-3.496
TXNRD1	rs4411337	T>C	0.705	0.295	0.663	0.337	1.678	0.195	1.213	0.905-1.626	0.824	0.615-1.105
TXNRD1	rs61937916	A>G	0.975	0.025	0.970	0.030	0.171	0.679	1.187	0.526-2.681	0.842	0.373-1.901
TXNRD1	rs73392601	A>C	0.968	0.032	0.972	0.028	0.159	0.690	0.853	0.39-1.866	1.172	0.536-2.564
TXNRD1	rs74990047	A>G	0.998	0.002	0.880	0.120	51.414	<0.0001	58.227	7.997-423.98	0.017	0.002-0.125
TXNRD1	rs75300553	A>G	0.998	0.002	1.000	0.000	0.992	0.319	-	-	-	-
TXNRD1	rs77176894	T>C	0.979	0.021	0.984	0.016	0.274	0.601	0.767	0.283-2.078	1.304	0.481-3.533
TXNRD1	rs4077561	T>C	0.426	0.574	0.493	0.507	3.881	0.049	0.763	0.583-0.999	1.310	1.001-1.715
TXNRD1	rs4445711	A>G	0.242	0.758	0.275	0.725	1.201	0.273	0.843	0.621-1.144	1.186	0.874-1.611
TXNRD1	rs7301631	T>C	0.291	0.709	0.363	0.637	5.049	0.025	0.718	0.537-0.959	1.393	1.043-1.861
TXNRD1	rs7975161	T>C	0.898	0.102	0.804	0.196	14.933	<0.0001	2.146	1.448-3.181	0.466	0.314-0.69
UCP3	rs117552079	A>G	0.975	0.025	0.968	0.032	0.357	0.550	1.276	0.573-2.842	0.784	0.352-1.746
UCP3	rs1685325	T>C	0.521	0.479	0.495	0.505	0.562	0.454	1.108	0.847-1.449	0.902	0.69-1.18
UCP3	rs1726743	T>C	0.961	0.039	0.963	0.037	0.036	0.849	0.934	0.466-1.874	1.070	0.534-2.146
UCP3	rs76833611	T>C	0.988	0.012	0.979	0.021	1.180	0.277	1.826	0.607-5.492	0.548	0.182-1.648
UCP3	rs11235971	T>C	0.714	0.286	0.649	0.351	4.077	0.043	1.351	1.008-1.809	0.740	0.553-0.992
UCP3	rs138705669	T>C	0.995	0.005	0.890	0.110	44.526	<0.0001	26.710	6.44-110.779	0.037	0.009-0.155
UCP3	rs17848368	A>G	0.995	0.005	0.993	0.007	0.201	0.654	1.503	0.25-9.043	0.665	0.111-4.001
UCP3	rs183714776	T>C	0.995	0.005	0.984	0.016	2.760	0.097	3.508	0.725-16.985	0.285	0.059-1.38
UCP3	rs199679366	T>C	0.995	0.005	0.991	0.009	0.690	0.406	2.028	0.37-11.132	0.493	0.09-2.706
UCP3	rs201993988	T>C	0.995	0.005	0.886	0.114	46.738	<0.0001	27.926	6.746-115.608	0.036	0.009-0.148
UCP3	rs2734827	A>G	0.701	0.299	0.686	0.314	0.239	0.625	1.075	0.805-1.436	0.930	0.696-1.243
UCP3	rs45476292	T>C	0.995	0.005	0.989	0.011	1.282	0.257	2.506	0.484-12.986	0.399	0.077-2.068
UCP3	rs76629964	A>G	0.995	0.005	0.906	0.094	37.093	<0.0001	22.500	5.402-93.72	0.044	0.011-0.185
Note: *A – Ancestral allele, *B – Derived allele												

Table S4. Association between individual SNPs and pesticide exposure

Gene	rs ID	Alleles	χ2	*p*-valume	OR for AA genotype	95% CI	OR for AB genotype	95% CI	OR for BB genotype	95% CI	Hardy–Weinberg equilibrium χ2 >> p value	
											Control	Case
AKR1B10	rs77337210	A>G	11.571	0.003	11.796	1.495-93.109	0.000	-	0.893	0.055-14.391	4.284//0.117	198//0

AKR1B10	rs1722883	T>C	7.141	0.028	1.159	0.771-1.742	0.659	0.445-0.974	1.609	0.962-2.693	1.07//0.586	4.939//0.085
AKR1C1	rs113645163	T>G	-	-	1.500	0.417-5.392	0.667	0.185-2.396	-	-	0.042//0.979	0.019//0.991
AKR1C1	rs117795769	A>G	45.266	<0.0001	28.261	6.745-118.416	0.019	0.003-0.137	0.476	0.043-5.293	0.129//0.937	93.108//0
AKR1C1	rs142200840	A>C	51.580	<0.0001	30.525	7.302-127.598	0.016	0.002-0.119	-	-	3.215//0.2	95.774//0
AKR1C1	rs2904799	A>G	7.268	0.026	1.556	1.046-2.314	0.812	0.544-1.211	0.577	0.314-1.059	0.84//0.657	0.748//0.688
AKR1C1	rs2904802	T>C	6.026	0.049	1.619	1.096-2.39	0.621	0.418-0.924	0.854	0.304-2.398	3.399//0.183	0.07//0.965
AKR1C1	rs7076886	T>C	0.163	0.922	0.929	0.618-1.398	1.085	0.709-1.661	0.990	0.385-2.545	1.588//0.452	0.84//0.657
APOE	rs150375400	A>G	-	-	1.327	0.293-6.001	0.754	0.167-3.407	-	-	0.019//0.991	0.011//0.995
APOE	rs11083750	A>C	-	-	-	-	0.000	-	-	-	0.011//0.995	-
APOE	rs11083750.1	G>C	2.012	0.366	-	-	0.000	-	0.000	-	94.885//0	-
APOE	rs140808909	A>G	47.814	<0.0001	52.561	7.161-385.809	0.000	-	-	-	2.548//0.28	215//0
APOE	rs190853081	A>G	1.007	0.604	0.660	0.109-3.993	1.005	0.14-7.2	-	-	0.005//0.998	51.995//0
APOE	rs199768005	A>T	-	-	2.038	0.183-22.645	0.491	0.044-5.453	-	-	0.005//0.998	0.001//0.999
APOE	rs201672011	A>G	2.049	0.359	3.057	0.315-29.627	0.000	-	0.991	0.062-15.942	52.245//0	215//0
APOE	rs267606661	C>G	-	-	-	-	0.000	-	-	-	0.005//0.998	-
APOE	rs267606664	A>G	0.991	0.609	1.991	0.361-10.984	0.335	0.035-3.245	1.014	0.063-16.317	33.677//0	94.885//0
APOE	rs28931577	A>G	0.338	0.845	1.507	0.249-9.11	0.498	0.045-5.53	1.000	0.062-16.091	53.245//0	95.774//0
APOE	rs387906567	T>C	1.017	0.601	1.963	0.177-21.809	0.000	-	1.024	0.064-16.476	95.33//0	211//0
APOE	rs7412	T>C	3.429	0.180	0.607	0.32-1.15	1.807	0.928-3.517	0.491	0.044-5.452	6.381//0.041	0.01//0.995
APOE	rs7412_10AT	T>C	2.234	0.327	0.621	0.326-1.181	1.646	0.852-3.179	1.005	0.062-16.169	1.112//0.573	0.028//0.986
APOE	rs7412_15AT	T>C	-	-	0.678	0.342-1.347	1.474	0.743-2.927	-	-	0.29//0.865	0.625//0.732
APOE	rs769449	A>G	1.659	0.436	0.784	0.495-1.241	1.328	0.83-2.124	0.648	0.107-3.918	0.196//0.907	0.718//0.699
APOE	rs769455	T>C	2.000	0.368	3.014	0.311-29.21	0.000	-	1.005	0.062-16.168	52.745//0	214//0
CYP1A1	rs1799814	T>G	34.063	<0.0001	13.750	4.143-45.635	0.025	0.003-0.184	1.903	0.171-21.154	0.178//0.915	132.311//0
CYP1A1	rs143070677	C>G	-	-	4.195	0.465-37.849	0.238	0.026-2.151	-	-	0.02//0.99	0.001//0.999
CYP1A1	rs17861084	T>G	14.419	0.001	9.247	2.099-40.747	0.058	0.008-0.441	0.931	0.058-14.977	1.257//0.533	95.774//0
CYP1A1	rs17861094	A>G	-	-	-	-	0.000	-	-	-	0.001//0.999	-
CYP1A1	rs180744198	T>C	49.018	<0.0001	56.579	7.72-414.655	0.000	-	1.000	0.062-16.092	1.074//0.585	216//0
CYP1A1	rs201691396	A>C	48.244	<0.0001	-	-	0.000	-	0.000	-	0.874//0.646	-
CYP1A1	rs2278970.2	G>C	-	-	-	-	0.000	-	-	-	0.001//0.999	-

CYP1A1	rs2606345	A>C	7.591	0.022	1.518	1.031-2.235	0.589	0.4-0.866	1.390	0.723-2.67	5.776//0.056	1.047//0.592
CYP1A1	rs28399430	C>G	-	-	1.009	0.063-16.243	0.991	0.062-15.942	-	-	0.001//0.999	0.001//0.999
CYP1A1	rs34260157.1	A>G	3.044	0.218	-	-	0.000	-	0.000	-	136.792//0	-
CYP1A1	rs34260157.2	G>C	-	-	-	-	-	-	0.000	-	216//0	-
CYP1A1	rs35035798	T>C	-	-	-	-	0.000	-	-	-	0.001//0.999	-
CYP1A1	rs36121583	A>C	49.988	<0.0001	-	-	0.000	-	0.000	-	1.061//0.588	-
CYP1A1	rs41279188	T>G	3.023	0.221	4.056	0.45-36.59	0.000	-	1.000	0.062-16.091	33.517//0	217//0
CYP1A1	rs45442501	A>G	2.012	0.366	3.028	0.312-29.342	0.000	-	1.000	0.062-16.091	53.245//0	217//0
CYP1A1	rs45500996	A>G	3.007	0.222	-	-	0.000	-	0.000	-	53.245//0	-
CYP1A1	rs45528935	T>C	3.023	0.221	4.056	0.45-36.59	0.000	-	1.000	0.062-16.091	33.517//0	217//0
CYP1A1	rs4646422	T>C	7.053	0.029	0.536	0.276-1.039	2.176	1.09-4.342	0.000	-	8.712//0.013	0.96//0.619
CYP1A1	rs4986881.2	T>G	-	-	-	-	0.000	-	-	-	0.011//0.995	-
CYP1A1	rs4986884	A>G	2.000	0.368	-	-	0.000	-	0.000	-	95.774//0	-
CYP1A1	rs56240201.2	G>C	-	-	-	-	0.000	-	-	-	0.001//0.999	-
CYP1A1	rs56313657.1	A>C	-	-	-	-	0.000	-	-	-	0.011//0.995	-
CYP1A1	rs56313657.2	T>C	2.028	0.363	-	-	0.000	-	0.000	-	95.33//0	-
CYP1A1	rs61747605	A>G	2.012	0.366	3.028	0.312-29.342	0.000	-	1.000	0.062-16.091	53.245//0	217//0
CYP1A1	rs72547509	A>T	4.131	0.127	-	-	0.000	-	0.000	-	32.877//0	-
CYP1A1	rs72547510	I>D	61.197	<0.0001	72.474	9.907-530.168	0.000	-	0.935	0.058-15.052	2.144//0.342	217//0
CYP2B6	rs180791497	G>C	-	-	-	-	0.000	-	-	-	0.001//0.999	-
CYP2B6	rs149403002	T>G	-	-	-	-	0.000	-	-	-	2.794//0.247	-
CYP2B6	rs138652715	A>G	1.032	0.597	2.028	0.183-22.535	0.000	-	0.991	0.062-15.943	94.885//0	217//0
CYP2B6	rs192903015	T>C	48.006	<0.0001	52.744	7.186-387.122	0.000	-	-	-	2.534//0.282	217//0
CYP2B6	rs373489637	T>G	-	-	-	-	0.000	-	-	-	2.778//0.249	-
CYP2B6	rs28399491	A>G	0.109	0.947	1.200	0.361-3.992	0.800	0.212-3.02	1.005	0.062-16.165	16.374//0	22.778//0
CYP2B6	rs8192718	A>G	3.821	0.148	2.210	0.933-5.235	0.483	0.202-1.154	0.000	-	1.145//0.564	0.077//0.962
CYP2B6	rs111688353	A>G	-	-	1.352	0.299-6.115	0.739	0.164-3.344	-	-	0.019//0.991	0.011//0.995
CYP2B6	rs117398851	A>G	1.666	0.435	2.048	0.607-6.905	0.419	0.107-1.64	0.995	0.062-16.017	9.137//0.01	33.517//0
CYP2B6	rs12721656	A>G	-	-	0.000	-	-	-	-	-	-	0.001//0.999
CYP2B6	rs35449271	T>C	1.640	0.440	0.491	0.145-1.654	2.378	0.607-9.32	1.000	0.062-16.092	33.357//0	9.137//0.01

CYP2B6	rs7246465	T>C	3.473	0.176	1.044	0.713-1.528	0.788	0.537-1.156	1.714	0.895-3.284	1.603//0.449	1.351//0.509
CYP2B6	rs7260329	A>G	2.328	0.312	1.098	0.731-1.651	0.791	0.539-1.162	1.340	0.782-2.295	3.365//0.186	0.001//1
CYP2B6	rs1042389	T>C	2.605	0.272	1.156	0.781-1.71	0.788	0.529-1.173	1.636	0.631-4.245	4//0.135	0.021//0.99
CYP2B6	rs117323987	T>G	50.759	<0.0001	56.237	7.669-412.366	0.000	-	-	-	2.826//0.243	217//0
CYP2B6	rs12721646.1	C>G	-	-	-	-	0.000	-	-	-	0.005//0.998	-
CYP2B6	rs12721646.2	T>C	-	-	-	-	0.000	-	-	-	0.011//0.995	-
CYP2B6	rs12721649	A>G	40.034	<0.0001	-	-	0.000	-	0.000	-	0.245//0.885	-
CYP2B6	rs12721655	A>G	-	-	-	-	0.000	-	-	-	1.867//0.393	-
CYP2B6	rs138264188	T>C	43.276	<0.0001	46.737	6.348-344.106	0.000	-	-	-	1.982//0.371	217//0
CYP2B6	rs145884402	T>G	2.340	0.310	1.054	0.065-16.957	0.000	-	-	-	0.001//0.999	217//0
CYP2B6	rs186335453	T>G	41.059	<0.0001	17.799	5.398-58.691	0.039	0.009-0.162	0.908	0.056-14.617	0.748//0.688	50.745//0
CYP2B6	rs200238771	A>T	5.023	0.081	4.056	0.45-36.59	0.000	-	-	-	0.019//0.991	217//0
CYP2B6	rs2099361	A>C	4.479	0.106	1.880	0.906-3.904	0.732	0.496-1.079	1.127	0.771-1.646	1.787//0.409	2.657//0.265
CYP2B6	rs2279341	G>C	2.537	0.281	0.553	0.249-1.227	1.658	0.709-3.877	2.930	0.302-28.392	5.331//0.07	13.416//0.001
CYP2B6	rs2279342	T>A	1.740	0.419	1.173	0.777-1.772	0.833	0.551-1.259	-	-	8.169//0.017	4.176//0.124
CYP2B6	rs2279344	A>G	0.854	0.653	1.087	0.745-1.587	0.848	0.578-1.245	1.205	0.667-2.177	0.04//0.98	2.309//0.315
CYP2B6	rs2279345	T>C	17.283	<0.0001	2.106	1.408-3.149	0.455	0.307-0.674	1.198	0.653-2.195	9.721//0.008	2.185//0.335
CYP2B6	rs28399499	T>C	-	-	-	-	0.000	-	-	-	0.005//0.998	-
CYP2B6	rs33980385	A>G	-	-	-	-	0.000	-	-	-	0.005//0.997	-
CYP2B6	rs34646544	A>G	49.267	<0.0001	56.912	7.765-417.108	0.000	-	0.995	0.062-16.017	1.087//0.581	216//0
CYP2B6	rs34698757	G>C	-	-	-	-	0.000	-	-	-	0.011//0.995	-
CYP2B6	rs34826503	T>C	50.235	<0.0001	58.105	7.932-425.633	0.000	-	1.000	0.062-16.091	1.18//0.554	217//0
CYP2B6	rs35010098	A>C	5.023	0.081	4.056	0.45-36.59	0.000	-	-	-	0.019//0.991	217//0
CYP2B6	rs35266616	T>C	63.375	<0.0001	75.383	10.307-551.317	0.000	-	0.926	0.058-14.903	2.362//0.307	217//0
CYP2B6	rs35303484	A>G	-	-	1.000	0.14-7.164	1.000	0.14-7.164	-	-	0.005//0.998	0.005//0.998
CYP2B6	rs35303484.1	T>A	2.075	0.354	3.071	0.317-29.761	0.000	-	0.986	0.061-15.868	52.495//0	217//0
CYP2B6	rs35468935	T>C	1.005	0.605	2.009	0.181-22.325	0.000	-	1.000	0.062-16.091	95.774//0	217//0
CYP2B6	rs35622401	C>G	-	-	-	-	0.000	-	-	-	0.005//0.998	-
CYP2B6	rs35979566	A>T	-	-	-	-	0.000	-	-	-	0.011//0.995	-
CYP2B6	rs36056539	T>C	51.231	<0.0001	59.368	8.108-434.692	0.000	-	1.005	0.062-16.165	1.289//0.525	217//0

CYP2B6	rs36060847	A>G	46.084	<0.0001	52.935	7.211-388.585	0.000	-	0.986	0.061-15.867	0.802//0.67	214//0
CYP2B6	rs36118214	A>G	-	-	-	-	0.000	-	-	-	2.361//0.307	-
CYP2B6	rs3745274	T>G	8.406	0.015	1.721	1.161-2.553	0.622	0.417-0.929	0.708	0.323-1.554	0.867//0.648	0.216//0.898
CYP2B6	rs3786547	T>C	2.368	0.306	1.226	0.832-1.807	0.896	0.602-1.334	0.673	0.31-1.46	0.168//0.919	0//1
CYP2B6	rs45459594.1	C>G	-	-	3.086	0.318-29.908	0.324	0.033-3.14	-	-	0.011//0.995	0.001//0.999
CYP2B6	rs45459594.2	T>C	-	-	2.019	0.182-22.43	0.495	0.045-5.504	-	-	0.005//0.998	0.001//0.999
CYP2B6	rs45466193	T>G	50.749	<0.0001	-	-	0.000	-	0.000	-	1.1//0.577	-
CYP2B6	rs45482602	A>C	0.338	0.845	0.664	0.11-4.011	2.009	0.181-22.325	1.000	0.062-16.091	95.774//0	53.245//0
CYP2B6	rs4803417	A>C	7.020	0.030	1.356	0.919-2.001	0.618	0.418-0.913	1.528	0.83-2.813	2.375//0.305	3.785//0.151
CYP2B6	rs4803418	C>G	0.818	0.664	1.088	0.727-1.628	0.846	0.58-1.235	1.171	0.715-1.919	0.786//0.675	0.149//0.928
CYP2B6	rs4803419	T>C	2.340	0.310	1.326	0.874-2.012	0.741	0.49-1.122	1.934	0.174-21.491	61.966//0	45.699//0
CYP2B6	rs6508964	A>G	12.477	0.002	1.477	0.999-2.185	0.527	0.356-0.779	1.957	1.019-3.758	7.463//0.024	3.917//0.141
CYP2B6	rs707265	A>G	7.077	0.029	1.606	1.08-2.388	0.637	0.428-0.946	0.000	-	41.334//0	27.762//0
CYP2B6	rs71337576	A>G	5.310	0.070	1.080	0.603-1.935	0.664	0.45-0.981	1.454	0.985-2.147	0.546//0.761	2.834//0.242
CYP2B6	rs8101756	T>C	0.379	0.827	1.070	0.73-1.57	0.977	0.658-1.449	0.833	0.386-1.795	0.272//0.873	0.04//0.98
CYP2B6	rs8192709	T>C	0.000	1.000	0.995	0.437-2.267	1.005	0.426-2.37	1.005	0.062-16.168	3.493//0.174	3.47//0.176
CYP2B6	rs8192719	T>C	3.016	0.221	1.392	0.948-2.044	0.768	0.518-1.139	0.708	0.318-1.58	0.008//0.996	0.095//0.953
CYP2C19	rs1564657013	A>G	-	-	28.024	6.697-117.268	0.036	0.009-0.149	-	-	2.842//0.241	0.005//0.998
CYP2C19	rs375781227	A>G	2.000	0.368	3.014	0.311-29.206	0.000	-	1.005	0.062-16.166	53.245//0	216//0
CYP2C19	rs1564660997	T>C	46.335	<0.0001	55.579	7.58-407.516	0.019	0.003-0.136	0.000	-	0.972//0.615	0.001//0.999
CYP2C19	rs141417293	A>C	48.110	<0.0001	28.289	6.766-118.279	0.018	0.002-0.129	-	-	2.92//0.232	95.774//0
CYP2C19	rs150790215	A>G	1.033	0.597	2.028	0.183-22.538	0.000	-	0.991	0.062-15.942	94.441//0	216//0
CYP2C19	rs190777341	C>G	0.099	0.952	1.105	0.492-2.48	0.899	0.388-2.084	0.986	0.061-15.868	2.755//0.252	3.516//0.172
CYP2C19	rs575923433	T>C	2.995	0.224	4.019	0.446-36.251	0.000	-	1.009	0.063-16.241	33.677//0	216//0
CYP2C19	rs72552267	A>G	1.032	0.597	2.028	0.183-22.535	0.000	-	0.991	0.062-15.943	94.885//0	217//0
CYP2C19	rs4494250	A>G	13.099	0.001	1.639	1.111-2.416	0.502	0.339-0.745	1.721	0.868-3.412	5.889//0.053	4.887//0.087
CYP2C19	rs11592737	A>G	6.450	0.040	0.576	0.357-0.928	1.562	0.953-2.562	3.433	0.705-16.722	0.151//0.928	2.032//0.362
CYP2C19	rs116992754	T>G	-	-	31.556	7.558-131.754	0.032	0.008-0.132	-	-	3.496//0.174	0.005//0.998
CYP2C19	rs140554854	T>G	-	-	62.377	8.516-456.87	0.016	0.002-0.117	-	-	3.354//0.187	0.001//0.999
CYP2C19	rs75105257	T>C	-	-	3.042	0.314-29.48	0.329	0.034-3.185	-	-	0.011//0.995	0.001//0.999

CYP2C19	rs11188092	A>C	7.147	0.028	0.546	0.336-0.887	1.650	0.997-2.73	3.450	0.708-16.803	0.326//0.849	2.325//0.313
CYP2C19	rs11528090	T>G	1.718	0.423	1.239	0.849-1.808	0.776	0.528-1.139	1.100	0.573-2.109	0.282//0.868	0.986//0.611
CYP2C19	rs118203756	C>G	-	-	-	-	0.000	-	-	-	0.005//0.998	-
CYP2C19	rs118203757	A>G	3.007	0.222	4.037	0.448-36.419	0.000	-	1.005	0.062-16.165	33.677//0	217//0
CYP2C19	rs118203759	C>G	-	-	-	-	0.000	-	-	-	0.001//0.999	-
CYP2C19	rs12268020	T>C	5.823	0.054	1.359	0.891-2.075	0.641	0.414-0.993	2.628	0.688-10.046	1.829//0.401	3.516//0.172
CYP2C19	rs12571421	A>G	2.633	0.268	1.043	0.694-1.567	0.856	0.564-1.3	2.692	0.704-10.288	2.037//0.361	0.521//0.771
CYP2C19	rs12768009	A>G	2.653	0.265	1.080	0.731-1.596	0.817	0.547-1.22	2.010	0.74-5.457	1.711//0.425	0.835//0.659
CYP2C19	rs12769205	A>G	1.040	0.595	1.137	0.756-1.71	0.839	0.551-1.278	1.288	0.439-3.779	0.219//0.896	0.478//0.788
CYP2C19	rs138142612	A>G	49.164	<0.0001	56.634	7.718-415.564	0.000	-	0.954	0.059-15.349	0.959//0.619	217//0
CYP2C19	rs140278421	G>C	2.049	0.359	-	-	0.000	-	0.000	-	94.441//0	-
CYP2C19	rs144036596	A>G	0.387	0.824	1.528	0.253-9.24	0.491	0.044-5.453	0.986	0.061-15.868	52.495//0	95.774//0
CYP2C19	rs145119820	A>G	52.749	<0.0001	58.793	8.026-430.699	0.000	-	-	-	3.085//0.214	217//0
CYP2C19	rs145328984	T>C	48.244	<0.0001	55.579	7.58-407.516	0.000	-	0.991	0.062-15.943	0.972//0.615	217//0
CYP2C19	rs17878459.1	A>G	-	-	-	-	0.000	-	-	-	0.005//0.998	-
CYP2C19	rs17878459.2	C>G	2.304	0.316	0.603	0.142-2.555	2.500	0.48-13.029	0.000	-	52.245//0	0.03//0.985
CYP2C19	rs17878649	A>G	0.298	0.862	1.205	0.603-2.412	0.821	0.403-1.674	1.000	0.062-16.091	0.693//0.707	1.448//0.485
CYP2C19	rs17879685	T>C	53.456	<0.0001	59.632	8.135-437.141	0.000	-	-	-	3.06//0.217	217//0
CYP2C19	rs17884832	T>G	23.850	<0.0001	3.016	1.883-4.832	0.314	0.194-0.508	1.944	0.175-21.604	6.595//0.037	0.395//0.821
CYP2C19	rs17886522	A>C	-	-	1.133	0.551-2.332	0.882	0.429-1.816	-	-	0.364//0.833	0.284//0.868
CYP2C19	rs185136199	T>C	35.348	<0.0001	39.497	5.344-291.948	0.000	-	0.954	0.059-15.349	0.089//0.956	217//0
CYP2C19	rs200003088	A>G	52.565	<0.0001	61.157	8.351-447.862	0.000	-	0.981	0.061-15.794	1.364//0.506	217//0
CYP2C19	rs200936950	A>G	3.007	0.222	4.038	0.448-36.422	0.000	-	1.005	0.062-16.166	33.517//0	216//0
CYP2C19	rs201132803	A>T	-	-	-	-	0.000	-	-	-	0.005//0.998	-
CYP2C19	rs28399505	T>C	-	-	-	-	0.000	-	-	-	0.001//0.999	-
CYP2C19	rs28399513	A>T	2.069	0.355	1.118	0.748-1.672	0.816	0.54-1.232	2.000	0.593-6.744	1.875//0.392	0.296//0.862
CYP2C19	rs28399514	A>G	-	-	2.019	0.182-22.43	-	-	0.495	0.045-5.504	216//0	217//0
CYP2C19	rs375283723	T>C	51.009	<0.0001	-	-	0.000	-	0.000	-	1.18//0.554	-
CYP2C19	rs377184510	A>G	-	-	-	-	0.000	-	-	-	0.001//0.999	-
CYP2C19	rs41291556	T>C	-	-	-	-	0.000	-	-	-	2.563//0.278	-

CYP2C19	rs4244285.1	A>G	1.975	0.372	1.104	0.737-1.654	0.825	0.544-1.25	2.019	0.599-6.808	1.448//0.485	0.521//0.771
CYP2C19	rs4388808	A>G	1.637	0.441	1.089	0.742-1.598	0.826	0.559-1.219	1.378	0.69-2.754	0.592//0.744	0.825//0.662
CYP2C19	rs4417205	G>C	2.152	0.341	1.128	0.752-1.691	0.806	0.532-1.222	2.019	0.599-6.809	1.688//0.43	0.478//0.788
CYP2C19	rs4917612	C>G	2.249	0.325	1.279	0.873-1.873	0.741	0.5-1.097	1.129	0.57-2.237	0.025//0.988	2.605//0.272
CYP2C19	rs4917623	T>C	2.041	0.360	1.464	0.864-2.48	0.895	0.613-1.306	0.909	0.612-1.35	0.979//0.613	0.091//0.955
CYP2C19	rs4986893	A>G	29.430	<0.0001	4.589	2.54-8.29	0.208	0.114-0.381	1.000	0.062-16.092	3.054//0.217	1.435//0.488
CYP2C19	rs545642100	G>C	2.049	0.359	-	-	0.000	-	0.000	-	94.441//0	-
CYP2C19	rs550527959	A>T	-	-	-	-	0.000	-	-	-	0.001//0.999	-
CYP2C19	rs55640102.1	A>C	-	-	-	-	0.000	-	-	-	0.005//0.998	-
CYP2C19	rs55640102.2	T>A	2.106	0.349	-	-	0.000	-	0.000	-	93.108//0	-
CYP2C19	rs55752064	T>C	-	-	-	-	0.000	-	-	-	1.96//0.375	-
CYP2C19	rs55948420	A>G	48.244	<0.0001	55.579	7.58-407.516	0.000	-	0.991	0.062-15.943	0.972//0.615	217//0
CYP2C19	rs559628884	A>C	43.132	<0.0001	26.404	6.302-110.629	0.019	0.003-0.143	0.981	0.061-15.794	0.781//0.677	95.774//0
CYP2C19	rs56337013	T>C	30.327	<0.0001	32.842	4.412-244.472	0.000	-	0.907	0.056-14.606	0.007//0.997	217//0
CYP2C19	rs5787121	D>I	1.016	0.602	2.019	0.182-22.43	0.000	-	0.995	0.062-16.017	95.33//0	217//0
CYP2C19	rs6413438	T>C	3.045	0.218	4.076	0.452-36.773	0.000	-	0.995	0.062-16.017	33.037//0	215//0
CYP2C19	rs72552267	A>G	1.032	0.597	2.028	0.183-22.535	0.000	-	0.991	0.062-15.943	94.885//0	217//0
CYP2C19	rs72558185	D>I	-	-	-	-	-	-	0.000	-	217//0	-
CYP2C19	rs72558186	T>A	-	-	-	-	0.000	-	-	-	0.005//0.998	-
CYP2C19	rs770829708.1	A>G	-	-	-	-	-	-	0.000	-	209//0	-
CYP2C19	rs778258371	A>G	2.049	0.359	3.057	0.315-29.623	0.000	-	0.991	0.062-15.942	52.495//0	216//0
CYP2C19	rs7916649	A>G	0.618	0.734	1.081	0.727-1.609	1.013	0.692-1.483	0.859	0.516-1.43	0.086//0.958	0.001//1
CYP2D6	rs75467367	C>G	-	-	-	-	0.000	-	-	-	0.005//0.998	-
CYP2D6	rs180847475	G>C	-	-	-	-	0.000	-	-	-	0.005//0.998	-
CYP2D6	rs186133763	C>G	3.044	0.218	-	-	0.000	-	0.000	-	52.745//0	-
CYP2D6	rs28371706	A>G	4.592	0.101	-	-	0.000	-	0.000	-	31.596//0	-
CYP2D6	rs140513104	A>G	46.337	<0.0001	55.579	7.58-407.516	0.019	0.003-0.14	0.000	-	0.108//0.947	0.001//0.999
CYP2D6	rs59421388	T>C	-	-	-	-	0.000	-	-	-	2.314//0.315	-
CYP2D6	rs61736512	T>C	-	-	56.842	7.756-416.575	0.018	0.002-0.129	-	-	2.92//0.232	0.001//0.999
CYP2D6	rs28371732	T>C	41.192	<0.0001	18.643	5.688-61.102	0.057	0.017-0.187	0.000	-	0.135//0.935	0.011//0.995

CYP2D6	rs5030866	T>C	2.305	0.316	3.042	0.607-15.244	0.396	0.076-2.065	0.000	-	16.374//0	0.005//0.998
CYP2D6	rs1058172	T>C	-	-	1.394	0.859-2.263	0.717	0.442-1.165	-	-	3.158//0.206	1.785//0.41
CYP2D6	rs1135824	T>C	-	-	-	-	0.000	-	-	-	0.001//0.999	-
CYP2D6	rs1135832.1	A>C	-	-	-	-	-	-	0.000	-	211//0	-
CYP2D6	rs1135832.2	G>C	-	-	-	-	0.000	-	-	-	0.001//0.999	-
CYP2D6	rs1135833	G>C	-	-	-	-	-	-	-	-	-	-
CYP2D6	rs1135835	T>C	48.244	<0.0001	-	-	0.000	-	0.000	-	0.874//0.646	-
CYP2D6	rs1135838	A>C	-	-	54.382	7.413-398.967	0.018	0.003-0.135	-	-	2.685//0.261	0.001//0.999
CYP2D6	rs118203758	T>C	3.070	0.215	4.095	0.454-36.939	0.000	-	0.991	0.062-15.943	33.197//0	217//0
CYP2D6	rs138100349	A>G	52.285	<0.0001	-	-	0.000	-	0.000	-	1.236//0.539	-
CYP2D6	rs138417770	T>C	-	-	1.019	0.063-16.396	-	-	0.981	0.061-15.794	213//0	217//0
CYP2D6	rs139779104	T>C	3.681	0.159	6.085	0.726-50.975	0.198	0.023-1.71	0.000	-	16.374//0	0.001//0.999
CYP2D6	rs142302759	T>G	-	-	-	-	0.000	-	-	-	2.826//0.243	-
CYP2D6	rs146540061	A>G	-	-	-	-	0.000	-	-	-	2.534//0.282	-
CYP2D6	rs146838345	T>C	2.075	0.354	3.071	0.317-29.761	0.000	-	0.986	0.061-15.868	52.495//0	217//0
CYP2D6	rs147943410	C>G	-	-	-	-	0.000	-	-	-	0.019//0.991	-
CYP2D6	rs147960066	A>G	-	-	5.168	0.599-44.614	0.193	0.022-1.67	-	-	0.03//0.985	0.001//0.999
CYP2D6	rs148250267	T>C	-	-	-	-	0.000	-	-	-	2.669//0.263	-
CYP2D6	rs148382141	T>C	-	-	-	-	0.000	-	-	-	3.05//0.218	-
CYP2D6	rs149157808	T>C	-	-	-	-	0.000	-	-	-	2.954//0.228	-
CYP2D6	rs149686350	A>G	-	-	58.105	7.932-425.633	0.017	0.002-0.126	-	-	3.05//0.218	0.001//0.999
CYP2D6	rs150445731	A>G	-	-	61.902	8.452-453.355	0.016	0.002-0.118	-	-	3.314//0.191	0.001//0.999
CYP2D6	rs188062577	T>C	49.240	<0.0001	56.842	7.756-416.575	0.000	-	0.995	0.062-16.017	1.074//0.585	217//0
CYP2D6	rs199722016	T>C	-	-	-	-	0.000	-	-	-	0.001//0.999	-
CYP2D6	rs199849357	A>G	3.007	0.222	-	-	0.000	-	0.000	-	53.245//0	-
CYP2D6	rs201297021	T>C	3.182	0.204	5.294	0.613-45.704	0.237	0.026-2.141	0.000	-	21.889//0	0.001//0.999
CYP2D6	rs201377835	C>G	-	-	-	-	0.000	-	-	-	0.011//0.995	-
CYP2D6	rs267608279	D>I	2.049	0.359	-	-	0.000	-	0.000	-	94.441//0	-
CYP2D6	rs267608285	T>G	50.791	<0.0001	-	-	0.000	-	0.000	-	0.204//0.903	-
CYP2D6	rs267608289	T>C	-	-	0.493	0.122-1.997	2.029	0.501-8.219	-	-	0.011//0.995	0.043//0.979

CYP2D6	rs267608290	T>G	-	-	-	-	0.000	-	-	-	2.654//0.265	-
CYP2D6	rs267608291	T>C	-	-	3.028	0.312-29.344	0.330	0.034-3.2	-	-	0.011//0.995	0.001//0.999
CYP2D6	rs267608295.2	G>C	-	-	-	-	0.000	-	-	-	0.005//0.998	-
CYP2D6	rs267608295.3	T>G	-	-	-	-	0.000	-	-	-	0.005//0.998	-
CYP2D6	rs267608297	A>G	6.444	0.040	-	-	0.000	-	0.000	-	49.481//0	-
CYP2D6	rs267608298	A>G	1.281	0.527	2.097	0.189-23.304	0.958	0.06-15.423	0.000	-	91.774//0	0.001//0.999
CYP2D6	rs267608302	T>G	-	-	-	-	0.000	-	-	-	0.005//0.998	-
CYP2D6	rs267608305	T>C	-	-	-	-	0.000	-	-	-	2.794//0.247	-
CYP2D6	rs267608308	T>C	43.409	<0.0001	27.694	6.619-115.877	0.037	0.009-0.156	0.000	-	0.985//0.611	0.005//0.998
CYP2D6	rs267608309	A>G	2.995	0.224	-	-	0.000	-	0.000	-	52.995//0	-
CYP2D6	rs267608310	A>G	1.361	0.506	3.042	0.314-29.483	0.495	0.045-5.504	0.000	-	52.745//0	0.001//0.999
CYP2D6	rs267608313	A>G	6.031	0.049	-	-	0.000	-	0.000	-	16.374//0	-
CYP2D6	rs267608316	A>C	-	-	-	-	0.000	-	-	-	2.842//0.241	-
CYP2D6	rs267608322	A>C	2.009	0.366	1.005	0.062-16.166	0.000	-	-	-	0.001//0.999	217//0
CYP2D6	rs28371706.1	A>G	2.555	0.279	2.257	0.203-25.09	0.891	0.055-14.345	0.000	-	83.329//0	0.001//0.999
CYP2D6	rs28371717	A>C	2.003	0.367	2.355	0.601-9.231	0.498	0.123-2.016	0.000	-	12.14//0.002	0.011//0.995
CYP2D6	rs28371725	T>C	16.001	<0.0001	2.698	1.63-4.467	0.379	0.229-0.629	0.000	-	3.493//0.174	0.986//0.611
CYP2D6	rs28371729	T>G	-	-	57.249	7.811-419.59	0.017	0.002-0.128	-	-	2.954//0.228	0.001//0.999
CYP2D6	rs28371733	T>C	50.489	<0.0001	-	-	0.000	-	0.000	-	1.087//0.581	-
CYP2D6	rs28371735	A>G	-	-	-	-	0.000	-	-	-	4.19//0.123	-
CYP2D6	rs28371736.1	A>G	-	-	-	-	0.000	-	-	-	0.001//0.999	-
CYP2D6	rs28371736.2	G>C	-	-	-	-	0.000	-	-	-	0.001//0.999	-
CYP2D6	rs35742686	I>D	47.356	<0.0001	56.912	7.765-417.108	0.018	0.002-0.133	0.000	-	1.087//0.581	0.001//0.999
CYP2D6	rs367543000	A>G	2.007	0.367	4.037	0.448-36.419	0.332	0.034-3.215	0.000	-	33.677//0	0.001//0.999
CYP2D6	rs368389952	T>C	4.986	0.083	-	-	0.000	-	0.000	-	23//0	-
CYP2D6	rs370580423	A>G	4.012	0.135	3.028	0.312-29.342	-	-	0.000	-	217//0	0.001//0.999
CYP2D6	rs371181941	A>G	50.235	<0.0001	-	-	0.000	-	0.000	-	1.074//0.585	-
CYP2D6	rs371793722	A>G	-	-	-	-	0.000	-	-	-	1.478//0.478	-
CYP2D6	rs377591409	A>G	1.361	0.506	3.042	0.314-29.48	0.495	0.045-5.504	0.000	-	52.995//0	0.001//0.999
CYP2D6	rs5030862	T>C	1.016	0.602	2.019	0.182-22.43	0.000	-	0.995	0.062-16.017	95.33//0	217//0

CYP2D6	rs5030867	T>G	-	-	4.038	0.448-36.422	0.248	0.027-2.234	-	-	0.019//0.991	0.001//0.999
CYP2D6	rs536049502	T>A	-	-	-	-	0.000	-	-	-	0.011//0.995	-
CYP2D6	rs544790460	A>C	-	-	-	-	0.000	-	-	-	2.81//0.245	-
CYP2D6	rs554540676	T>G	-	-	-	-	0.000	-	-	-	2.937//0.23	-
CYP2D6	rs554875652	A>C	3.007	0.222	4.037	0.448-36.419	1.005	0.062-16.165	0.000	-	159.293//0	0.001//0.999
CYP2D6	rs567606867	T>C	50.235	<0.0001	-	-	0.000	-	0.000	-	0.053//0.974	-
CYP2D6	rs569926140	A>C	-	-	-	-	0.000	-	-	-	2.989//0.224	-
CYP2D6	rs67497403	A>G	-	-	3.028	0.312-29.342	0.330	0.034-3.2	-	-	0.011//0.995	0.001//0.999
CYP2D6	rs72549346	I>D	-	-	-	-	0.000	-	-	-	0.005//0.998	-
CYP2D6	rs72549349	C>G	-	-	-	-	0.000	-	-	-	0.005//0.998	-
CYP2D6	rs72549351	I>D	-	-	-	-	0.000	-	-	-	2.92//0.232	-
CYP2D6	rs72549354	D>I	-	-	-	-	0.000	-	-	-	0.001//0.999	-
CYP2D6	rs72549358	T>C	-	-	61.714	8.422-452.229	0.016	0.002-0.119	-	-	3.234//0.198	0.001//0.999
CYP2D6	rs730882170	D>I	2.101	0.350	4.115	0.456-37.122	0.326	0.034-3.155	0.000	-	32.877//0	0.001//0.999
CYP2D6	rs74478221	T>C	2.045	0.360	4.078	0.452-36.792	0.000	-	0.329	0.034-3.185	153.415//0	211//0
CYP2D6	rs745746329	T>C	47.559	<0.0001	56.237	7.669-412.366	0.000	-	0.242	0.027-2.182	0.789//0.674	217//0
CYP2D6	rs747998333	A>G	-	-	-	-	0.000	-	-	-	0.045//0.978	-
CYP2D6	rs74802369	T>A	-	-	-	-	0.000	-	-	-	0.011//0.995	-
CYP2D6	rs748712690	T>C	-	-	-	-	0.000	-	-	-	1.562//0.458	-
CYP2D6	rs74962936	A>G	2.007	0.367	4.037	0.448-36.419	0.332	0.034-3.215	0.000	-	33.677//0	0.001//0.999
CYP2D6	rs750996195	T>C	-	-	55.906	7.624-409.926	0.018	0.002-0.131	-	-	2.81//0.245	0.001//0.999
CYP2D6	rs758320086	I>D	-	-	-	-	0.000	-	-	-	0.001//0.999	-
CYP2D6	rs76187628	A>G	-	-	15.185	4.604-50.081	0.066	0.02-0.217	-	-	2.152//0.341	0.011//0.994
CYP2D6	rs765776661	I>D	-	-	3.028	0.312-29.342	0.330	0.034-3.2	-	-	0.011//0.995	0.001//0.999
CYP2D6	rs766507177	T>G	-	-	-	-	0.000	-	-	-	2.563//0.278	-
CYP2D6	rs77312092	T>C	-	-	-	-	0.000	-	-	-	0.019//0.99	-
CYP2D6	rs773790593	A>G	49.240	<0.0001	-	-	0.000	-	0.000	-	0.972//0.615	-
CYP2D6	rs774943042	A>C	48.244	<0.0001	-	-	0.000	-	0.000	-	0.874//0.646	-
CYP2D6	rs78482768	C>G	3.007	0.222	-	-	0.000	-	0.000	-	53.245//0	-
CYP2D6	rs79292917	T>C	48.385	<0.0001	58.249	7.951-426.729	0.020	0.003-0.146	0.000	-	1.661//0.436	0.001//0.999

CYP2D6	rs79392742	T>G	3.016	0.221	2.019	0.182-22.43	0.000	-	-	-	0.005//0.998	217//0
CYP2D6	rs79738337	A>G	1.054	0.590	2.038	0.183-22.645	0.986	0.061-15.868	0.000	-	93.996//0	0.001//0.999
CYP2D6	rs5030655	I>D	-	-	-	-	0.000	-	-	-	3.234//0.198	-
CYP2D6	rs747955910	I>D	1.345	0.510	3.028	0.312-29.342	0.498	0.045-5.53	0.000	-	53.245//0	0.001//0.999
GCLC	rs114704032	T>C	-	-	-	-	0.000	-	-	-	0.001//0.999	-
GCLC	rs12524550	T>C	12.103	0.002	4.474	1.458-13.727	0.167	0.048-0.586	-	-	0.319//0.853	33.037//0
GCLC	rs12524652	T>C	42.219	<0.0001	11.850	4.609-30.467	0.067	0.024-0.19	-	-	3.295//0.193	22.334//0
GCLC	rs17193216	A>G	-	-	1.449	0.541-3.88	0.690	0.258-1.847	-	-	0.121//0.941	0.058//0.971
GCLC	rs17884118	T>C	-	-	0.600	0.142-2.543	1.667	0.393-7.063	-	-	0.011//0.995	0.029//0.985
GCLC	rs2397147	T>C	5.380	0.068	0.670	0.447-1.006	1.321	0.87-2.007	2.341	0.81-6.767	0.031//0.985	0.276//0.871
GCLC	rs41271285	T>C	-	-	2.000	0.18-22.223	0.500	0.045-5.556	-	-	0.005//0.998	0.001//0.999
GCLC	rs41271289	T>C	48.358	<0.0001	28.456	6.806-118.981	0.017	0.002-0.128	-	-	2.937//0.23	95.774//0
GCLC	rs77516417	A>C	-	-	30.665	7.341-128.094	0.033	0.008-0.136	-	-	3.314//0.191	0.005//0.998
GCLC	rs12525474	T>C	4.476	0.107	1.452	0.97-2.173	0.653	0.433-0.985	1.457	0.405-5.24	3.717//0.156	0.007//0.997
GCLC	rs1555906	A>G	3.567	0.168	1.423	0.968-2.091	0.714	0.487-1.047	0.969	0.525-1.787	1.542//0.462	0.252//0.881
GCLC	rs17883718	A>G	51.231	<0.0001	-	-	0.000	-	0.000	-	1.18//0.554	-
GCLC	rs17884046	A>C	0.076	0.963	0.932	0.544-1.597	1.075	0.623-1.854	0.995	0.062-16.017	0.013//0.993	0.061//0.97
GCLC	rs3799694	T>C	5.051	0.080	1.532	1.046-2.246	0.748	0.51-1.096	0.703	0.381-1.296	0.062//0.97	0.286//0.867
GCLC	rs3799700	T>C	2.077	0.354	0.770	0.523-1.133	1.324	0.891-1.967	0.971	0.411-2.29	0.124//0.94	0.864//0.649
GCLC	rs4715407	A>G	11.464	0.003	1.876	1.261-2.791	0.557	0.372-0.834	0.693	0.268-1.793	3.201//0.202	0.019//0.99
GCLC	rs524553	T>C	16.487	<0.0001	2.203	1.456-3.334	0.421	0.275-0.643	1.690	0.399-7.162	7.098//0.029	0.702//0.704
GCLC	rs547222	T>C	7.150	0.028	1.641	1.102-2.443	0.621	0.414-0.931	0.783	0.279-2.201	2.356//0.308	0.028//0.986
GCLC	rs648595	T>G	7.482	0.024	1.487	0.94-2.353	0.591	0.401-0.869	1.417	0.894-2.248	10.671//0.005	0.225//0.894
GCLC	rs680403	T>C	1.866	0.393	1.153	0.75-1.771	0.852	0.578-1.256	1.064	0.669-1.692	0.033//0.984	0.914//0.633
GCLC	rs761142	A>C	3.788	0.150	1.301	0.888-1.908	0.695	0.474-1.019	1.368	0.697-2.684	4.761//0.093	0.101//0.951
GCLC	rs9474588	A>G	1.796	0.407	1.281	0.875-1.875	0.785	0.537-1.148	0.990	0.549-1.784	0.436//0.804	0.421//0.81
GCLC	RS17883901	C>T	1.577	0.454	1.343	0.83-2.174	0.735	0.449-1.201	1.028	0.144-7.366	0.239//0.887	0.074//0.964
GCLM	rs17376966	T>C	-	-	0.664	0.11-4.011	1.507	0.249-9.11	-	-	0.005//0.998	0.011//0.995
GCLM	rs3789453	T>C	0.275	0.872	0.910	0.617-1.341	1.084	0.729-1.611	1.114	0.422-2.944	0.289//0.865	0.43//0.806
GCLM	rs41303970	C>T	6.689	0.035	1.286	0.819-2.019	0.639	0.398-1.027	4.152	0.872-19.777	0.885//0.642	10.069//0.057

GPX4	rs117193629	T>C	5.309	0.070	0.440	0.21-0.923	2.395	1.111-5.163	0.977	0.061-15.72	4.299//0.117	0.123//0.941
GPX4	rs73507255	A>G	-	-	-	-	0.000	-	-	-	2.778//0.249	-
GPX4	rs76201145	A>G	3.007	0.222	4.037	0.448-36.419	0.000	-	1.005	0.062-16.165	33.677//0	217//0
GPX4	rs713041	C>T	16.735	<0.0001	0.538	0.349-0.829	2.212	1.507-3.245	0.610	0.375-0.992	1.999//0.368	18.12//0
GSTM1	rs185525957	A>G	2.340	0.310	4.037	0.448-36.419	0.000	-	0.500	0.045-5.555	95.542//0	217//0
GSTM1	rs2239892	A>G	-	-	2.987	1.8-4.957	0.335	0.202-0.555	-	-	6.025//0.049	0.881//0.644
GSTM1	null	I>D	-	-	0.880	0.60-1.28	-	-	1.140	0.78-1.67	210//0	220//0
GSTP1	rs1138272	T>C	8.980	0.011	0.353	0.172-0.727	3.086	1.41-6.753	1.479	0.245-8.941	17.41//0	3.274//0.195
GSTP1	rs11553890	T>C	4.002	0.135	-	-	0.000	-	0.000	-	33.677//0	-
GSTP1	rs11553892.1	A>C	-	-	-	-	0.000	-	-	-	0.011//0.995	-
GSTP1	rs11553892.2	G>C	4.040	0.133	-	-	0.000	-	0.000	-	94.653//0	-
GSTP1	rs11553893	T>G	49.044	<0.0001	56.647	7.729-415.176	0.000	-	1.000	0.062-16.092	1.087//0.581	215//0
GSTP1	rs12796085	T>C	39.492	<0.0001	47.058	6.396-346.245	0.022	0.003-0.162	0.000	-	0.441//0.802	0.001//0.999
GSTP1	rs1871042	T>C	6.885	0.032	0.658	0.447-0.968	1.271	0.857-1.886	2.514	1.021-6.195	0.535//0.765	0.033//0.984
GSTP1	rs41462048	A>G	0.998	0.607	2.000	0.18-22.221	1.005	0.062-16.165	0.000	-	96.219//0	0.001//0.999
GSTP1	rs4147581	G>C	6.140	0.046	0.641	0.426-0.964	1.087	0.745-1.587	1.583	0.989-2.534	0.307//0.858	0.221//0.896
GSTP1	rs45543438	A>G	2.012	0.366	3.028	0.312-29.342	0.000	-	1.000	0.062-16.091	53.245//0	217//0
GSTP1	rs4986949	T>G	48.616	<0.0001	16.755	5.922-47.405	0.046	0.014-0.15	0.944	0.059-15.196	1.806//0.405	33.357//0
GSTP1	rs749174	A>G	2.282	0.319	0.824	0.558-1.215	1.111	0.746-1.653	1.452	0.663-3.181	0.031//0.985	0.033//0.984
GSTP1	rs762803	A>C	5.544	0.063	0.809	0.549-1.193	0.973	0.657-1.442	2.098	1.028-4.282	0.757//0.685	0.796//0.672
GSTP1	rs8191439	A>G	1.043	0.594	0.923	0.383-2.22	0.981	0.4-2.406	-	-	0.123//0.94	4.436//0.109
GSTP1	rs8191441	A>G	1.005	0.605	1.507	0.249-9.111	0.000	-	1.000	0.14-7.165	136.792//0	215//0
GSTP1	rs8191444	G>C	-	-	-	-	0.000	-	-	-	0.005//0.998	-
GSTP1	RS1695	A>G	6.740	0.034	0.644	0.441-0.941	1.310	0.891-1.927	2.050	0.936-4.489	0.205//0.903	0.017//0.992
GSTT1	null	I>D	-	-	1.100	0.75-1.62	-	-	0.910	0.62-1.34	210//0	217//0
NAT2	rs76846598	T>C	0.010	0.995	0.986	0.243-3.994	1.014	0.202-5.081	1.014	0.063-16.317	33.677//0	33.197//0
NAT2	rs146789770	A>C	0.995	0.608	1.991	0.179-22.119	0.000	-	1.009	0.063-16.241	96.219//0	216//0
NAT2	rs150339859	T>C	47.249	<0.0001	54.316	7.404-398.457	0.000	-	0.986	0.061-15.868	0.874//0.646	217//0
NAT2	rs45594437	A>G	3.683	0.159	0.400	0.077-2.085	5.024	0.582-43.364	0.000	-	94.441//0	0.029//0.985
NAT2	rs1041983	T>C	9.000	0.011	1.795	1.205-2.675	0.743	0.508-1.087	0.646	0.387-1.078	0.046//0.977	0.813//0.666

NAT2	rs1208	A>G	5.712	0.058	0.642	0.432-0.954	1.425	0.948-2.14	1.793	0.701-4.588	0.001//0.999	0.033//0.984
NAT2	rs12720065	G>C	3.044	0.218	-	-	0.000	-	0.000	-	52.745//0	-
NAT2	rs138707146	T>C	43.983	<0.0001	27.003	6.448-113.086	0.019	0.003-0.139	0.991	0.062-15.942	0.91//0.635	93.996//0
NAT2	rs139351995	A>C	-	-	-	-	0.000	-	-	-	0.001//0.999	-
NAT2	rs144176822	T>G	3.023	0.221	4.057	0.45-36.594	0.000	-	1.000	0.062-16.092	33.357//0	216//0
NAT2	rs149283608	A>T	-	-	-	-	0.000	-	-	-	0.011//0.995	-
NAT2	rs1799929	T>C	1.401	0.496	1.066	0.719-1.581	0.934	0.625-1.395	1.010	0.382-2.673	1.007//0.605	0.574//0.751
NAT2	rs1799930	A>G	9.188	0.010	1.745	1.185-2.571	0.562	0.379-0.834	1.003	0.493-2.044	2.408//0.3	1.553//0.46
NAT2	rs1799931	A>G	19.376	<0.0001	2.613	1.69-4.041	0.398	0.256-0.621	0.387	0.074-2.017	2.733//0.255	0.032//0.984
NAT2	rs1801279	A>G	3.023	0.221	4.056	0.45-36.59	0.000	-	1.000	0.062-16.091	33.517//0	217//0
NAT2	rs1801280	T>C	1.421	0.491	0.827	0.564-1.212	1.126	0.762-1.664	1.529	0.612-3.819	0.627//0.731	0.189//0.91
NAT2	rs1805158	T>C	3.007	0.222	4.037	0.448-36.419	0.000	-	1.005	0.062-16.165	33.677//0	217//0
NAT2	rs45477599	T>A	-	-	-	-	0.000	-	-	-	0.011//0.995	-
NAT2	rs45518335	T>C	1.054	0.590	2.038	0.183-22.645	0.000	-	0.986	0.061-15.868	93.996//0	216//0
NAT2	rs45607939	T>A	1.361	0.506	3.042	0.314-29.48	0.495	0.045-5.504	0.000	-	52.995//0	0.001//0.999
NAT2	rs45618543	T>G	45.118	<0.0001	27.661	6.611-115.729	0.019	0.003-0.136	0.991	0.062-15.943	0.972//0.615	95.774//0
NAT2	rs4986996	A>G	-	-	19.441	2.563-147.482	0.051	0.007-0.39	-	-	0.384//0.825	0.001//0.999
NAT2	rs4986997	A>T	2.000	0.368	-	-	0.000	-	0.000	-	95.33//0	-
NAT2	rs55700793	A>G	-	-	-	-	0.000	-	-	-	0.001//0.999	-
NAT2	rs56011192	T>C	1.000	0.607	2.009	0.364-11.087	0.332	0.034-3.215	1.005	0.062-16.166	33.517//0	95.33//0
NAT2	rs56054745.1	A>C	-	-	2.038	0.369-11.245	0.491	0.089-2.708	-	-	0.019//0.991	0.005//0.998
NAT2	rs56054745.2	A>G	-	-	-	-	0.000	-	-	-	0.011//0.995	-
NAT2	rs56387565	T>C	-	-	-	-	0.000	-	-	-	0.005//0.998	-
NAT2	rs56393504	A>G	45.422	<0.0001	27.893	6.665-116.723	0.018	0.003-0.135	0.986	0.061-15.867	1.01//0.604	94.885//0
NAT2	rs72466459	T>C	54.531	<0.0001	63.235	8.623-463.745	0.000	-	0.916	0.057-14.749	1.339//0.512	216//0
NAT2	rs72466460	T>C	57.832	<0.0001	-	-	0.000	-	0.000	-	1.585//0.453	-
NAT2	rs72466461	A>G	-	-	-	-	0.000	-	-	-	0.005//0.998	-
NAT2	rs72554615	T>C	-	-	-	-	0.000	-	-	-	2.904//0.234	-
NAT2	rs72554616	A>C	-	-	-	-	0.000	-	-	-	0.001//0.999	-
NAT2	rs72554617	A>G	45.577	<0.0001	15.739	5.567-44.5	0.049	0.015-0.159	0.991	0.062-15.942	1.619//0.445	33.357//0

NAT2	rs79050330	T>C	56.155	<0.0001	65.617	8.956-480.752	0.000	-	0.930	0.058-14.973	1.566//0.457	216//0
NFE2	rs10506328	A>C	0.170	0.919	0.924	0.608-1.406	1.089	0.706-1.68	0.990	0.314-3.121	0.319//0.853	0.062//0.97
NFE2L1	rs147114188	A>G	38.120	<0.0001	6.714	3.409-13.223	0.138	0.068-0.279	0.995	0.062-16.017	2.869//0.238	4.436//0.109
NFE2L1	rs150840650	T>C	2.012	0.366	3.028	0.312-29.342	0.000	-	1.000	0.062-16.091	53.245//0	217//0
NFE2L1	rs2023885	A>G	11.179	0.004	1.955	1.301-2.938	0.548	0.362-0.828	0.404	0.103-1.585	2.35//0.309	0.66//0.719
NFE2L1	rs2229367	G>C	-	-	3.131	0.323-30.345	0.319	0.033-3.095	-	-	0.011//0.995	0.001//0.999
NFE2L2	rs10930781	A>G	3.030	0.220	0.941	0.634-1.397	0.950	0.633-1.424	1.609	0.694-3.731	0.235//0.889	0.658//0.72
NFE2L2	rs11686945	T>C	3.378	0.185	0.703	0.476-1.039	1.318	0.88-1.976	1.515	0.665-3.451	0.729//0.695	0.437//0.804
NFE2L2	rs13001694	A>G	2.134	0.344	0.779	0.522-1.163	1.192	0.79-1.799	1.835	0.605-5.566	0.161//0.923	0.014//0.993
NFE2L2	rs199673454	A>T	-	-	-	-	0.000	-	-	-	0.001//0.999	-
NFE2L2	rs2001350	T>C	2.588	0.274	0.903	0.609-1.339	0.954	0.633-1.438	1.842	0.802-4.231	0.023//0.989	3.584//0.167
NFE2L2	rs34468415	A>G	2.283	0.319	0.731	0.485-1.102	1.336	0.874-2.042	1.333	0.455-3.91	0.334//0.846	0.013//0.994
NFE2L3	rs11770841	T>G	1.087	0.581	1.094	0.733-1.632	0.830	0.568-1.212	1.220	0.726-2.052	1.825//0.401	0.009//0.996
NFE2L3	rs2237329	T>C	15.339	<0.0001	2.219	1.464-3.363	0.434	0.283-0.666	0.962	0.305-3.032	2.762//0.251	1.551//0.46
NFE2L3	rs79693596	A>G	7.608	0.022	2.212	0.925-5.285	0.336	0.129-0.876	-	-	0.338//0.844	32.307//0
NFE2L3	rs12113404	A>G	19.444	<0.0001	2.267	1.542-3.334	0.425	0.287-0.628	1.071	0.504-2.278	8.484//0.014	1.717//0.424
NQO1	rs117363962	T>C	43.586	<0.0001	49.767	6.773-365.708	0.000	-	0.986	0.061-15.867	0.597//0.742	215//0
NQO1	rs143567674	T>C	54.717	<0.0001	63.849	8.717-467.698	0.000	-	0.949	0.059-15.274	1.478//0.478	217//0
NQO1	rs45467396	A>G	-	-	0.493	0.089-2.72	2.028	0.368-11.192	-	-	0.005//0.998	0.019//0.991
NQO1	rs76921462	T>C	10.570	0.005	1.959	1.226-3.133	0.471	0.292-0.762	2.887	0.298-27.985	3.353//0.187	1.055//0.59
NQO1	rs77097817	T>C	0.550	0.760	1.529	0.425-5.497	0.589	0.139-2.495	0.991	0.062-15.942	15.966//0	33.197//0
NQO1	rs10517	A>G	0.413	0.814	1.030	0.699-1.519	0.957	0.646-1.419	1.111	0.367-3.363	2.748//0.253	1.868//0.393
NQO1	rs1131341	G>C	-	-	-	-	0.000	-	-	-	0.011//0.995	-
NQO1	rs1131341.1	A>G	-	-	1.635	0.692-3.862	0.612	0.259-1.446	-	-	0.248//0.883	0.098//0.952
NQO1	rs114238154	A>G	-	-	-	-	0.000	-	-	-	0.005//0.998	-
NQO1	rs1800566	A>G	10.772	0.005	2.012	1.316-3.076	0.592	0.402-0.871	0.866	0.496-1.515	15.371//0	1.043//0.594
NQO1	rs201787127	T>C	26.269	<0.0001	29.032	3.896-216.332	0.000	-	0.972	0.06-15.646	0.056//0.972	217//0
NQO1	rs2917670	T>C	0.995	0.608	1.101	0.745-1.628	0.838	0.574-1.224	1.213	0.687-2.141	2.174//0.337	0.012//0.994
NQO1	rs2917677	T>C	10.578	0.005	1.743	1.189-2.557	0.529	0.36-0.78	1.437	0.601-3.437	11.507//0.003	0.008//0.996
NQO1	rs34447156	C>G	-	-	4.075	0.452-36.764	0.245	0.027-2.213	-	-	0.019//0.991	0.001//0.999

PON1	rs3917592	T>C	-	-	0.972	0.06-15.646	-	-	1.029	0.064-16.552	217//0	211//0
PON1	rs78307684	T>G	47.221	<0.0001	54.414	7.42-399.062	0.000	-	1.014	0.063-16.325	0.997//0.607	210//0
PON1	rs854570	A>C	4.991	0.082	1.527	0.974-2.395	0.971	0.663-1.422	0.689	0.443-1.072	0.144//0.931	0.017//0.992
PON1	rs13306698	T>C	48.617	<0.0001	5.736	2.972-11.071	0.000	-	1.107	0.478-2.566	10.022//0.007	216//0
PON1	rs199851417	A>G	44.024	<0.0001	47.721	6.486-351.099	0.000	-	-	-	2.078//0.354	217//0
PON1	rs2057681	A>G	3.495	0.174	1.406	0.941-2.102	0.710	0.486-1.036	1.075	0.648-1.783	2.5//0.287	0.447//0.8
PON1	rs2299260	T>C	1.830	0.401	0.798	0.539-1.182	1.167	0.78-1.745	1.683	0.601-4.715	0.344//0.842	0.031//0.985
PON1	rs3917477	A>G	1.308	0.520	1.124	0.658-1.919	0.855	0.499-1.466	-	-	1.486//0.476	0.013//0.993
PON1	rs3917503	T>C	5.403	0.067	1.626	1.07-2.469	0.709	0.485-1.035	0.913	0.573-1.456	1.383//0.501	1.127//0.569
PON1	rs3917550	A>G	3.751	0.153	1.558	0.936-2.596	0.596	0.349-1.017	1.327	0.293-6.001	0.148//0.929	6.293//0.043
PON1	rs3917594	T>C	2.995	0.224	4.019	0.446-36.251	0.000	-	1.009	0.063-16.241	33.677//0	216//0
PON1	rs662	T>C	3.575	0.167	1.427	0.951-2.143	0.719	0.491-1.053	1.028	0.619-1.706	2.021//0.364	0.447//0.8
PON1	rs854552	T>C	7.706	0.021	1.527	1.042-2.237	0.587	0.401-0.859	1.514	0.728-3.148	11.344//0.003	0.006//0.997
PON1	rs854555	A>C	8.098	0.017	1.128	0.68-1.869	0.595	0.405-0.874	1.715	1.123-2.619	8.271//0.016	0.288//0.866
PON1	rs854562	T>C	3.013	0.222	1.282	0.844-1.948	0.782	0.51-1.2	0.882	0.251-3.093	0.584//0.747	0.033//0.984
PON1	rs854565	A>G	4.941	0.085	1.204	0.803-1.807	0.680	0.463-0.997	1.591	0.907-2.791	8.849//0.012	0.001//1
PON1	rs854568	A>G	3.971	0.137	1.813	0.868-3.783	0.741	0.506-1.085	1.139	0.781-1.661	4.163//0.125	0.481//0.786
PON2	rs375489124	T>C	21.889	<0.0001	17.315	2.228-134.599	0.000	-	0.145	0.017-1.254	51.447//0	216//0
PON2	rs144782675	T>C	-	-	0.000	-	-	-	-	-	-	0.001//0.999
PON2	rs12534274	A>G	11.173	0.004	2.013	1.328-3.05	0.646	0.44-0.948	0.734	0.439-1.23	3.729//0.155	0.129//0.937
PON2	rs17717505	A>C	65.959	<0.0001	-	-	0.000	-	0.000	-	2.641//0.267	-
PON2	rs10261470	A>G	3.465	0.177	0.781	0.417-1.462	1.479	0.764-2.862	0.300	0.031-2.91	10.101//0.006	0.026//0.987
PON2	rs11981433	T>C	2.713	0.258	0.970	0.645-1.458	0.946	0.626-1.429	4.976	0.576-42.958	4.912//0.086	0.395//0.821
PON2	rs201301042	T>C	44.263	<0.0001	50.526	6.876-371.281	0.000	-	0.972	0.06-15.646	0.606//0.739	217//0
PON2	rs2299267	A>G	7.943	0.019	1.611	1.095-2.369	0.584	0.396-0.861	1.179	0.603-2.303	3.549//0.17	1.424//0.491
PON2	rs7493	G>C	0.303	0.860	1.010	0.692-1.474	1.049	0.717-1.535	0.833	0.424-1.638	0.033//0.984	0.686//0.71
PON2	rs77619496	A>G	47.249	<0.0001	54.316	7.404-398.457	0.000	-	0.986	0.061-15.868	0.874//0.646	217//0
PON2	rs7785039	T>C	3.040	0.219	0.881	0.584-1.329	1.076	0.709-1.632	2.217	0.425-11.562	3.083//0.214	1.48//0.477
PON3	rs377433561	T>C	0.366	0.833	1.521	0.252-9.196	0.991	0.062-15.943	0.493	0.044-5.478	136.792//0	95.774//0
PON3	rs138268669	A>G	39.147	<0.0001	7.404	3.656-14.995	0.127	0.061-0.266	0.493	0.044-5.477	1.462//0.481	5.265//0.072

PON3	rs17883823	T>C	-	-	-	-	0.000	-	-	-	2.654//0.265	-
PON3	rs368482537	A>G	-	-	-	-	0.000	-	-	-	3.033//0.22	-
PON3	rs763839908	T>C	-	-	-	-	0.000	-	-	-	2.904//0.234	-
PON3	rs17883750	A>G	-	-	0.814	0.441-1.504	1.228	0.665-2.267	-	-	0.558//0.756	0.815//0.665
PON3	rs17885558	A>G	15.324	<0.0001	2.639	1.572-4.431	0.372	0.22-0.629	0.907	0.056-14.6	2.49//0.288	0.033//0.984
PON3	rs10953143	T>C	2.774	0.250	1.473	0.878-2.472	0.795	0.543-1.166	1.016	0.676-1.526	2.296//0.317	0.179//0.914
PON3	rs139856535	T>C	1.016	0.602	2.019	0.182-22.431	0.000	-	0.995	0.062-16.017	94.885//0	216//0
PON3	rs141350740	A>G	-	-	0.606	0.246-1.494	1.649	0.669-4.063	-	-	0.078//0.962	0.208//0.901
PON3	rs142620825	A>G	-	-	-	-	0.000	-	-	-	0.001//0.999	-
PON3	rs17883013	T>G	47.001	<0.0001	-	-	0.000	-	0.000	-	0.77//0.68	-
SOD1	rs114905802	T>C	1.033	0.597	2.028	0.183-22.538	0.000	-	0.991	0.062-15.942	94.441//0	216//0
SOD1	rs138002121	G>C	-	-	-	-	0.000	-	-	-	0.019//0.99	-
SOD1	rs183603801	C>G	-	-	-	-	0.000	-	-	-	0.001//0.999	-
SOD1	rs76067554	G>C	1.217	0.544	0.735	0.23-2.354	1.160	0.349-3.861	-	-	0.031//0.985	12.077//0.002
SOD1	rs1041740	T>C	6.539	0.038	1.654	1.116-2.453	0.651	0.445-0.952	0.911	0.52-1.599	3.674//0.159	0.154//0.926
SOD1	rs121912438	G>C	-	-	-	-	0.000	-	-	-	0.011//0.995	-
SOD1	rs121912441	T>C	-	-	-	-	0.000	-	-	-	0.001//0.999	-
SOD1	rs121912442	T>C	2.000	0.368	3.014	0.311-29.206	0.000	-	1.005	0.062-16.166	53.245//0	216//0
SOD1	rs121912443	A>G	43.267	<0.0001	-	-	0.000	-	0.000	-	0.449//0.799	-
SOD1	rs121912451	A>G	50.644	<0.0001	58.720	8.01-430.455	0.000	-	0.972	0.06-15.642	1.169//0.557	215//0
SOD1	rs121912455	A>G	1.005	0.605	2.009	0.181-22.325	0.000	-	1.000	0.062-16.091	95.774//0	217//0
SOD1	rs4816407	A>G	1.255	0.534	0.815	0.556-1.194	1.201	0.813-1.774	1.125	0.522-2.426	0.019//0.991	0.201//0.904
SOD1	rs80265967	A>C	-	-	-	-	0.000	-	-	-	2.92//0.232	-
SOD2	rs113886498	A>G	32.390	<0.0001	20.892	4.946-88.243	0.050	0.012-0.21	0.000	-	0.221//0.895	0.005//0.998
SOD2	rs117466922	A>G	-	-	0.725	0.226-2.32	1.380	0.431-4.418	-	-	0.03//0.985	0.059//0.971
SOD2	rs12190141	A>G	-	-	0.490	0.165-1.46	2.039	0.685-6.068	-	-	0.03//0.985	0.121//0.941
SOD2	rs12204454	T>C	7.009	0.030	1.483	1.012-2.174	0.595	0.405-0.875	1.268	0.739-2.176	0.294//0.863	8.496//0.014
SOD2	rs13212047	A>C	51.819	<0.0001	60.465	8.25-443.177	0.000	-	0.976	0.061-15.711	1.359//0.507	210//0
SOD2	rs140181088	A>G	0.003	0.998	0.990	0.471-2.079	1.010	0.469-2.173	1.009	0.063-16.242	1.817//0.403	1.788//0.409
SOD2	rs4516970	A>G	1.538	0.464	1.223	0.535-2.795	0.740	0.317-1.725	-	-	0.212//0.899	4.436//0.109

SOD2	rs5746081	A>G	45.825	<0.0001	7.517	3.809-14.837	0.120	0.059-0.244	-	-	5.56//0.062	4.436//0.109
SOD2	rs75374960	T>C	-	-	1.427	0.446-4.568	0.701	0.219-2.243	-	-	0.059//0.971	0.029//0.985
SOD2	rs80065806	A>C	2.564	0.278	0.573	0.28-1.17	1.805	0.864-3.771	0.986	0.061-15.865	2.676//0.262	0.254//0.881
SOD2	rs143582231	A>G	45.036	<0.0001	51.550	7.019-378.587	0.000	-	0.981	0.061-15.793	0.691//0.708	216//0
SOD2	rs2758348	T>C	5.963	0.051	1.441	0.951-2.182	0.734	0.478-1.129	0.665	0.257-1.723	0.198//0.906	0.635//0.728
SOD2	rs2842974	T>C	3.135	0.209	1.251	0.853-1.833	0.721	0.492-1.056	1.382	0.704-2.712	4.614//0.1	0.035//0.983
SOD2	rs4709364	T>C	8.939	0.011	1.377	0.934-2.031	0.577	0.387-0.86	1.587	0.877-2.871	0.251//0.882	12.553//0.002
SOD2	rs4880	A>G	7.131	0.028	1.456	0.99-2.14	0.602	0.409-0.885	1.455	0.761-2.782	5.143//0.076	1.356//0.508
SOD3	rs17880193	T>C	5.023	0.081	4.056	0.45-36.59	0.000	-	-	-	0.019//0.991	217//0
SOD3	rs17878863	A>G	2.834	0.242	1.215	0.623-2.37	0.924	0.467-1.831	0.000	-	3.633//0.163	0.408//0.815
SOD3	rs17880362	T>C	38.200	<0.0001	6.159	3.179-11.934	0.148	0.075-0.293	-	-	4.666//0.097	3.54//0.17
SOD3	rs800444	A>G	24.575	<0.0001	24.720	3.292-185.644	0.000	-	-	-	0.628//0.731	207//0
SOD3	rs368892083	A>G	60.211	<0.0001	68.211	9.313-499.572	0.000	-	-	-	3.719//0.156	217//0
SOD3	rs1799895	C>G	1.197	0.550	1.116	0.525-2.373	0.965	0.448-2.078	0.000	-	1.685//0.431	0.244//0.885
SOD3	rs8192290	T>C	-	-	2.780	1.635-4.726	0.360	0.212-0.612	-	-	4.382//0.112	0.683//0.711
SOD3	rs2536512	A>G	1.912	0.384	0.769	0.519-1.139	1.266	0.866-1.851	1.018	0.608-1.704	0.864//0.649	0.22//0.896
SOD3	rs2855262	T>C	0.604	0.740	0.898	0.604-1.334	1.035	0.706-1.516	1.126	0.675-1.876	0.145//0.93	0.139//0.933
SRXN1	rs6053666	T>C	1.298	0.523	1.199	0.788-1.824	0.812	0.553-1.192	1.115	0.654-1.899	7.33//0.026	1.76//0.415
SRXN1	rs6107696	A>G	1.885	0.390	1.463	0.765-2.797	1.029	0.703-1.507	0.844	0.572-1.245	2.829//0.243	1.304//0.521
SRXN1	rs7268200	A>G	13.818	0.001	1.840	1.254-2.7	0.668	0.455-0.981	0.247	0.08-0.756	3.494//0.174	5.462//0.065
TXNRD1	rs550114104	T>C	-	-	-	-	0.000	-	-	-	0.001//0.999	-
TXNRD1	rs117567389	T>C	7.486	0.024	1.682	1.098-2.578	0.634	0.411-0.978	0.356	0.068-1.857	1.174//0.556	1.053//0.591
TXNRD1	rs34337366	T>G	-	-	-	-	0.000	-	-	-	0.019//0.991	-
TXNRD1	rs35232644	T>C	1.507	0.471	0.792	0.21-2.992	1.690	0.399-7.164	0.000	-	33.357//0	0.03//0.985
TXNRD1	rs4411337	T>C	3.279	0.194	1.356	0.921-1.998	0.760	0.516-1.12	0.909	0.467-1.772	1.275//0.529	0.002//0.999
TXNRD1	rs61937916	A>G	-	-	1.193	0.522-2.726	0.838	0.367-1.914	-	-	0.208//0.901	0.147//0.929
TXNRD1	rs73392601	A>C	0.194	0.908	0.838	0.367-1.914	1.212	0.512-2.867	1.000	0.062-16.091	4.436//0.109	2.834//0.242
TXNRD1	rs74990047	A>G	52.641	<0.0001	63.623	8.68-466.374	0.017	0.002-0.122	0.000	-	0.347//0.841	0.001//0.999
TXNRD1	rs75300553	A>G	-	-	0.000	-	-	-	-	-	-	0.001//0.999
TXNRD1	rs77176894	T>C	-	-	0.763	0.279-2.087	1.311	0.479-3.585	-	-	0.058//0.971	0.098//0.952

TXNRD1	rs4077561	T>C	4.309	0.116	0.660	0.405-1.076	0.970	0.663-1.418	1.434	0.934-2.203	1.703//0.427	2.082//0.353
TXNRD1	rs4445711	A>G	2.522	0.283	0.545	0.253-1.175	1.051	0.711-1.552	1.115	0.762-1.631	1.018//0.601	0.397//0.82
TXNRD1	rs7301631	T>C	7.019	0.030	0.821	0.447-1.507	0.647	0.439-0.955	1.665	1.13-2.452	0.392//0.822	1.606//0.448
TXNRD1	rs7975161	T>C	15.493	<0.0001	2.385	1.535-3.707	0.442	0.281-0.696	0.412	0.105-1.617	0.24//0.887	0.319//0.853
UCP3	rs117552079	A>G	0.436	0.804	1.313	0.563-3.061	0.743	0.306-1.801	1.005	0.062-16.165	2.854//0.24	5.593//0.061
UCP3	rs1685325	T>C	4.437	0.109	1.547	0.966-2.479	0.680	0.461-1.003	1.141	0.706-1.845	16.995//0	2.133//0.344
UCP3	rs1726743	T>C	0.371	0.831	0.995	0.474-2.089	0.929	0.426-2.025	2.019	0.182-22.428	1.832//0.4	9.041//0.011
UCP3	rs76833611	T>C	1.666	0.435	2.048	0.607-6.905	0.419	0.107-1.64	0.995	0.062-16.017	9.137//0.01	33.517//0
UCP3	rs11235971	T>C	6.107	0.047	1.584	1.074-2.335	0.675	0.459-0.995	0.819	0.42-1.596	2.144//0.342	0.009//0.995
UCP3	rs138705669	T>C	51.155	<0.0001	59.268	8.085-434.46	0.000	-	0.963	0.06-15.497	1.169//0.557	217//0
UCP3	rs17848368	A>G	4.012	0.135	3.028	0.312-29.344	0.000	-	-	-	0.011//0.995	216//0
UCP3	rs183714776	T>C	1.995	0.369	4.019	0.445-36.253	0.000	-	0.333	0.034-3.23	158.559//0	215//0
UCP3	rs199679366	T>C	2.049	0.359	3.057	0.315-29.62	0.000	-	0.991	0.062-15.943	52.745//0	217//0
UCP3	rs201993988	T>C	53.561	<0.0001	62.458	8.532-457.195	0.000	-	0.986	0.061-15.868	1.481//0.477	217//0
UCP3	rs2734827	A>G	2.754	0.252	1.240	0.849-1.811	0.734	0.502-1.074	1.361	0.677-2.734	3.844//0.146	0.058//0.972
UCP3	rs45476292	T>C	3.007	0.222	4.037	0.448-36.419	0.000	-	1.005	0.062-16.165	33.677//0	217//0
UCP3	rs76629964	A>G	42.779	<0.0001	48.694	6.623-358.012	0.000	-	0.977	0.061-15.72	0.508//0.776	217//0

Note: *A – Ancestral allele, *B – Derived allele

Table S5. Allele Frequency of Significantly Associated Polymorphisms of Xenobiotic Detoxification Genes

**Gene**	**rs ID (Alleles)**	**Allele frequency in case**		**Allele frequency in control**		**χ2**	**p-valume**	**OR for A* allele**	**95% CI**	**OR for B* allele**	**95% CI**
		**Allele A***	**Allele B***	**Allele A***	**Allele B***						
CYP1A1	rs2606345 (C>A)	0.690	0.310	0.653	0.347	1.302	0.254	1.182	0.887-1.576	0.846	0.634-1.128
CYP2B6	rs2279345 (C>T)	0.680	0.320	0.601	0.399	5.583	0.018	1.409	1.06-1.873	0.710	0.534-0.944
CYP2B6	rs3745274 (G>T)	0.775	0.225	0.697	0.303	6.327	0.012	1.495	1.092-2.047	0.669	0.488-0.916
CYP2B6	rs4803417 (A>C)	0.669	0.331	0.653	0.347	0.228	0.633	1.073	0.804-1.43	0.932	0.699-1.243
CYP2B6	rs6508964 (A>G)	0.671	0.329	0.654	0.346	0.251	0.616	1.076	0.807-1.436	0.929	0.697-1.239
CYP2C19	rs4494250 (G>A)	0.710	0.290	0.671	0.329	1.466	0.226	1.199	0.893-1.61	0.834	0.621-1.119
CYP2C19	rs11592737 (A>G)	0.857	0.143	0.913	0.087	6.605	0.010	0.568	0.368-0.878	1.759	1.139-2.718
CYP2C19	rs11188092 (A>C)	0.859	0.141	0.919	0.081	7.536	0.006	0.541	0.348-0.843	1.847	1.186-2.877
CYP2C19	rs17884832 (T>G)	0.922	0.078	0.826	0.174	17.751	<0.0001	2.475	1.607-3.811	0.404	0.262-0.622
CYP2C19	rs4986893 (G>A)	0.961	0.039	0.863	0.137	25.449	<0.0001	3.861	2.211-6.742	0.259	0.148-0.452
GSTP1	rs1871042 (C>T)	0.723	0.277	0.797	0.203	6.394	0.011	0.665	0.484-0.913	1.504	1.095-2.065
GSTP1	rs4147581 (C>G)	0.512	0.488	0.597	0.403	6.391	0.011	0.707	0.54-0.925	1.415	1.081-1.853
GSTP1	rs1695 (A>G)	0.695	0.305	0.772	0.228	6.532	0.011	0.674	0.498-0.913	1.484	1.095-2.009
NAT2	rs1041983 (C>T)	0.647	0.353	0.550	0.450	8.505	0.004	1.504	1.143-1.98	0.665	0.505-0.875
NAT2	rs1208 (A>G)	0.750	0.250	0.815	0.185	5.224	0.022	0.680	0.488-0.947	1.471	1.055-2.05
NAT2	rs1799930 (G>A)	0.749	0.251	0.680	0.320	4.887	0.027	1.404	1.039-1.899	0.712	0.527-0.963
NAT2	rs1799931 (G>A)	0.898	0.102	0.795	0.205	17.629	<0.0001	2.277	1.54-3.365	0.439	0.297-0.649
Note: *A – Ancestral allele, *B – Derived allele											

Table S6. Genotype Frequency of Significant Associations of Xenobiotic Detoxification Genes

**Gene**	**rs ID**	***χ2***	***p*-valume**	**OR for AA genotype**	**95% CI**	**OR for AB genotype**	**95% CI**	**OR for BB genotype**	**95% CI**
CYP1A1	rs2606345 (C>A)	7.591	0.022	1.518	1.031-2.235	0.589	0.4-0.866	1.390	0.723-2.67
CYP2B6	rs2279345 (C>T)	17.283	0.000	2.106	1.408-3.149	0.455	0.307-0.674	1.198	0.653-2.195
CYP2B6	rs3745274 (G>T)	8.406	0.015	1.721	1.161-2.553	0.622	0.417-0.929	0.708	0.323-1.554
CYP2B6	rs4803417 (A>C)	7.020	0.030	1.356	0.919-2.001	0.618	0.418-0.913	1.528	0.83-2.813
CYP2B6	rs6508964 (A>G)	12.477	0.002	1.477	0.999-2.185	0.527	0.356-0.779	1.957	1.019-3.758
CYP2C19	rs4494250 (G>A)	13.099	0.001	1.639	1.111-2.416	0.502	0.339-0.745	1.721	0.868-3.412
CYP2C19	rs11592737 (A>G)	6.450	0.040	0.576	0.357-0.928	1.562	0.953-2.562	3.433	0.705-16.722

CYP2C19	rs11188092 (A>C)	7.147	0.028	0.546	0.336-0.887	1.650	0.997-2.73	3.450	0.708-16.803
CYP2C19	rs17884832 (T>G)	23.850	0.000	3.016	1.883-4.832	0.314	0.194-0.508	1.944	0.175-21.604
CYP2C19	rs4986893 (G>A)	29.430	0.000	4.589	2.54-8.29	0.208	0.114-0.381	1.000	0.062-16.092
GSTP1	rs1871042 (C>T)	6.885	0.032	0.658	0.447-0.968	1.271	0.857-1.886	2.514	1.021-6.195
GSTP1	rs4147581 (C>G)	6.140	0.046	0.641	0.426-0.964	1.087	0.745-1.587	1.583	0.989-2.534
GSTP1	rs1695 (A>G)	6.740	0.034	0.644	0.441-0.941	1.310	0.891-1.927	2.050	0.936-4.489
NAT2	rs1041983 (C>T)	9.000	0.011	1.795	1.205-2.675	0.743	0.508-1.087	0.646	0.387-1.078
NAT2	rs1208 (A>G)	5.712	0.058	0.642	0.432-0.954	1.425	0.948-2.14	1.793	0.701-4.588
NAT2	rs1799930 (G>A)	9.188	0.010	1.745	1.185-2.571	0.562	0.379-0.834	1.003	0.493-2.044
NAT2	rs1799931 (G>A)	19.376	0.000	2.613	1.69-4.041	0.398	0.256-0.621	0.387	0.074-2.017
Note: *A – Ancestral allele, *B – Derived allele									

Table S7. Allele Frequency of Significantly Associated Polymorphisms of Antioxidant Defense Genes

**Gene**	**rs ID**	**Allele frequency in case**		**Allele frequency in control**		**χ2**	**p-valume**	**OR for A* allele**	**95% CI**	**OR for B** allele**	**95% CI**
		**Allele A***	**Allele B****	**Allele A***	**Allele B****						
AKR1B10	rs1722883 (T>C)	0.576	0.424	0.594	0.406	0.277	0.599	0.928	0.702-1.227	1.078	0.815-1.425
AKR1C1	rs2904799 (G> A)	0.712	0.288	0.628	0.372	6.389	0.011	1.466	1.089-1.973	0.682	0.507-0.918
AKR1C1	rs2904802 (C> T)	0.813	0.188	0.752	0.248	4.551	0.033	1.426	1.028-1.978	0.701	0.505-0.972
GCLC	rs4715407 (G> A)	0.804	0.196	0.720	0.280	8.015	0.005	1.598	1.153-2.213	0.626	0.452-0.867
GCLC	rs524553 (C> T)	0.871	0.129	0.791	0.209	9.835	0.002	1.780	1.238-2.561	0.562	0.39-0.808
GCLC	rs547222 (T>C)	0.816	0.184	0.753	0.247	4.873	0.027	1.455	1.042-2.032	0.687	0.492-0.96
GCLM	rs41303970 (C>T)	0.881	0.119	0.873	0.127	0.130	0.718	1.077	0.72-1.611	0.929	0.621-1.389
GPX4	rs117193629 (C> T)	0.942	0.058	0.972	0.028	4.463	0.035	0.477	0.236-0.961	2.099	1.04-4.234
NFE2L1	rs147114188 (G> A)	0.972	0.028	0.866	0.134	33.119	<0.0001	5.454	2.884-10.312	0.183	0.097-0.347
NFE2L1	rs2023885 (G> A)	0.856	0.144	0.771	0.229	10.132	0.001	1.768	1.241-2.517	0.566	0.397-0.806
NFE2L3	rs2237329 (C> T)	0.866	0.134	0.780	0.220	10.971	0.001	1.829	1.276-2.624	0.547	0.381-0.784
NFE2L3	rs12113404 (A>G)	0.769	0.231	0.670	0.330	10.407	0.001	1.637	1.212-2.211	0.611	0.452-0.825
NQO1	rs76921462 (C> T)	0.910	0.090	0.857	0.143	5.756	0.016	1.684	1.096-2.587	0.594	0.387-0.912
NQO1	rs2917677 (C> T)	0.757	0.243	0.697	0.303	3.907	0.048	1.356	1.002-1.835	0.738	0.545-0.998
PON1	rs854568 (G> A)	0.295	0.705	0.291	0.709	0.019	0.891	1.021	0.761-1.368	0.980	0.731-1.313
PON2	rs12534274 (G> A)	0.628	0.372	0.531	0.469	8.155	0.004	1.490	1.133-1.96	0.671	0.51-0.883
PON2	rs2299267 (A>G)	0.717	0.283	0.664	0.336	2.703	0.100	1.278	0.954-1.713	0.782	0.584-1.049
PON3	rs138268669 (G> A)	0.973	0.027	0.859	0.141	35.441	<0.0001	6.037	3.119-11.683	0.166	0.086-0.321

PON3	rs17885558 (G> A)	0.938	0.063	0.865	0.135	12.392	<0.0001	2.345	1.443-3.81	0.426	0.262-0.693
SOD1	rs1041740 (C> T)	0.655	0.345	0.591	0.409	3.747	0.053	1.314	0.996-1.732	0.761	0.577-1.004
SOD2	rs12204454 (C> T)	0.663	0.337	0.629	0.371	1.076	0.300	1.159	0.877-1.532	0.863	0.653-1.14
SOD2	rs4880 (A>G)	0.687	0.313	0.657	0.343	0.844	0.358	1.144	0.859-1.524	0.874	0.656-1.165
SRXN1	rs7268200 (A>G)	0.788	0.212	0.686	0.314	11.465	0.001	1.699	1.248-2.313	0.589	0.432-0.801
TXNRD1	rs117567389 (T>C)	0.867	0.133	0.804	0.196	5.953	0.015	1.597	1.094-2.33	0.626	0.429-0.914
TXNRD1	rs7301631 (T>C)	0.291	0.709	0.363	0.637	5.049	0.025	0.718	0.537-0.959	1.393	1.043-1.861
TXNRD1	rs7975161 (C> T)	0.898	0.102	0.804	0.196	14.933	<0.0001	2.146	1.448-3.181	0.466	0.314-0.69
UCP3	rs11235971 (T>C)	0.714	0.286	0.649	0.351	4.077	0.043	1.351	1.008-1.809	0.740	0.553-0.992
Note: *A – Ancestral allele, *B – Derived allele											

Table S8. Genotype Frequency of Significant Associations of Antioxidant Defense Genes

Gene	rs ID	$\chi 2$	*p*-valume	OR for AA genotype	95% CI	OR for AB genotype	95% CI	OR for BB genotype	95% CI
AKR1B10	rs1722883 (T>C)	7.141	0.028	1.159	0.771-1.742	0.659	0.445-0.974	1.609	0.962-2.693
AKR1C1	rs2904799 (G> A)	7.268	0.026	1.556	1.046-2.314	0.812	0.544-1.211	0.577	0.314-1.059
AKR1C1	rs2904802 (C> T)	6.026	0.049	1.619	1.096-2.39	0.621	0.418-0.924	0.854	0.304-2.398
GCLC	rs4715407 (G> A)	11.464	0.003	1.876	1.261-2.791	0.557	0.372-0.834	0.693	0.268-1.793
GCLC	rs524553 (C> T)	16.487	0.000	2.203	1.456-3.334	0.421	0.275-0.643	1.690	0.399-7.162
GCLC	rs547222 (T>C)	7.150	0.028	1.641	1.102-2.443	0.621	0.414-0.931	0.783	0.279-2.201
GCLM	rs41303970 (C>T)	6.689	0.035	1.286	0.819-2.019	0.639	0.398-1.027	4.152	0.872-19.777
GPX4	rs117193629 (C> T)	5.309	0.070	0.440	0.21-0.923	2.395	1.111-5.163	0.977	0.061-15.72
NFE2L1	rs147114188 (G> A)	38.120	0.000	6.714	3.409-13.223	0.138	0.068-0.279	0.995	0.062-16.017
NFE2L1	rs2023885 (G> A)	11.179	0.004	1.955	1.301-2.938	0.548	0.362-0.828	0.404	0.103-1.585
NFE2L3	rs2237329 (C> T)	15.339	0.000	2.219	1.464-3.363	0.434	0.283-0.666	0.962	0.305-3.032
NFE2L3	rs12113404 (A>G)	19.444	0.000	2.267	1.542-3.334	0.425	0.287-0.628	1.071	0.504-2.278
NQO1	rs76921462 (C> T)	10.570	0.005	1.959	1.226-3.133	0.471	0.292-0.762	2.887	0.298-27.985
NQO1	rs2917677 (C> T)	10.578	0.005	1.743	1.189-2.557	0.529	0.36-0.78	1.437	0.601-3.437
PON1	rs854568 (G> A)	3.971	0.137	1.813	0.868-3.783	0.741	0.506-1.085	1.139	0.781-1.661
PON2	rs12534274 (G> A)	11.173	0.004	2.013	1.328-3.05	0.646	0.44-0.948	0.734	0.439-1.23
PON2	rs2299267 (A>G)	7.943	0.019	1.611	1.095-2.369	0.584	0.396-0.861	1.179	0.603-2.303

PON3	rs138268669 (G> A)	39.147	0.000	7.404	3.656-14.995	0.127	0.061-0.266	0.493	0.044-5.477
PON3	rs17885558 (G> A)	15.324	0.000	2.639	1.572-4.431	0.372	0.22-0.629	0.907	0.056-14.6
SOD1	rs1041740 (C> T)	6.539	0.038	1.654	1.116-2.453	0.651	0.445-0.952	0.911	0.52-1.599
SOD2	rs12204454 (C> T)	7.009	0.030	1.483	1.012-2.174	0.595	0.405-0.875	1.268	0.739-2.176
SOD2	rs4880 (A>G)	7.131	0.028	1.456	0.99-2.14	0.602	0.409-0.885	1.455	0.761-2.782
SRXN1	rs7268200 (A>G)	13.818	0.001	1.840	1.254-2.7	0.668	0.455-0.981	0.247	0.08-0.756
TXNRD1	rs117567389 (T>C)	7.486	0.024	1.682	1.098-2.578	0.634	0.411-0.978	0.356	0.068-1.857
TXNRD1	rs7301631 (T>C)	7.019	0.030	0.821	0.447-1.507	0.647	0.439-0.955	1.665	1.13-2.452
TXNRD1	rs7975161 (C> T)	15.493	0.000	2.385	1.535-3.707	0.442	0.281-0.696	0.412	0.105-1.617
UCP3	rs11235971 (T>C)	6.107	0.047	1.584	1.074-2.335	0.675	0.459-0.995	0.819	0.42-1.596

Note: *A – Ancestral allele, *B – Derived allele
